# Diversity and connectivity of microbial eukaryotes in High Arctic terrestrial and aquatic ecosystems

**DOI:** 10.1093/femsec/fiag096

**Published:** 2026-10-30

**Authors:** Patrick M Hooper, Warwick F Vincent, Catherine Girard, Edward J Feil, David Bass, Anne D Jungblut

**Affiliations:** Life Sciences Department, Natural History Museum, Cromwell Road, London SW7 5BD, United Kingdom; Department of Life Sciences, University of Bath, Claverton Down, Bath BA2 7AY, United Kingdom; Département de Biologie, Takuvik International Research Laboratory and Institut de Biologie Intégrative et des Systèmes, Université Laval, Québec, QC G1V 0A6, Canada; Centre d'études nordiques (CEN), Université Laval, Québec, QC G1V 0A6, Canada; Département de Biologie, Takuvik International Research Laboratory and Institut de Biologie Intégrative et des Systèmes, Université Laval, Québec, QC G1V 0A6, Canada; Centre d'études nordiques (CEN), Université Laval, Québec, QC G1V 0A6, Canada; Département de biochimie, de microbiologie et de bio-informatique, and Institut de Biologie Intégrative et des Systèmes, Université Laval, Québec, QC G1V 0A6, Canada; Department of Life Sciences, University of Bath, Claverton Down, Bath BA2 7AY, United Kingdom; Centre for Environment, Fisheries and Aquaculture Science (Cefas), Weymouth Laboratory, Barrack Road, Weymouth DT4 8UB, United Kingdom; Centre for Sustainable Aquaculture Futures, University of Exeter, Stocker Road, Exeter EX4 4QD, United Kingdom; Life Sciences Department, Natural History Museum, Cromwell Road, London SW7 5BD, United Kingdom

**Keywords:** arctic, microbial eukaryotes, protistology, oxford nanopore technologies, 18S rRNA gene, environmental microbiome

## Abstract

High Arctic ecosystems are being rapidly altered by climate change. Increased permafrost thaw and rainfall are making terrestrial landscapes more hydrologically active, increasing the input of organic and inorganic material into freshwater systems and freshening coastal marine ecosystems. Understanding the ecological implications of this environmental change requires an improved taxonomic and ecological resolution for less-studied microbial eukaryote communities. Microbial communities were investigated from freshwater, terrestrial, and coastal marine ecosystems on Ward Hunt Island, Ward Hunt Ice Shelf, and Ellesmere Island along the coastal margin of the Last Ice Area, Canadian High Arctic. For the first time, Arctic microbial eukaryotic diversity was assessed using full-length 18S rRNA gene metabarcoding sequencing. Terrestrial microbial eukaryotic richness correlated with soil moisture, extending biogeographical patterns observed in lower latitudes. A greater connectivity between terrestrial and freshwater, than to coastal communities was identified, with a predicted input of terrestrial microbial eukaryotes into the Ward Hunt Lake microplankton. As Arctic catchments become more hydrologically active, increased transport of microbial eukaryotes may strengthen connectivity and homogenization between terrestrial and freshwater ecosystems, while greater freshwater inputs may alter microbial diversity in adjacent coastal waters.

## Teaser

18S rRNA gene microbial eukaryote community assessment identified distinct connectivity between terrestrial, freshwater, and marine coastal environments in the Canadian High Arctic. As Arctic catchments become more hydrologically active, increased transport of microbial eukaryotes may strengthen connectivity and homogenization between terrestrial and freshwater ecosystems, while greater freshwater inputs may alter microbial diversity in adjacent coastal waters.

## Introduction

Across the northernmost coastline of the Canadian High Arctic, terrestrial and aquatic ecosystems are dominated by microbial life (Vincent et al. 2009). Communities of microbes, often thriving under extreme environmental conditions, drive biogeochemical cycles, primary production, and food webs across the Arctic (Vincent and Laybourn-Parry 2008). Once constrained by consistent freezing temperatures, these ecosystems are now undergoing rapid transformation due to accelerated warming (IPCC 2023, Girard et al. 2023). Increased permafrost thaw and the shift from snow to rain are making terrestrial landscapes more hydrologically active, increasing the input of organic and inorganic material into freshwater systems and ‘freshening’ adjacent coastal marine environments (Vincent 2010, Bintanja and Andry 2017). Careful monitoring of microbial diversity is therefore essential for understanding the ecological implications of environmental change in the High Arctic (Edwards et al. 2020).

Comparative 18S rRNA gene metabarcoding across temperate, tropical, and Antarctic soils demonstrated that terrestrial protist communities are strongly structured by soil moisture availability (Bates et al. 2013, Geisen et al. 2014). Increased permafrost thaw and rainfall in the Arctic may therefore lead to greater richness of terrestrial microbial eukaryotic life. For example, thawed permafrost demonstrated a three-fold abundance of heterotrophic ciliate and cercozoan taxa in comparison to frozen permafrost in Zackenberg, northeast Greenland (Scheel et al. 2023). Understanding the connectivity of microbes within freshwater and terrestrial habitats is therefore of interest in monitoring the effects of Arctic climate change (Cavaco et al. 2019, Girard et al. 2023). The movement of microbial eukaryotes between terrestrial and freshwater environments has already been observed in Arctic Lake catchments, such as at Toolik Lake (Crump et al. 2012), and Ward Hunt Lake (WHL) (Comte et al. 2018). Furthermore, the ‘freshening’ of coastal Arctic marine environments raises concerns about the loss of distinct microbial eukaryotic communities that are adapted to conditions of high salinity (Singer et al. 2021). Therefore, an increased understanding of the connectivity of microbial eukaryotic communities between terrestrial, freshwater, and coastal marine habitats is needed for improved monitoring of ecosystem change.

WHL, the northernmost lake in Canada, is situated on Ward Hunt Island (WHI) (83°N), a small island separated from Ellesmere Island by seasonally open waters of the coastal Arctic Ocean and a remnant section of the Ward Hunt Ice Shelf (WHIS) (Vincent et al. 2011). WHI is proximal to the oldest, thickest sea ice in the Arctic Ocean, the Last Ice Area (LIA), considered the final refuge for Arctic Sea ice and ice-dependent species (Vincent and Mueller 2020, Newton et al. 2021, Girard et al. 2023). The terrestrial, freshwater, coastal, and WHIS meltwater habitats on and near the island may be hydrologically and/or atmospherically connected, as indicated by some overlap in their microbial compositions (Jungblut et al. 2017 Blackburn-Desbiens et al. 2025).

In this study, the taxonomic diversity and connectivity of microbial eukaryotes between freshwater, terrestrial and costal marine communities proximal to the LIA were assessed by full length 18S rRNA gene metabarcoding using Oxford Nanopore Technologies (ONT) long-read sequencing. We hypothesized that richness of microbial eukaryotes would be highest in terrestrial ecosystems, in accord with global biogeographical studies of microbial eukaryotes. Additionally, we anticipated a greater connectivity of terrestrial and freshwater ecosystems, compared to the relative isolation of coastal marine communities.

## Materials and methods

### Study site and sampling

Environmental samples were collected from Ward Hunt Island, Nunavut, Canada (83°05′08“N; 74°09′43″W) between 16^th^ to 24^th^ July 2022 (Fig. 1A–C). Ward Hunt Island contains Ward Hunt Lake (WHL), 0.37 km^2^ in area and 9.7 m deep. WHL is mainly fed by snowmelt from the surrounding slopes (Vincent et al. 2011). Once perennially ice-covered, the ice cover on WHL has rapidly thinned (Paquette et al. 2015). Subsurface permafrost forces water to form routes downstream through the active layer, creating zones of high soil moisture, soil crusts, surrounded by comparatively dryer soils (Steven et al. 2013). In the summer months, snowmelt forms seasonal water tracks on the soil surface which feed into the lake (Paquette et al. 2017, 2018). On the northern coast of the island, a once freshwater proglacial bay, unofficially named Quttinirpaaq Lagoon (QL), is now hydrologically connected to the Arctic Ocean, due to the collapse of a section of the Ward Hunt Ice Shelf (WHIS) in 2008 (Vincent et al. 2009, 2011). Samples were further collected from two sites on nearby northern Ellesmere Island, a stream flowing into Lake A (LA) (82°53′27″N, 75°26′24″W) and a small pond, informally named Antoniades Pond (AP) (82°58′48″N, 75°24′00″W) and from two meltwater ponds on the Ward Hunt Ice Shelf (83°05′03″N, 74°22′22″W).

**Figure 1:**
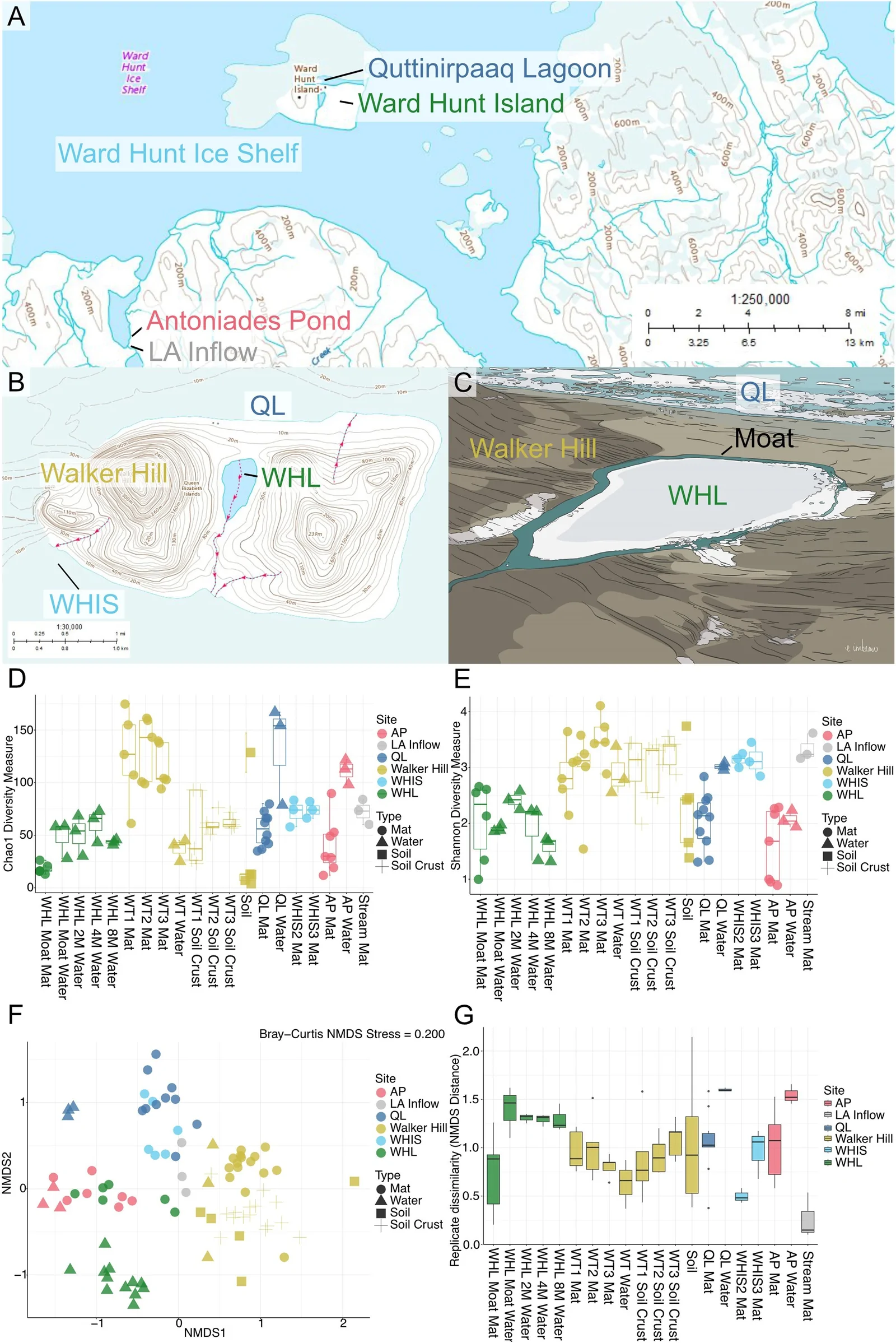
Summary of microbial eukaryotic diversity across sampling locations. (A) Map of Ellesmere Island and (B) Ward Hunt Island (Natural Resources Canada 2021). (C) Illustration of Ward Hunt Lake (WHL), the eastern slope of Walker Hill, and Quttinirpaaq Lagoon (QL), Ward Hunt Island, Nunavut Canada (83°N), adapted from original illustration by E. Imbeau. Estimates of microbial eukaryotic (protists and fungi) gene richness (D) (Chao1 diversity index) and (E) evenness (Shannon diversity index). (F) Ordination of the relative abundance transformed microbial eukaryotic community. (G) Euclidean distance of Bray–Curtis dissimilarity values within sampling locations. AP (Pink) = Antoniades Pond, LA Inflow (Grey) = Lake A inflow stream, QL (Dark Blue) = Quttinirpaaq Lagoon, WHIS (Light Blue) = Ward Hunt Ice Shelf, Walker Hill (Yellow), WHL (Green) = Ward Hunt Lake.

Microbial communities sampled are summarized in Suppl. Table 1. Microbial mat samples were collected from the moat of WHL, WHIS, QL, AP, LA, and the water tracks (WT_M). All freshwater and coastal microbial mats were sampled from the shoreline at ∼15–30 cm depth using a sterile stainless-steel spatula. Microplankton samples were collected at depths of 0.25, 2, 4, and 8 m from WHL using a sterilized Kemmerer bottle through a bore hole, through ∼155 cm of surface ice. WHL moat and QL microplankton samples were collected at 30–50 cm depth, 8 m from the shoreline. From each sample, 1000–1800 ml of water was filtered through 0.22 μm Millipore® Sterivex™ Pressure Filters (Merck, Germany) using a Masterflex™ Digital Drive peristaltic pump (Fisher Scientific, UK). For Antoniades Pond (AP) and two of the water tracks (WT_W), 240–360 ml of water was filtered through a 0.22 μm Millipore® Sterivex™ Pressure Filter using a sterile 60 ml syringe. Terrestrial microbial mat communities were collected along three water tracks at the bottom of Walker Hill, leading into WHL. Five surface soil crust (WT_C) and five sub-surface soil (WT_S) samples (sampled at a depth of 10–15 cm) were from collected three transects parallel to the water tracks. All samples were stored at −20°C at the field research station on WHI, before being transported to the Natural History Museum, London, UK.

At each microbial mat and water sampling site, water temperature, pH and specific conductivity were measured using a pH/Con 10 Series probe (Oakton Instruments™, UK). Soil and soil crust dry weight, pH, and specific conductivity were measured in the NHM laboratory on a SevenExcellence pH/Cond meter S470 (Mettler Toledo, UK), following standard protocols of the Global Soil Laboratory Network (GLOSOLAN 2021a,b). Profiles of water temperature, specific conductivity, dissolved oxygen concentration, and Chlorophyll ɑ were measured using a Ruskin RBR Concerto profiler at the deepest part of WHL, through a hole drilled in the surface ice (RBR, Ottawa, Canada). Concentrations of total nitrogen (TN), total phosphorus (TP), dissolved organic carbon (DOC), total inorganic carbon (TIC), total dissolved nitrogen (TDN), and anion and cation concentrations were measured from water samples collected at Ward Hunt Lake and Antoniades Pond. Anions were also measured in Quttinirpaaq Lagoon. All samples were processed at Institut national de la recherche scientifique (INRS), Quebec, Canada.

### DNA extraction

DNA was extracted from 0.1–0.2 g of microbial mat samples and soil and soil crust samples using the DNeasy PowerBiofilm Kit and DNAeasy PowerSoil Pro Kit (QIAGEN, UK), respectively following manufacturer’s instructions. Microplankton DNA extractions were processed following a protocol adapted from Cruaud et al. (2017) and Fournier et al. (2021). Briefly, the Sterivex filter papers are cut into pieces and incubated in 600 μl of 1% β-mercaptoethanol (Sigma-Aldrich, UK) in RLT+ buffer (QIAGEN, UK) and 50 μl of Lysozyme (0.04%) (Sigma–Aldrich, UK), at 37°C for 45 min with horizontal stirring at 200 r/m. After 45 min, 85 μl of sodium dodecyl sulfate (10%) and 30 μl of proteinase K (8 mg/ml) are added to the microcentrifuge tube and incubated at 65°C for 15 min, with horizontal stirring at 200 r/m. The sample tubes are vortexed for 4 min and the lysate is spun through a QiaShredder (QIAGEN, UK) column. The flowthrough is transferred to an AllPrep DNA column from the AllPrep DNA/RNA co-extraction kit (Qiagen, UK) and eluted in EB buffer, following manufacturer’s instructions. All DNA extractions were immediately stored at −20°C after processing.

### Polymerase chain reaction (PCR) and gel electrophoresis

DNA samples were amplified in triplicate to reduce potential amplification bias (Polz and Cavanaugh 1998). The V1–V9 regions of the 18S rRNA SSU rRNA (ca 1794 bp) were amplified using the NSF4/18 (CTG GTT GAT YCT GCC AGT) (Hendriks et al. 1989) and EukR/B (TGA TCC TTC TGC AGG TTC ACC TAC) (Medlin et al. 1988) primer set in a PCR reaction mix consisting of: 4 μl 1X buffer (Promega, UK), 2 μl 2.5 mM MgCl_2_ (Promega, UK), 1 μl 0.2 mG recombinant albumin (New England Biolabs®, UK), 0.16 μl 0.25 mM dNTPs (Meridian BioScience®, UK), 1 μl 0.5 mM forward and reverse primers, HyClone™ HyPure™ Molecular Biology Grade Water (Nuclease-Free) (Avantor, UK), and 0.2 μl 1X GoTaq® G2 Flexi DNA Polymerase (Promega, UK) to a 20 μl reaction volume. The reaction cycle consisted of 94°C for 3 min, 30 cycles of 94°C for 45 s, 55°C for 1 min, 72°C for 2 min, 72°C for 5 min. Total 15 μl of the triplicate PCR products were pooled to a total volume of 45 μl per sample. Pooled PCRs were cleaned using the AppMag Bead Clean Up kit (Appleton Woods Ltd, UK), following manufacturer’s instructions. Magnetic beads were added at a 0.8× ratio to remove small DNA fragments. Purified DNA was resuspended in 40 μl of molecular grade water and quantified using the Qubit™ 4 fluorometer with BR Assay kit (Invitrogen™, UK). Sequencing libraries were prepared using the ONT Native Barcoding Ligation kit (SQK-NBD114.96). The two barcoded sequencing libraries were loaded onto individual MinION Flow Cells v10.4.1 (FLO-MIN114) (Oxford Nanopore Technologies, UK) on a GridION Mk1 device with MinKNOW v22.07.9 software and sequenced for 72 h, with a minimum read length requirement of 200 bp and super-accurate basecalling using *Guppy* v6.3.9.

### Bioinformatic analyses

Sequences were demultiplexed and trimmed of barcodes and adapters in *Guppy* v6.3.9. Raw reads were trimmed of any remaining sequencing adapters and barcodes using *PoreChop* (v0.2.4) (Wick et al. 2017). Sequences were filtered using *Chopper* (v0.7.0) (De Coster and Rademakers 2023), reads 1000–3000 bp in length and with Phred quality score >9 were retained. Filtered reads were taxonomically annotated using the *Emu* package (v3.4.5) with default settings (Curry et al. 2022). The 18S rRNA gene reads were annotated with the PR^2^ database (v5.0.0) (Guillou et al. 2013). Bacteria and Archaea assigned reads were removed in *phyloseq* (v1.48.0) (McMurdie and Holmes 2013). After filtering, median read length was 1778 bp. Emu taxonomic annotation of the 18S rRNA gene reads identified 1136 eukaryotic taxa, including 716 protist, 225 fungal, 133 metazoa, and 62 land plant taxa, across 88 samples. All samples demonstrated adequate sequencing depths, except for one soil sample, WT3_S2, which was retained in downstream analyses despite its comparatively low read depth to preserve spatial representation across the three water-track transects (Suppl. Fig. 1, Suppl. Table 1).

Data visualization and statistical analyses were conducted using the *phyloseq* and *vegan* packages in R (v2.6.4) (Oksanen et al. 2023). Chao1(richness) and Shannon (richness and evenness) indices and Bray–Curtis NMDS of protist and fungi were estimated based on relative abundance transformed protist and fungi counts. Dissimilarity within replicate communities from sampling locations was estimated by calculating the Euclidean distance of the NMDS1 and NMDS2 coordinates. Canonical Correspondence Analyses (CCA) of the microbial mat and microplankton communities included water temperature, pH, and conductivity as significant environmental variables (*P*-value < 0.001), tested using PERMANOVA with Bray–Curtis dissimilarities and 999 permutations. Samples lacking these variables were excluded, leaving 54 samples. The number of shared protist and fungi taxa between freshwater, terrestrial, and coastal environments and between the sampling sites were presented on Venn diagrams. Relative abundance of metazoa and fungi reads within the 18S rRNA gene reads was represented at the class level in separate barplots. Protist taxa were classified by trophic function as: autotrophic phototrophs and mixotrophs, heterotrophic bacterivores, eukaryvores and omnivores, pathogens, or parasites, by previous observed trophic descriptions for each taxon, as previously described in Hooper et al. (2024). Trophic descriptions of protists were presented as a relative abundance barplot for each sampling location. Metazoa and land plant assigned taxa were removed for diversity and statistical analyses

Sourcetracker2 (v1.0.0) (Knights et al. 2011) was used to ascertain the input of protists and fungi from terrestrial to freshwater and marine microbiomes. The Walker Hill samples were identified as source communities and the Ward Hunt Lake and Quttinirpaaq Lagoon samples were assessed separately as sinks. Replicate samples were grouped by sample type and analyzed using the Gibbs algorithm with 100 burn-ins. The ‘*-per_sink_feature_assignments*’ argument was used to estimate the contribution of each source community to the total read counts of the individual taxa in the sink communities.

## Results

### Environmental conditions

Highest water temperatures were measured in Antoniades Pond (6.1–9.6°C) and lowest in Quttinirpaaq Lagoon (0.8–2.9°C) and WHIS meltwater ponds (0.9–2.4°C) (Suppl. Table 2). Specific conductivity was highest in coastal marine Quttinirpaaq Lagoon (2080–4400 μS cm^1^) indicating brackish conditions, while freshwater systems ranged from 100 to 431 μS cm^−1^. TN and TP were higher in Antoniades Pond than Ward Hunt Lake (WHL) (Suppl. Table 2). Within WHL, water temperature and specific conductivity were lower in the moat region than the deeper ice-covered waters. The lake profile showed that temperature was highest at ∼5 m depth and dissolved oxygen saturation and chlorophyll α were highest at ∼7.5 m depth (Suppl. Fig. 2). Soil crust samples had higher specific conductivity (229–774 μS cm^−1^) and gravitational water content (32%–180%) than the underlying soil (88–174 μS cm^−1^, 13.5%–27.2%).

### Alpha and beta diversity of microbial eukaryotes

Highest richness of protist and fungi assigned 18S rRNA gene reads were in the water track microbial mats and microplankton community of Quttinirpaaq Lagoon (Fig. 1D). Across Ward Hunt Lake, Quttinirpaaq Lagoon and Antoniades Pond, richness was higher in the microplankton than microbial mat communities. However, measured diversity accounting for richness and evenness was found to be more similar between microplankton and microbial mat communities from the same water body (Fig. 1E). Walker Hill microbial eukaryotic communities were distinct from all freshwater and coastal communities (Fig. 1F). WHIS microbial mats were more similar in composition to the coastal QL microbial mats than other freshwater microbial mats. Microbial mats, soil crust, and soil communities had higher dissimilarity between replicates than the microplankton replicates (Fig. 1G). The CCA showed opposing conductivity and temperature gradients, indicating a negative correlation between these variables (Suppl. Fig. 3). PERMANOVA identified significant associations (*P >* 0.001) between community composition in the microbial mat and microplankton communities and temperature (${{R}^2} = 0.082$), pH (${{R}^2} = 0.066$) and conductivity (${{R}^2} = 0.049$). Quttinirpaaq Lagoon communities were associated with higher conductivity and lower temperature, whereas Antoniades Pond and Ward Hunt Lake communities were generally associated with higher temperature and lower conductivity. WHIS communities were associated with higher pH.

### Taxonomic and trophic diversity

Microbial eukaryotic communities were dominated by Ciliophora, Chlorophyta, Dinoflagellata, Cercozoa, and Chrysophyceae (Fig. 2). Description of trophic function was not achieved for 26 out of 716 protist taxa (Suppl. Table 3). Overall, freshwater and coastal marine microbial communities contained a higher abundance of phototrophic and mixotrophic protists than terrestrial communities, which were dominated by heterotrophic protists (Fig. 3). However, distinctions in the diversity and function of protists were identified between microbial communities within these sampling sites (Figs. 2 and 3).

**Figure 2:**
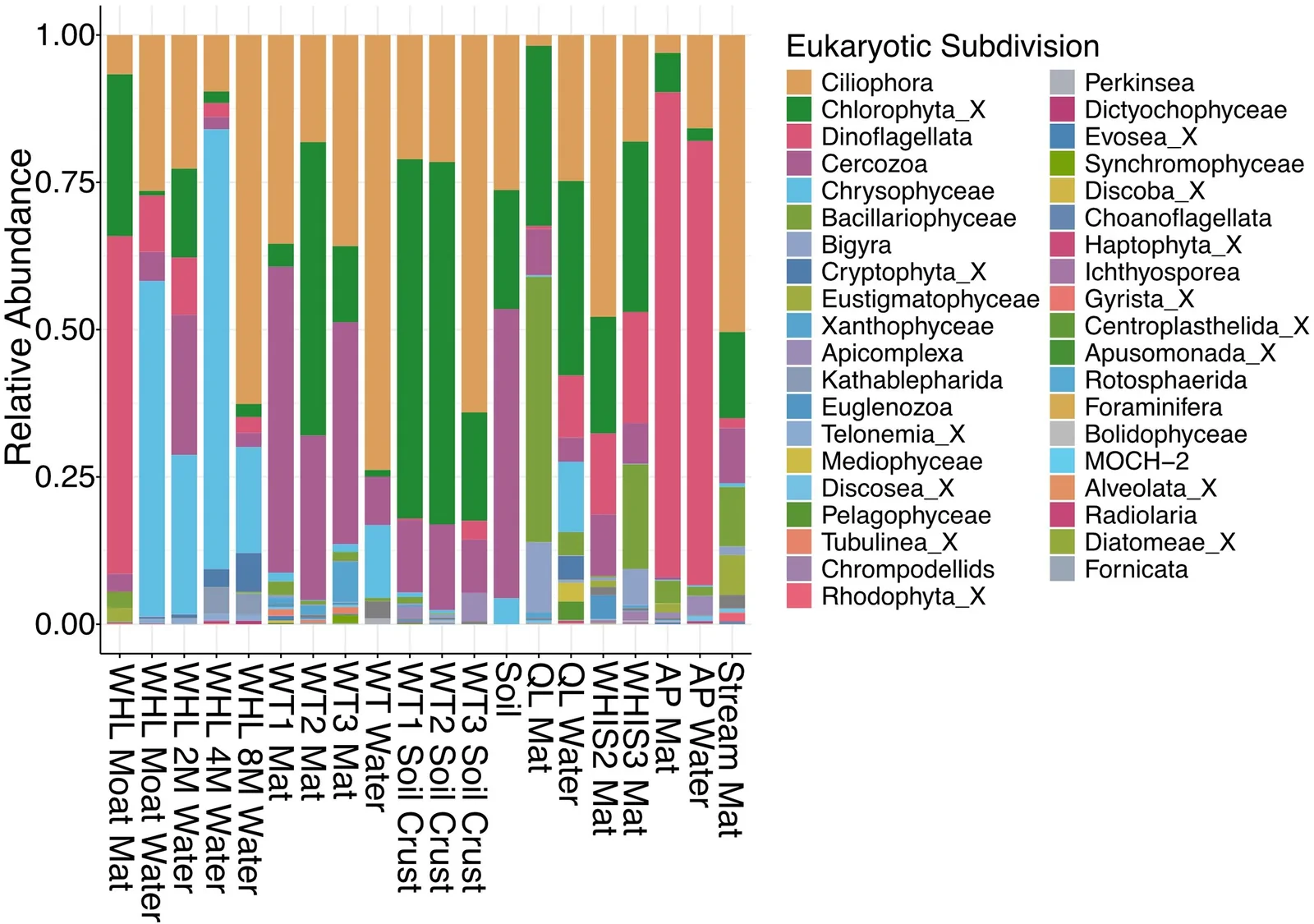
Taxonomic diversity of protists averaged by sampling location and represented as relative abundance of protist-assigned reads. Reads grouped by eukaryotic subdivision, except for the Gyrista, which are represented at the Class level. WHL = Ward Hunt Lake, WT = Water Track, QL = Quttinirpaaq Lagoon, WHIS = Ward Hunt Ice Shelf, AP = Antoniades Mat, Stream_Mat = Lake A Inflow Stream.

**Figure 3:**
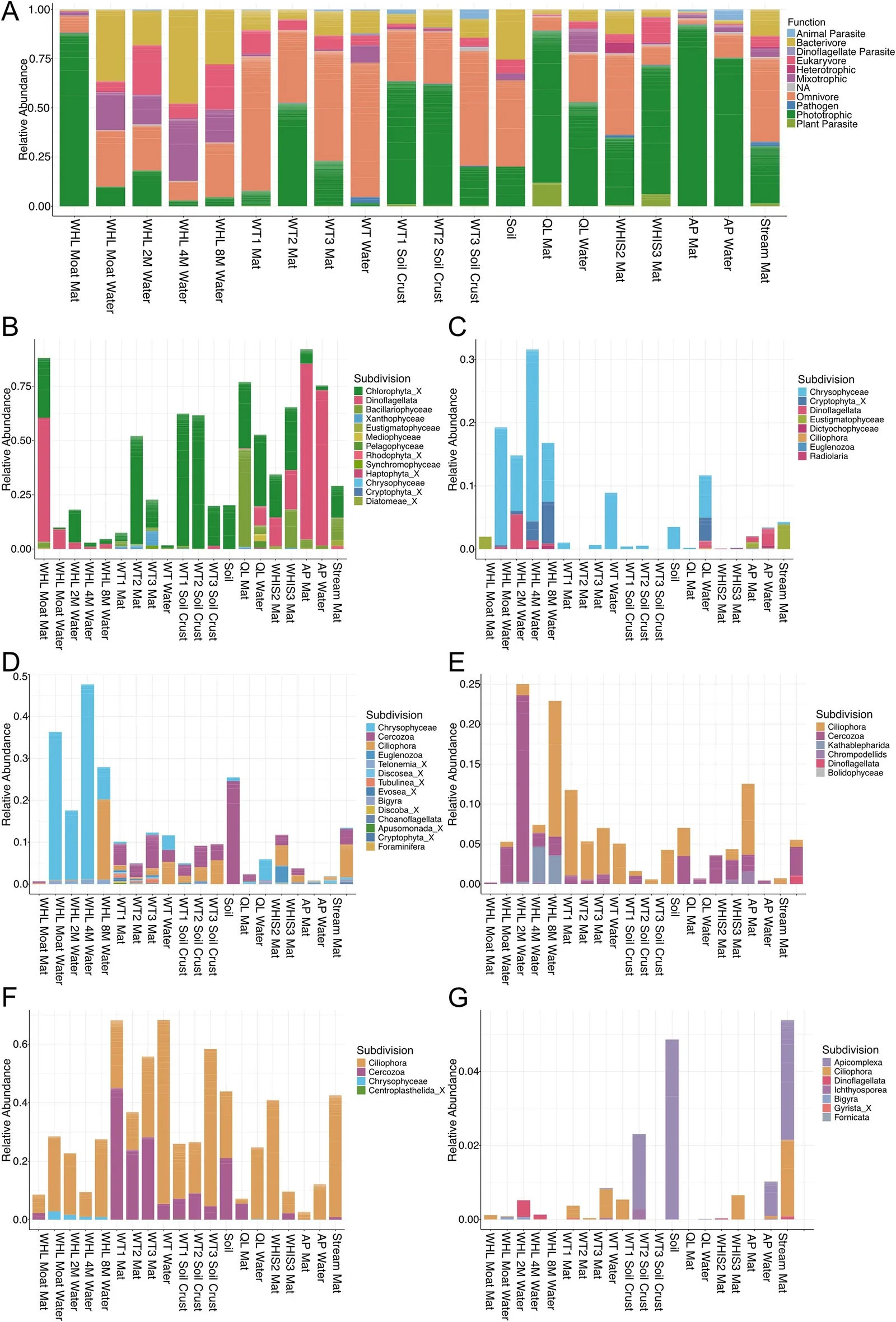
Trophic functional diversity of protists (A) averaged by sampling location and represented as relative abundance of protist-assigned reads. Within each function, taxonomic diversity was summarized at the eukaryotic subdivision level, except for Gyrista, represented at the Class level for (B) phototrophic, (C) mixotrophic, (D) bacterivore, (E) eukaryvore, (F) omnivore, and (G) animal parasite protists.

Terrestrial microbial communities were dominated by cercozoans and ciliates (Figs. 2 and 3). This included a high abundance of omnivorous *Rhogostoma* (Cercozoa) and Oxytrichidae (Ciliophora) species across the water track microbial mat and microplankton, soil crust and soil communities (Fig. 3F). Bacterivores were further identified from several eukaryotic subdivisions in the water track microbial mats (Fig. 3D). Eukaryvores including *Obertrumia georgiana, Platyophrya*, and *Nassulida* species (Ciliophora) and Vampyrellida species (Cercozoa) were abundant in the terrestrial communities (Fig. 3E). The soil crusts contained a higher abundance of phototrophic Chlorophyta than the soil communities (Fig. 3B). Pezizomycotina (Ascomycota) were also highly abundant in the soil crust and water track microbial mats (Suppl. Fig. 4A).

In Ward Hunt Lake, microbial mats were dominated by phototrophic Dinoflagellata and Chlorophyta, with the most abundant affiliated to the gymnodinoid dinoflagellate *Borghiella tenuissima* (Fig. 3B). The most abundant heterotrophic taxa were the omnivorous ciliates, *Halteria grandinella* and *Stentor elegans* (Fig. 3F). In contrast, the microplankton of Ward Hunt Lake was dominated by heterotrophic and mixotrophic Chrysophyceae, the most abundant being the obligately heterotrophic species *Spumella bureschii*. Ciliate bacterivores, eukaryvores, omnivores, and the cercozoan eukaryvores, *Protaspa* were also abundant in the microplankton community. One Rozellomycota species accounted for 99% of fungal reads in the Ward Hunt Lake microplankton (Suppl. Fig. 4A). Microbial mats in Antoniades Pond contained the highest abundance of animal parasites of all freshwater environments, mostly the invertebrate parasitic group Gregarinomorphea (Apicomplexa) (Fig. 3H). The WHIS meltwater ponds contained phototrophic dinoflagellates, including *Polarella glacialis*, and Chlamydomonadales (Chlorophyta) and and heterotrophic protists, including Eupetalomonads (Discoba), *Obertrumia georgiana, Naxella paralucida*, Vampyrellida species, and *Alphamonas* (Chrompodellids) and a *Perkinsea* parasite of dinoflagellates. Nematodes and tardigrades were shared between all freshwater microbial mats, while Antoniades Pond also contained arthropods and Lake A also contained platyhelminths in microbial mats (Suppl. Fig. 4B). Metazoan diversity was low in all microplankton communities, but rotifers were identified in Ward Hunt Lake.

The coastal marine Quttinirpaaq Lagoon microbial mats and microplankton contained 77 and 70 phototrophic taxa, respectively, however only 24 were shared in both communities. Phototrophic taxa in the microbial mats were mostly diatoms (Bacillariophyceae) and Chlorophyta, the most abundant being *Navicula phyllepta*. Comparatively, the microplankton community was dominated by *Carteria* (Chlorophyta) (Fig. 2). Heterotrophic protists were more abundant in the microplankton than the microbial mats. The microbial mats were dominated by Cercozoa, including *Vanella*, Vampyrellida and Cryomonadida species, whereas the microplankton were dominated by *Spumella* (Chrysophyceae), *Protaspa* and *Cryothecomonas* (Cercozoa) species (Fig. 2). The microbial mats also contained a high abundance of Labyrinthulomycetes (Bigyra), parasites of algal species.

### Connectivity of protists across freshwater, terrestrial, and coastal ecosystems

The microbial communities from terrestrial and freshwater environments shared more taxa with each other, than with the coastal microbial communities (Fig. 4A). Eighty microbial eukaryotes were shared by all three habitats, ∼10% of total present diversity (Suppl. Table 4). The highest abundance of unique taxa was in the terrestrial microbial communities (Fig. 4B). Twenty-four protists were identified in all sampling sites, ∼3% of present diversity (Fig. 4B). This included phototropic *Chlamydomonas* (Chlorophyta), heterotrophic Glissomonadida (Cercozoa), hypotrich ciliates, and the cold-water dinoflagellate *Polarella glacialis*, one Rozellomycota and two Chytridiomycota taxa (Suppl. Table 5).

**Figure 4:**
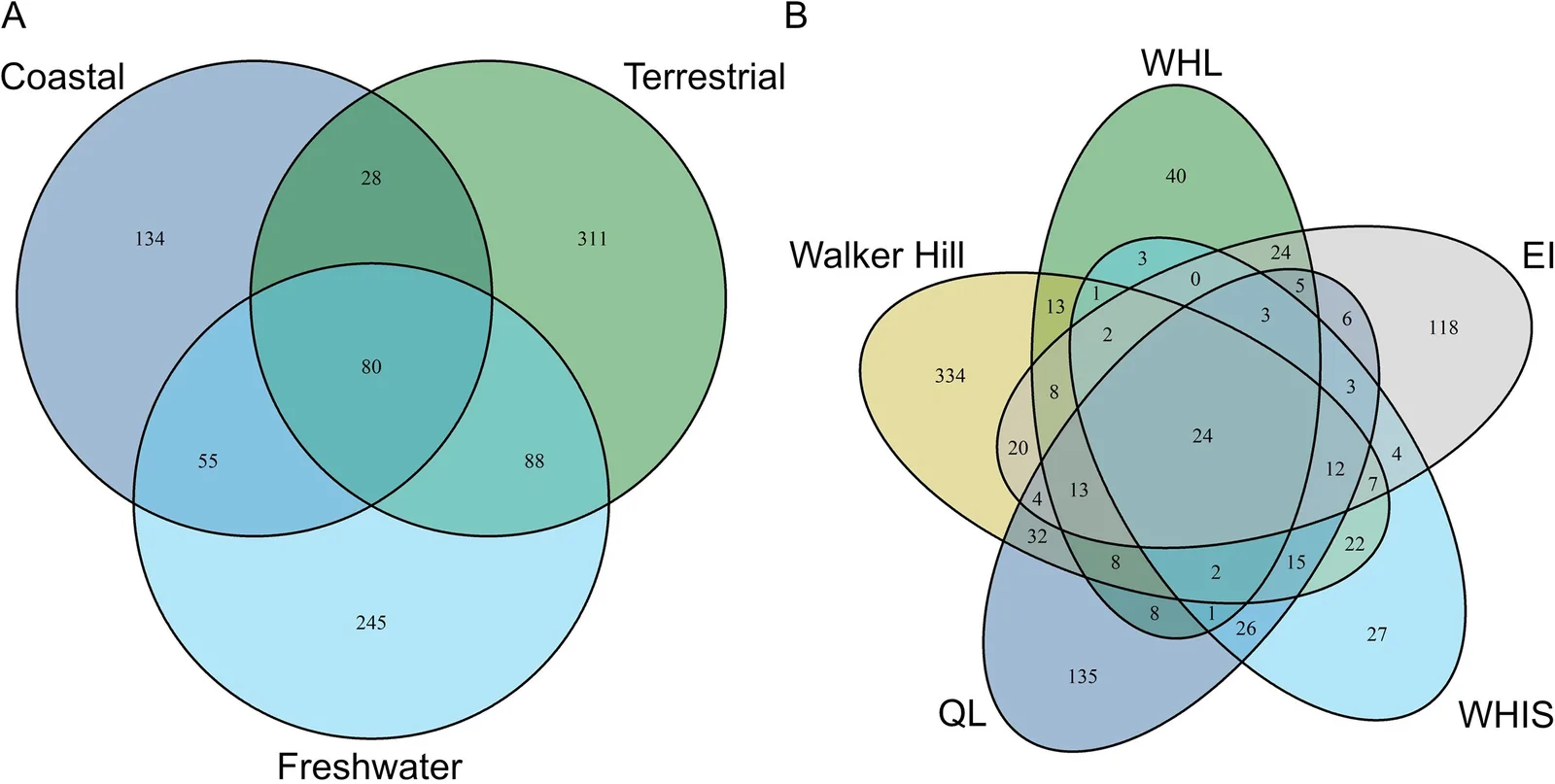
Venn diagram of shared and unique microbial eukaryotic (protist and fungi) taxa between: (A) the freshwater, terrestrial, and coastal environments and (B) the different sampling sites. WHL = Ward Hunt Lake, EI = Antoniades Pond and Lake A inflow stream, WHIS = Ward Hunt Ice Shelf, and QL = Quttinirpaaq Lagoon.

SourceTracker2 estimated 54% of the microplankton and 6% of microbial mats in WHL were contributed from terrestrial soil communities, including Rozellomycota, Cercozoa, Ciliophora, and Chrysophyceae (Table 1, Suppl. Table 6). Soil crusts and water track microbial mats influenced 17.5% and 4% of the Quttinirpaaq Lagoon microbial mats, respectively, including Chlorophyta, Rozellomycota, Pezizomycotina, Ciliophora, and Cercozoa (Suppl. Table 1, Suppl. Table 7). The terrestrial communities had no measurable influence on the Quttinirpaaq Lagoon microplankton.

**Table 1:** Contribution of protist and fungi assigned reads from source (column names) to sink (row names) microbial communities as a percentage of the total reads in the sink community.

	Soil	Soil Crust	Microbial Mat	Water	Unknown
**Ward Hunt Lake Microplankton**	52.61	0.00	0.00	0.00	47.38
**Ward Hunt Lake Microbial Mat**	6.14	0.36	0.24	0.14	93.13
**Quttinirpaaq Lagoon Microplankton Water**	0.00	0.00	0.00	0.01	99.98
**Quttinirpaaq Lagoon Microbial Mat**	1.30	17.46	4.39	0.62	76.23

Unknown refers to reads that were not identified as sourced from any of the source communities. WHL = Ward Hunt Lake, QL = Quttinirpaaq Lagoon.

## Discussion

### Hotspots of microbial eukaryotic diversity in the High Arctic

In this study, microbial eukaryotic diversity was assessed by long read 18S rRNA gene metabarcoding of terrestrial, freshwater, and coastal communities proximal to the Last Ice Area (LIA). The application of near full length 18S rRNA gene improved taxonomic classification through the inclusion of phylogenetic information from multiple variable regions (Jamy et al. 2020). Microbial eukaryotic communities of varying richness, composition, and trophic diversity were identified across terrestrial, freshwater, and coastal marine biomes, with distinct microbial communities further identified within each biome. Terrestrial microbial communities were dominated by heterotrophic protists while aquatic systems contained a high abundance of phototrophic taxa alongside diverse heterotrophic protists, extending patterns of trophic composition identified in terrestrial and aquatic communities in global protist studies (Geisen et al. 2018, Oliverio et al. 2020, Singer et al. 2021).

The richness of microbial eukaryotes in the water tracks microbial mats and coastal microplankton suggests that similar patterns of protist biogeography may be present in the High Arctic region to those identified in global biogeography studies of protists, where higher richness was identified in soil and marine, than freshwater environments (Geisen et al. 2018, Singer et al. 2021). However, within the extreme environmental conditions of the Arctic, distinct microbial communities of higher richness are found due to local scale variations in abiotic factors, particularly water availability. This highlights the importance of liquid water for facilitating life in the polar regions, as well as its critical importance for microbes as a transport medium (Vincent and Laybourn-Parry 2008, Harding et al. 2011).

### Microbial eukaryotes in High Arctic microbial communities

Arctic freshwater ecosystems commonly contain two distinct microbial communities, an oligotrophic microplankton community and dense multiannual benthic microbial mat communities (Villeneuve et al. 2001). The three-dimensional microbial mats develop over many years creating a more stable microhabitat, in which microbial eukaryotes can develop more consistent population sizes over longer periods (Jungblut et al. 2012, Hooper et al. 2024). Accordingly, the microbial eukaryotic community within the microbial mats from both Ward Hunt Lake (WHL) and Antoniades Pond (AP) showed greater diversity when both richness and evenness were considered, than the microplankton communities in each respective sampling location, suggesting the microbial mats are a more stable microbial community than the microplankton. In contrast, the microplankton are transient microbial populations, restricted for much of the year by freezing temperatures, darkness and a less stable nutrient supply (Charvet et al. 2014).

Microbial mats in WHL, AP, and the Lake A stream inflow (LA) were dominated by phototrophic protists, however phototrophic diversity differed between freshwater ecosystems. This suggests a consistent functional contribution of protists to microbial mats, despite localized distinctions in taxonomic diversity due to local scale variations in abiotic conditions between freshwater ecosystems (Comte et al. 2016, Burki et al. 2021). Because metazoan microfauna are multicellular, each organism contributes many more copies of the 18S rRNA gene than a single-celled protist, disproportionately increasing the number of metazoan reads recovered. This overrepresentation may have reduced the detection of less abundant microbial eukaryotes in the microbial mat communities, as previously described (Jungblut et al. 2012).

The dominance of heterotrophic protists throughout the WHL water column may reflect multiple factors, including reduced light availability at depth and the limited presence of metazoan grazers other than rotifers, as previously recorded (Jungblut et al. 2012, Bégin et al. 2020). The higher abundance of mixotrophic than phototrophic protists at all depths of WHL, highlights the role of mixotrophs in primary production in High Arctic lakes (Charvet et al. 2012, 2014). The abundance of autotrophic protists decreased with depth, demonstrating the impact of ice cover and water depth on light availability measured by Bégin et al. 2020, and the modulating effect this has on the microbial eukaryotic community. Rozellomycota species were highly abundant and likely play an important role in decomposition and recycling of cyanobacteria and microbial eukaryotes in the microplankton, facilitating nutrient turnover in the oligotrophic conditions (Marchetta et al. 2023). However, Rozellomycota also includes the microsporidia, diverse invertebrate and protist parasites (Bass et al. 2018). Further phylogenetic study of the Rozellomycota taxa is needed to better elucidate the ecological diversity within this group.

Situated on Ellesmere Island, Antoniades Pond and Lake A are less isolated than Ward Hunt Island, separated by sea and WHIS (Vincent et al. 2020). AP has a greater influence of terrestrial vegetation and animals, as indicated by a local population of nesting birds and animal droppings proximal to the pond. Accordingly, total phosphorus and total nitrogen concentrations were higher in Antoniades Pond than in Ward Hunt Lake. Therefore, the higher richness of protists identified in Antoniades Pond in comparison to Ward Hunt Lake was likely facilitated by greater allochthonous nutrient input from a more active terrestrial environment (Wrona et al. 2016). Correspondingly, Antoniades Pond had a high abundance of invertebrate parasites, Gregarinomorphea (Apicomplexa), alongside potential crustacean hosts, not identified in Lake A or Ward Hunt Lake (del Campo et al. 2019).

Microbial eukaryotic richness was greatest in the terrestrial samples with highest water availability. The highest richness of microbial eukaryotes across all sampling locations was identified within the water track microbial mats and soil crusts, distinctive features of polar terrestrial ecosystems (Levy et al. 2011, Steven et al. 2013). The higher abundance in the terrestrial communities with higher water content, suggest terrestrial protist taxa may be highly responsive to changes in water availability, as previously described in temperate soil communities (Adl and Coleman 2005, Bates et al. 2013, Geisen et al. 2014). Correspondingly, the lower diversity of microbial eukaryotes in deeper soils was likely limited by prolonged periods of freezing (Scheel et al. 2023). The identified high diversity of rare microbial eukaryotic species identified in the water track microbial mat and soil crust communities, not identified in the subsurface soil communities, suggests that a high diversity of moisture-limited taxa may be present in frozen terrestrial Arctic environments (Geisen et al. 2018, Oliverio et al. 2020). Therefore, within the Arctic region, terrestrial ecosystems may represent significant hotspots of microbial eukaryotes, currently limited by frozen temperatures. Accordingly, this suggests potential greater abundance of heterotrophic protists in High Arctic terrestrial microbiomes with permafrost melt, as previously experimentally demonstrated by Scheel et al. (2023). Many of the abundant terrestrial Cercozoa taxa did not have detailed taxonomic description, suggesting diverse novel taxa are contained within Arctic terrestrial ecosystems, as previously described in global soil environments (Geisen et al. 2018).

The soil crust communities contained a high abundance of the lichen-associated Chlorophyta species *Pseudostichococcus monallantoides* and *Stichococcus bacillaris* and lichen-forming Pezizomycotina fungi, which are common in early stages of succession in Arctic soil development, enhancing nutrient and water acquisition (Sanders and Masumoto 2021). The high abundance of lichen-associated algae and fungi suggests microbial eukaryotes are important for primary succession and primary production in soil crust communities on Ward Hunt Island (Steven et al. 2013), and across polar terrestrial ecosystems (Levy et al. 2011).

Distinct microbial communities were identified between the microplankton and benthic microbial mat samples from the coastal waters of the Quttinirpaaq Lagoon (QL). More specifically, the microplankton was dominated by marine protists, with no identified influence of terrestrial ecosystems on the microplankton community. In comparison, the microbial mats were shown to be more influenced by terrestrial environments, with a high abundance of terrestrial ciliate and cercozoan species, not identified in the microplankton. This suggests that following the loss of the separation of the once freshwater lagoon from the Arctic Ocean in 2008, the microplankton community in Quttinirpaaq Lagoon has been largely displaced with marine taxa input from the Arctic Ocean, while the sediment microbial mats have retained a stronger influence of terrestrial and freshwater taxa. *Carteria* (Chlorophyta), *Cryothecomonas* and marine stramenopiles (MAST) identified in the Quttinirpaaq Lagoon microplankton are important contributors to biogeochemical systems in the Arctic Ocean (Comeau et al. 2011, Thaler and Lovejoy 2015). The most abundant phototrophic protist in the QL microbial mats, Navicula phyllepta, is commonly identified in phytobenthic biofilms from brackish and marine sediments (Vanelslander et al. 2009), suggesting that the benthic communities have also responded to increased salinity, a well known environmental filter for microbial community structure (Blais et al. 2022). A high abundance of Labyrinthulomycetes (Bigyra) was also observed, which are parasites of marine algae and seagrasses that may contribute to nutrient turnover (Raghukumar 2002).

Despite being freshwater systems, meltwater ponds shared a high proportion of microbial eukaryotes with Quttinirpaaq Lagoon, suggesting the meltwater ponds are inhabited by microbes moved by wind from the proximal lagoon and Arctic Ocean. The most abundant phototrophic taxon, *Polarella glacialis*, is a marine species of cold-adapted dinoflagellate that is broadly distributed across the polar region due to its capability for cyst formation (Montresor et al. 2003). The presence of diverse bacterivores, eukaryvores and saprotrophic pythium species, suggest heterotrophic microbial eukaryotes are integral to nutrient turnover in isolated meltwater ponds (Jungblut et al. 2017). The richness of microbial eukaryotes within even small meltwater ponds demonstrates the potential biodiversity loss that is anticipated due to loss of ice-based ecosystems in the LIA (Vincent and Mueller 2020).

### Connectivity of microbial eukaryotes across High Arctic terrestrial, freshwater, and coastal ecosystems

The greater connectivity identified between terrestrial and freshwater ecosystems in comparison to with marine ecosystems is reflective of global patterns of protist diversity (Singer et al. 2021). The strong influence of salinity on microbial eukaryotic composition in the coastal communities highlights the influence of the salinity barrier on microbial eukaryotic diversity for High Arctic environments of the Last Ice Area, as identified across global marine environments (Logares et al. 2009). These distinct coastal communities may therefore be impacted by continued freshening from thawing terrestrial landscapes (Vincent 2010). Moreover, the greater similarity between the freshwater and terrestrial communities than with marine ecosystems suggests microbial eukaryotes are transported in hydrological networks between freshwater and terrestrial environments on Ward Hunt Island, and in the High Arctic (Harding et al. 2011, Comte et al. 2018). The high abundance of terrestrially sourced ciliates, Cercozoa, and Rozellomycota fungi in the microplankton suggests freshwater communities are highly influenced by input from terrestrial environments. Warming temperatures are increasing permafrost thaw and precipitation as rainfall in the High Arctic (Bintanja and Andry 2017, Edwards et al. 2020, IPCC 2023). Therefore, movement of microbial eukaryotic organisms between terrestrial and freshwater ecosystems may also increase facilitated by more hydrologically active river, lake and wetland catchments (Vincent 2010, Bégin et al. 2021). In turn, this could lead to changes in community composition which may alter biogeochemical cycles or food webs in oligotrophic lakes.

Only 3% of microbial eukaryotic taxa were identified in all sampling locations, suggesting most protists have specific habitat conditions, and few protists have cosmopolitan distribution (Petz et al. 2007, Bass et al. 2007). Cosmopolitan taxa were mostly dinoflagellates, ciliate, and Cercozoa, taxonomic lineages which are often capable of cyst formation, permitting broader dispersal patterns across the Arctic region (Harding et al. 2011, Jungblut et al. 2012) and even from pole to pole (Montresor et al. 2003). Ciliates were highly abundant in all communities and appear to play important roles in basal trophic interactions in High Arctic ecosystems due to their ability to predate smaller prokaryotic and eukaryotic organisms (Adl et al. 2019). However, it is possible their abundance in environmental communities may have been overestimated due to their higher copy number of ribosomal genes (Medinger et al. 2010).

Distinct and heterogenous populations of microbial eukaryotes were identified within terrestrial ecosystems and between freshwater ecosystems in the vicinity of Ward Hunt Island. This study suggests that microbial eukaryotic communities are as abundant and heterogeneous in Arctic aquatic and terrestrial ecosystems as described more thoroughly in lower latitude ecosystems (Oliverio et al. 2020, Sieber et al. 2020, Singer et al. 2021). Moreover, terrestrial ecosystems and benthic microbial mats appear to be sites of high heterogeneity in the Arctic. This is likely due to more distinct spatial patterns of environment conditions existing between these environments than in aquatic environments, which are mixed (Pernica et al. 2013, Sieber et al. 2020). The functional contribution of protists to freshwater microbial mats with distinct temperature, pH, TN, and TP conditions suggests that ecological function is also modulated by biotic factors, with the microbial eukaryotic community contributing specific functions based on trophic interactions and selection within the community, while taxonomic diversity may be more strongly influenced by local scale variations in abiotic and biotic factors across the Arctic. Future transcriptome-based characterization of the microbial communities would allow better identification of metabolically active or transcribed genes, demonstrated to better represent functional activity of the community (Campbell et al. 2011, Zhang et al. 2014).

## Conclusion

Utilizing full length 18S rRNA gene metabarcoding, we demonstrated that protists could have diverse trophic function and interactions in High Arctic terrestrial, freshwater, and coastal marine ecosystems proximal to the LIA. For the first time, this study extends biogeographical trends identified in microbial eukaryotes from lower latitude climates to the High Arctic. While terrestrially systems overall did not have the richest communities as hypothesized, microbial eukaryotic richness was highest in areas of higher soil moisture, suggesting Arctic microbial eukaryotic communities will be modified by increasing permafrost thaw and rainfall. Furthermore, we evaluated the connectivity of microbial eukaryotes between terrestrial and freshwater systems, which may change with ongoing climate warming due to more active hydrological catchments. These findings highlight important concerns for potential homogenization of microbial eukaryotic communities strongly influenced by current abiotic conditions. This underscores the importance of integrating microbial eukaryotic diversity into the monitoring of rapidly changing Arctic ecosystems—particularly within this “Decade of Action for Cryospheric Sciences”.

## Supplementary Material

fiag096_Supplemental_File

## Data Availability

The Oxford Nanopore Sequencing data generated in this study are available in the Sequence Read Archive with the accession number PRJNA1267098.
