# Supplementary material for: Diversity and connectivity of microbial eukaryotes in High Arctic terrestrial and aquatic ecosystems
Source: FEMS Microbiol Ecol. 2026 Oct 30;102(11):fiag096. doi: 10.1093/femsec/fiag096 (PMC13723083; doi:10.1093/femsec/fiag096)
Supplement: Supplementary file 1 [file fiag096_supplemental_file.docx]

**Supplementary Material**

**Diversity and connectivity of microbial eukaryotes in High Arctic terrestrial and aquatic ecosystems**

Hooper, P.M., Vincent, W.F. Girard, C. Feil, E.J.^2^ Bass, D. and Jungblut A.D.

# Supplementary Tables

**Supplementary Table 1.** Sample metadata. Sampling Sites: AP = Antoniades Pond, LA Inflow = Lake A Stream Inflow, QL = Quttinirpaaq Lagoon, WH = Ward Hunt Lake, WHIS = Ward Hunt Ice Shelf, WT = Water Track. Sample variables: Con = Specific conductivity, Diss. Oxygen Sat. = Dissolved oxygen saturation, GWC = Gravitational Water Content, Chl ɑ = Chlorophyll ɑ. Fmol refers to the final concentration of the sample prior to sequencing. Eukaryotic read depth is after removal of bacteria and archaea assigned reads.

| **Sample** | **Sample Group** | **Site** | **Sample Type** | **Ecosystem** | **Latitude** | **Longitude** | **Temperature (°C)** | **pH** | **Con (μS -1)** | **Diss. Oxygen Sat. (%)** | **GWC (%)** | **Chl ɑ µg/l** | **Vol (mL)** | **Fmol** | **Eukaryotic Read Depth** |
| --- | --- | --- | --- | --- | --- | --- | --- | --- | --- | --- | --- | --- | --- | --- | --- |
| AP_M1 | AP Microbial Mat | AP | Microbial Mat | Freshwater | 82.9839 | -75.4009 | 9.6 | 8.4 | 100 | - | - | - | - | 20.7 | 4661 |
| AP_M2 | AP Microbial Mat | AP | Microbial Mat | Freshwater | 82.984 | -75.4008 | 8.1 | 8.12 | 400 | - | - | - | - | 146.7 | 44045 |
| AP_M3 | AP Microbial Mat | AP | Microbial Mat | Freshwater | 82.984 | -75.4008 | 7 | 8.21 | 100 | - | - | - | - | 17.6 | 3630 |
| AP_M4 | AP Microbial Mat | AP | Microbial Mat | Freshwater | 82.9841 | -75.4003 | 8.2 | 8.32 | 110 | - | - | - | - | 22.1 | 5200 |
| AP_M5 | AP Microbial Mat | AP | Microbial Mat | Freshwater | 82.9843 | -75.4003 | 6.9 | 8.07 | 110 | - | - | - | - | 13.7 | 2944 |
| AP_M6 | AP Microbial Mat | AP | Microbial Mat | Freshwater | 82.9846 | -75.4009 | 6.9 | 8.14 | 110 | - | - | - | - | 92.8 | 16230 |
| AP_M7 | AP Microbial Mat | AP | Microbial Mat | Freshwater | 82.9846 | -75.401 | 6.9 | 8.16 | 110 | - | - | - | - | 38.4 | 14864 |
| AP_W1 | AP Water | AP | Microplankton | Freshwater | 82.9839 | -75.4009 | 9.3 | 8.22 | 110 | - | - | - | 240 | 190.0 | 67571 |
| AP_W2 | AP Water | AP | Microplankton | Freshwater | 82.9841 | -75.4003 | 6.9 | 8.01 | 110 | - | - | - | 240 | 200.0 | 57512 |
| AP_W3 | AP Water | AP | Microplankton | Freshwater | 82.9846 | -75.401 | 6.1 | 7.41 | 110 | - | - | - | 240 | 200.0 | 81098 |
| LA_M1 | Stream Mat | LA_Inflow | Microplankton | Freshwater | 82.9821 | -75.4406 | 3.3 | 7.3 | 250 | - | - | - | - | 45.7 | 14064 |
| LA_M2 | Stream Mat | LA_Inflow | Microplankton | Freshwater | 82.9821 | -75.441 | 4.8 | 7.46 | 240 | - | - | - | - | 39.1 | 8425 |
| LA_M3 | Stream Mat | LA_Inflow | Microplankton | Freshwater | 82.9821 | -75.4411 | 4.9 | 7.46 | 240 | - | - | - | - | 52.0 | 23186 |
| QL_M1 | QL Microbial Mat | QL | Microbial Mat | Coastal | 83.0928 | -74.1186 | 2.1 | 8.6 | 4400 | - | - | - | - | 127.0 | 44503 |
| QL_M2 | QL Microbial Mat | QL | Microbial Mat | Coastal | 83.0963 | -74.2959 | 2.1 | 8.54 | 2080 | - | - | - | - | 68.7 | 17801 |
| QL_M3 | QL Microbial Mat | QL | Microbial Mat | Coastal | 83.0966 | -74.2877 | 1.3 | 8.41 | 2530 | - | - | - | - | 86.1 | 23142 |
| QL_M4 | QL Microbial Mat | QL | Microbial Mat | Coastal | 83.0967 | -74.2793 | 2 | 8.35 | 2580 | - | - | - | - | 23.8 | 7009 |
| QL_M5 | QL Microbial Mat | QL | Microbial Mat | Coastal | 83.0969 | -74.2729 | 0.81 | 8.28 | 2340 | - | - | - | - | 41.3 | 16740 |
| QL_M6 | QL Microbial Mat | QL | Microbial Mat | Coastal | 83.0973 | -74.2534 | 1.6 | 8.11 | 4210 | - | - | - | - | 37.2 | 9728 |
| QL_M7 | QL Microbial Mat | QL | Microbial Mat | Coastal | 83.0974 | -74.2479 | 2 | 8.28 | 4270 | - | - | - | - | 38.0 | 10422 |
| QL_M8 | QL Microbial Mat | QL | Microbial Mat | Coastal | 83.0972 | -74.2251 | 1.5 | 8.13 | 4270 | - | - | - | - | 73.4 | 18573 |
| QL_M9 | QL Microbial Mat | QL | Microbial Mat | Coastal | 83.0964 | -74.1963 | 1.9 | 8.27 | 4530 | - | - | - | - | 103.4 | 24738 |
| QL_M10 | QL Microbial Mat | QL | Microbial Mat | Coastal | 83.0952 | -74.17 | 2.5 | 8.25 | 4380 | - | - | - | - | 47.6 | 14751 |
| QL_W1 | QL Water | QL | Microplankton | Coastal | 83.0973 | -74.2534 | 1.3 | 8.47 | 2840 | - | - | - | 1500 | 187.3 | 71548 |
| QL_W2 | QL Water | QL | Microplankton | Coastal | 83.0972 | -74.2251 | 0.9 | 8.25 | 3200 | - | - | - | 1500 | 200.0 | 18001 |
| QL_W3 | QL Water | QL | Microplankton | Coastal | 83.0952 | -74.17 | 2.9 | 7.11 | 4550 | - | - | - | 1500 | 200.0 | 78428 |
| WH_M1 | WHL Moat Mat | WHL | Microbial Mat | Freshwater | 83.0883 | -74.1624 | 4.5 | 8.5 | 180 | - | - | - | - | 11.9 | 3158 |
| WH_M2 | WHL Moat Mat | WHL | Microbial Mat | Freshwater | 83.0885 | -74.1604 | 3.4 | 8.5 | 110 | - | - | - | - | 15.1 | 2427 |
| WH_M3 | WHL Moat Mat | WHL | Microbial Mat | Freshwater | 83.0883 | -74.165 | 4.6 | 8.54 | 120 | - | - | - | - | 10.7 | 3131 |
| WH_M4 | WHL Moat Mat | WHL | Microbial Mat | Freshwater | 83.088 | -74.1685 | 3.9 | 8.54 | 110 | - | - | - | - | 11.9 | 7925 |
| WH_M6 | WHL Moat Mat | WHL | Microbial Mat | Freshwater | 83.0858 | -74.1753 | 4.8 | 8.5 | 120 | - | - | - | - | 31.9 | 7819 |
| WH_M7 | WHL Moat Mat | WHL | Microbial Mat | Freshwater | 83.0851 | -74.1682 | 4 | 8.43 | 130 | - | - | - | - | 71.2 | 4757 |
| WH_W1 | WHL Moat Water | WHL | Microplankton | Freshwater | 83.0883 | -74.1654 | 2.9 | 8.65 | 120 | - | - | - | 1800 | 200.0 | 66450 |
| WH_W2 | WHL Moat Water | WHL | Microplankton | Freshwater | 83.0884 | -74.1547 | 2.9 | 8.42 | 110 | - | - | - | 1500 | 92.0 | 36405 |
| WH_W3 | WHL Moat Water | WHL | Microplankton | Freshwater | 83.0883 | -74.1508 | 2.5 | 8.5 | 110 | - | - | - | 1500 | 30.6 | 9975 |
| WH_W4 | WHL 2m Water | WHL | Microplankton | Freshwater | 83.0871 | -74.1456 | 2.99 | - | 171 | 104.46 | - | 0.75 | 548 | 200.0 | 25625 |
| WH_W5 | WHL 2m Water | WHL | Microplankton | Freshwater | 83.0871 | -74.1456 | 2.99 | - | 171 | 104.46 | - | 0.75 | 633 | 74.8 | 10440 |
| WH_W6 | WHL 2m Water | WHL | Microplankton | Freshwater | 83.0871 | -74.1456 | 2.99 | - | 171 | 104.46 | - | 0.75 | 776 | 200.0 | 40566 |
| WH_W7 | WHL 4m Water | WHL | Microplankton | Freshwater | 83.0871 | -74.1456 | 3.91 | - | 270 | 118.85 | - | 0.60 | 946 | 194.6 | 84650 |
| WH_W8 | WHL 4m Water | WHL | Microplankton | Freshwater | 83.0871 | -74.1456 | 3.91 | - | 270 | 118.85 | - | 0.60 | 906 | 200.0 | 74405 |
| WH_W9 | WHL 4m Water | WHL | Microplankton | Freshwater | 83.0871 | -74.1456 | 3.91 | - | 270 | 118.85 | - | 0.60 | 776 | 125.8 | 32122 |
| WH_W10 | WHL 8m Water | WHL | Microplankton | Freshwater | 83.0871 | -74.1456 | 3.65 | - | 312 | 81.45 | - | 3.99 | 861 | 58.8 | 26160 |
| WH_W11 | WHL 8m Water | WHL | Microplankton | Freshwater | 83.0871 | -74.1456 | 3.65 | - | 312 | 81.45 | - | 3.99 | 956 | 63.5 | 22375 |
| WH_W12 | WHL 8m Water | WHL | Microplankton | Freshwater | 83.0871 | -74.1456 | 3.65 | - | 312 | 81.45 | - | 3.99 | 1006 | 63.5 | 24620 |
| WHIS2_M1 | WHIS2 Microbial Mat | WHIS | Microbial Mat | Freshwater | 83.0843 | -74.3727 | 1.7 | 9.56 | 800 | - | - | - | - | 52.1 | 18506 |
| WHIS2_M2 | WHIS2 Microbial Mat | WHIS | Microbial Mat | Freshwater | 83.0843 | -74.3727 | 1.1 | 9.5 | 710 | - | - | - | - | 49.3 | 17246 |
| WHIS2_M3 | WHIS2 Microbial Mat | WHIS | Microbial Mat | Freshwater | 83.0843 | -74.3727 | 0.9 | 9.92 | 870 | - | - | - | - | 35.5 | 11376 |
| WHIS3_M1 | WHIS3 Microbial Mat | WHIS | Microbial Mat | Freshwater | 83.0842 | -74.3723 | 1.6 | 9.71 | 1920 | - | - | - | - | 66.0 | 19869 |
| WHIS3_M2 | WHIS3 Microbial Mat | WHIS | Microbial Mat | Freshwater | 83.0842 | -74.3723 | 1.4 | 9.55 | 2730 | - | - | - | - | 10.4 | 21579 |
| WHIS3_M3 | WHIS3 Microbial Mat | WHIS | Microbial Mat | Freshwater | 83.0842 | -74.3723 | 2.4 | 9.51 | 2270 | - | - | - | - | 60.5 | 15117 |
| WT1_C1 | WT1 Soil Crust | Walker_Hill | Soil Crust | Terrestrial | 83.0869 | -74.1727 | - | 7.2 | 473 | - | 96.58 | - | - | 14.9 | 3985 |
| WT1_C2 | WT1 Soil Crust | Walker_Hill | Soil Crust | Terrestrial | 83.0869 | -74.1763 | - | 7.76 | 255 | - | 53.96 | - | - | 15.6 | 2623 |
| WT1_C3 | WT1 Soil Crust | Walker_Hill | Soil Crust | Terrestrial | 83.087 | -74.1784 | - | 7.65 | 429 | - | 63.81 | - | - | 44.6 | 13048 |
| WT1_C4 | WT1 Soil Crust | Walker_Hill | Soil Crust | Terrestrial | 83.087 | -74.1784 | - | 7.56 | 602 | - | 105.83 | - | - | 42.9 | 13891 |
| WT1_C5 | WT1 Soil Crust | Walker_Hill | Soil Crust | Terrestrial | 83.087 | -74.1787 | - | 7.63 | 407 | - | 79.16 | - | - | 14.3 | 3387 |
| WT1_M1 | WT1 Microbial Mat | Walker_Hill | Microbial Mat | Terrestrial | 83.0869 | -74.176 | 4.8 | 7.52 | 170 | - | - | - | - | 35.7 | 10616 |
| WT1_M2 | WT1 Microbial Mat | Walker_Hill | Microbial Mat | Terrestrial | 83.0869 | -74.1761 | 4.1 | 7.32 | 150 | - | - | - | - | 185.2 | 61006 |
| WT1_M3 | WT1 Microbial Mat | Walker_Hill | Microbial Mat | Terrestrial | 83.0869 | -74.1763 | 3.9 | 7.36 | 150 | - | - | - | - | 93.8 | 31427 |
| WT1_M4 | WT1 Microbial Mat | Walker_Hill | Microbial Mat | Terrestrial | 83.0871 | -74.1783 | 6 | 7.4 | 170 | - | - | - | - | 148.8 | 48514 |
| WT1_M5 | WT1 Microbial Mat | Walker_Hill | Microbial Mat | Terrestrial | 83.0871 | -74.1785 | 5.1 | 7.34 | 170 | - | - | - | - | 99.6 | 31879 |
| WT1_S3 | WT1 Soil | Walker_Hill | Soil | Terrestrial | 83.087 | -74.1784 | - | 8.17 | 120 | - | 22.13 | - | - | 31.2 | 2490 |
| WT1_W1 | WT1 Microplankton | Walker_Hill | Microplankton | Freshwater | 83.0869 | -74.176 | 4.8 | 7.52 | 170 | - | - | - | 200 | 47.0 | 1840 |
| WT1_W3 | WT1 Microplankton | Walker_Hill | Microplankton | Freshwater | 83.0871 | -74.1785 | 5.1 | 7.34 | 170 | - | - | - | 420 | 13.2 | 3062 |
| WT2_C1 | WT2 Soil Crust | Walker_Hill | Soil Crust | Terrestrial | 83.0872 | -74.1751 | - | 7.77 | 359 | - | 52.73 | - | - | 37.6 | 15964 |
| WT2_C2 | WT2 Soil Crust | Walker_Hill | Soil Crust | Terrestrial | 83.0872 | -74.1751 | - | 7.67 | 384 | - | 45.41 | - | - | 48.7 | 13161 |
| WT2_C3 | WT2 Soil Crust | Walker_Hill | Soil Crust | Terrestrial | 83.0872 | -74.1751 | - | 7.78 | 268 | - | 40.37 | - | - | 57.6 | 26589 |
| WT2_C4 | WT2 Soil Crust | Walker_Hill | Soil Crust | Terrestrial | 83.0872 | -74.1752 | - | 7.77 | 330 | - | 46.11 | - | - | 27.6 | 8937 |
| WT2_C5 | WT2 Soil Crust | Walker_Hill | Soil Crust | Terrestrial | 83.0873 | -74.1753 | - | 7.89 | 229 | - | 32.02 | - | - | 30.0 | 7634 |
| WT2_M1 | WT2 Microbial Mat | Walker_Hill | Microbial Mat | Terrestrial | 83.0873 | -74.1752 | 7.5 | 7.13 | 220 | - | - | - | - | 81.1 | 23564 |
| WT2_M2 | WT2 Microbial Mat | Walker_Hill | Microbial Mat | Terrestrial | 83.0873 | -74.1751 | 7.5 | 7.19 | 220 | - | - | - | - | 133.2 | 40403 |
| WT2_M3 | WT2 Microbial Mat | Walker_Hill | Microbial Mat | Terrestrial | 83.0873 | -74.175 | 7.5 | 7.19 | 210 | - | - | - | - | 47.5 | 34779 |
| WT2_M4 | WT2 Microbial Mat | Walker_Hill | Microbial Mat | Terrestrial | 83.0873 | -74.1748 | 7.4 | 7.27 | 210 | - | - | - | - | 56.5 | 19482 |
| WT2_M5 | WT2 Microbial Mat | Walker_Hill | Microbial Mat | Terrestrial | 83.0873 | -74.1747 | 6.6 | 7.31 | 200 | - | - | - | - | 105.1 | 32881 |
| WT2_S1 | WT2 Soil | Walker_Hill | Soil | Terrestrial | 83.0872 | -74.1751 | - | 8.17 | 174 | - | 27.15 | - | - | 42.7 | 2420 |
| WT2_W1 | WT2 Microplankton | Walker_Hill | Microplankton | Freshwater | 83.0873 | -74.1752 | 7.5 | 7.13 | 220 | - | - | - | 180 | 22.0 | 5587 |
| WT3_C1 | WT3_Soil_Crust | Walker_Hill | Soil Crust | Terrestrial | 83.0875 | -74.1743 | - | 7.45 | 760 | - | 154.03 | - | - | 28.7 | 5927 |
| WT3_C2 | WT3_Soil_Crust | Walker_Hill | Soil Crust | Terrestrial | 83.0875 | -74.1744 | - | 7.46 | 700 | - | 142.51 | - | - | 22.5 | 5814 |
| WT3_C3 | WT3_Soil_Crust | Walker_Hill | Soil Crust | Terrestrial | 83.0875 | -74.1744 | - | 7.43 | 726 | - | 132.42 | - | - | 29.7 | 8107 |
| WT3_C4 | WT3_Soil_Crust | Walker_Hill | Soil Crust | Terrestrial | 83.0875 | -74.1744 | - | 7.38 | 774 | - | 180.4 | - | - | 32.2 | 7915 |
| WT3_C5 | WT3_Soil_Crust | Walker_Hill | Soil Crust | Terrestrial | 83.0875 | -74.1745 | - | 7.48 | 631 | - | 159.49 | - | - | 20.7 | 6686 |
| WT3_M1 | WT3 Microbial Mat | Walker_Hill | Microbial Mat | Terrestrial | 83.0875 | -74.1744 | - | - | - | - | - | - | - | 57.3 | 18163 |
| WT3_M2 | WT3 Microbial Mat | Walker_Hill | Microbial Mat | Terrestrial | 83.0875 | -74.1744 | - | - | - | - | - | - | - | 101.7 | 28250 |
| WT3_M3 | WT3 Microbial Mat | Walker_Hill | Microbial Mat | Terrestrial | 83.0875 | -74.1743 | - | - | - | - | - | - | - | 87.3 | 19104 |
| WT3_M4 | WT3 Microbial Mat | Walker_Hill | Microbial Mat | Terrestrial | 83.0875 | -74.1743 | - | - | - | - | - | - | - | 104.1 | 22049 |
| WT3_M5 | WT3 Microbial Mat | Walker_Hill | Microbial Mat | Terrestrial | 83.0875 | -74.1744 | - | - | - | - | - | - | - | 42.5 | 15693 |
| WT3_S2 | WT3 Soil | Walker_Hill | Soil | Terrestrial | 83.0875 | -74.1744 | - | 8.35 | 112 | - | 17.48 | - | - | 21.7 | 722 |
| WT3_S4 | WT3 Soil | Walker_Hill | Soil | Terrestrial | 83.0875 | -74.1744 | - | 8.27 | 130 | - | 19.38 | - | - | 22.0 | 1241 |
| WT3_S5 | WT3 Soil | Walker_Hill | Soil | Terrestrial | 83.0875 | -74.1745 | - | 8.3 | 134 | - | 17.74 | - | - | 31.4 | 8333 |

**Supplementary Table 2.** Nutrient conditions from Ward Hunt Lake (WHL), Antoniades Pond (AP) and Quttinirpaaq Lagoon (QL).

| **Date** | **Site** | **ID** | **Type** | **TN (μg N/L)** | **TP (μg P/L)** | **DIC (mg/L)** | **DOC (mg/L)** | **TDN (mg/L)** | **Fluoride (mg/L)** | **Chloride (mg/L)** | **Nitrite (mg/L)** | **Sulfate (mg/L)** | **Bromide (mg/L)** | **Nitrate (mg/L)** | **Phosphate (mg/L)** | **Ca (μg/L)** | **Fe (μg/L)** | **K (μg/L)** | **Mg (μg/L)** | **Mn (μg/L)** | **Na (μg/L)** |
| --- | --- | --- | --- | --- | --- | --- | --- | --- | --- | --- | --- | --- | --- | --- | --- | --- | --- | --- | --- | --- | --- |
| 22/07/2022 | AP | AP_25cm_R1 | Freshwater | 342 | 20.4 | 12.36 | 2.55 | 0.33 | 0.011 | 8.66 | <0.005 | 2.16 | <0.002 | 0.025 | <0.03 | 11.8 | <LQ | 0.83 | 3.35 | <LQ | 4.2 |
| 22/07/2022 | AP | AP_25cm_R1 | Freshwater | 356 | 27.3 | 12.64 | 2.65 | 0.33 | 0.011 | 8.96 | <0.005 | 2.19 | <0.002 | 0.025 | <0.03 | 12.3 | 83 | 0.91 | 3.41 | <LQ | 4.4 |
| 22/07/2022 | AP | AP_Surface_R1 | Freshwater | 372 | 27.7 | 11.35 | 2.35 | 0.32 | 0.018 | 8.72 | <0.005 | 1.99 | <0.002 | 0.025 | <0.03 | 12.6 | 95 | 0.78 | 3.59 | <LQ | 4.2 |
| 22/07/2022 | AP | AP_Surface_R1 | Freshwater | 342 | 19.0 | 11.35 | 2.51 | 0.31 | 0.013 | 8.71 | <0.005 | 1.98 | <0.002 | 0.026 | <0.03 | 13.0 | <LQ | 0.77 | 3.50 | <LQ | 4.1 |
| 20/07/2022 | QL | QL_R1 | Coastal | NA | NA | NA | NA | NA | 0.066 | 962 | 0.048 | 153 | 3.26 | 0.047 | < 0.1 | 0.0064 | <LQ | 22.3 | 0.06 | <LQ | 1.014 |
| 20/07/2022 | QL | QL_R2 | Coastal | NA | NA | NA | NA | NA | 1.10 | 1446 | 0.57 | 226 | 4.86 | 5.06 | < 4 | 0.0175 | <LQ | 36.5 | 0.10 | <LQ | 0.794 |
| 23/07/2022 | WHL | WHL_25cm_R1 | Freshwater | 106 | 13.8 | 16.91 | 0.60 | 0.06 | 0.013 | 3.81 | <0.005 | 2.87 | 0.017 | <0.003 | <0.03 | 18.4 | <LQ | 0.66 | 3.43 | <LQ | 2.9 |
| 23/07/2022 | WHL | WHL_25cm_R2 | Freshwater | 123 | 12.3 | 18.25 | 2.97 | 0.09 | 0.035 | 3.79 | <0.005 | 2.87 | 0.016 | 0.019 | <0.03 | 17.8 | <LQ | 0.55 | 3.39 | <LQ | 2.8 |
| 23/07/2022 | WHL | WHL_Surface_R1 | Freshwater | 106 | 11.4 | 17.43 | 0.64 | 0.08 | 0.018 | 4.00 | <0.005 | 3.01 | 0.014 | 0.480 | <0.03 | 19.3 | <LQ | 0.68 | 3.55 | <LQ | 2.9 |
| 23/07/2022 | WHL | WHL_Surface_R2 | Freshwater | 115 | 13.6 | 17.46 | 0.60 | 0.07 | 0.021 | 4.10 | <0.005 | 3.02 | 0.015 | 0.044 | <0.03 | 18.6 | <LQ | 0.56 | 3.58 | <LQ | 2.9 |
| 18/07/2022 | WHL | Under Ice_R1 | Freshwater | 92.2 | 13.7 | 6.83 | 0.32 | 0.05 | 0.02 | 1.18 | <0.005 | 0.97 | 0.01 | 0.03 | <0.03 |  |  |  |  |  |  |
| 18/07/2022 | WHL | Under Ice_R2 | Freshwater | 127.2 | 17.7 | 3.94 | 0.17 | 0.06 | 0.03 | 2.96 | <0.005 | 1.45 | 0.01 | 0.02 | 0.04 | 6.5 | <LQ | 0.49 | 1.15 | <LQ | <LQ |
| 18/07/2022 | WHL | Under Ice_R3 | Freshwater | NA | NA | NA | NA | NA | 0.03 | 3.03 | <0.005 | 2.08 | 0.01 | 0.03 | <0.03 | 3.3 | <LQ | 0.51 | 0.48 | <LQ | <LQ |
| 18/07/2022 | WHL | 2m_R1 | Freshwater | 137.9 | 16.1 | 9.47 | 0.40 | 0.06 | 0.03 | 1.01 | <0.005 | 0.79 | 0.01 | 0.03 | 0.04 | 8.2 | <LQ | 0.51 | 1.43 | <LQ | <LQ |
| 18/07/2022 | WHL | 2m_R2 | Freshwater | 108.4 | 18.4 | 14.15 | 0.65 | 0.07 | 0.02 | 0.50 | <0.005 | 0.34 | <0.002 | 0.03 | 0.06 | 10.1 | <LQ | 0.51 | 1.83 | <LQ | 2.1 |
| 18/07/2022 | WHL | 2m_R3 | Freshwater | NA | NA | NA | NA | NA | 0.03 | 3.09 | <0.005 | 1.90 | 0.01 | 0.03 | <0.03 | 15.2 | <LQ | 0.72 | 2.72 | <LQ | 2.5 |
| 18/07/2022 | WHL | 4m_R1 | Freshwater | 127.2 | 14.0 | 28.21 | 3.03 | 0.11 | 0.02 | 7.22 | <0.005 | 4.85 | 0.03 | 0.01 | <0.03 | 30.6 | <LQ | 0.74 | 5.61 | <LQ | 5.1 |
| 18/07/2022 | WHL | 4m_R2 | Freshwater | 128.5 | 14.2 | 27.98 | 0.81 | 0.07 | 0.03 | 8.16 | <0.005 | 5.48 | 0.04 | 0.02 | <0.03 | 28.1 | <LQ | 0.70 | 5.20 | <LQ | 4.6 |
| 18/07/2022 | WHL | 4m_R3 | Freshwater | NA | NA | NA | NA | NA | 0.04 | 8.22 | <0.005 | 5.34 | 0.04 | 0.02 | <0.03 | 31.4 | <LQ | 0.73 | 5.72 | <LQ | 5.1 |
| 18/07/2022 | WHL | 8m_R1 | Freshwater | 155.4 | 26.4 | 33.40 | 0.87 | 0.09 | 0.05 | 9.07 | <0.005 | 6.07 | 0.04 | 0.02 | <0.03 | 36.7 | <LQ | 0.79 | 6.51 | <LQ | 5.7 |
| 18/07/2022 | WHL | 8m_R2 | Freshwater | 156.7 | 28.6 | 33.17 | 0.87 | 0.09 | 0.05 | 9.23 | <0.005 | 6.16 | 0.04 | 0.02 | <0.03 | 37.1 | <LQ | 0.81 | 6.69 | <LQ | 5.8 |
| 18/07/2022 | WHL | 8m_R3 | Freshwater | NA | NA | NA | NA | NA | 0.04 | 8.69 | <0.005 | 6.01 | 0.04 | 0.02 | <0.03 | NA | NA | NA | NA | NA | NA |
| 18/07/2022 | WHL | 9m_R1 | Freshwater | 235.7 | 14.9 | 34.81 | 0.85 | 0.16 | 0.04 | 8.61 | <0.005 | 4.88 | 0.05 | 0.02 | <0.03 | 39.7 | 179 | 0.86 | 6.59 | 362.0 | 5.7 |
| 18/07/2022 | WHL | 9m_R2 | Freshwater | 183.9 | 13.0 | 34.46 | 0.84 | 0.12 | 0.04 | 8.37 | <0.005 | 5.47 | 0.05 | 0.02 | <0.03 | 38.7 | <LQ | 0.78 | 6.51 | 175.2 | 5.4 |
| 18/07/2022 | WHL | 9m_R3 | Freshwater | NA | NA | NA | NA | NA | 0.06 | 8.89 | <0.005 | 5.56 | 0.05 | 0.03 | <0.03 | 39.2 | <LQ | 0.84 | 6.57 | 186.1 | 5.7 |

**Supplementary Table 3.** Functional annotation of protist taxa assigned by the PR^2^ database (V5.0.0).

| **TaxID** | **Supergroup** | **Division** | **Subdivision** | **Class** | **Order** | **Family** | **Genus** | **Species** | **Trophic Function** |
| --- | --- | --- | --- | --- | --- | --- | --- | --- | --- |
| 2854 | Amoebozoa | Discosea | Discosea_X | Centramoebia | Acanthopodida | Acanthamoebidae | *Acanthamoeba* | *Acanthamoeba_sp.* | Bacterivore |
| 2881 | Amoebozoa | Discosea | Discosea_X | Centramoebia | Himatismenida | Himatismenida_X | *Squamamoeba* | *Squamamoeba_japonica* | Bacterivore |
| 2884 | Amoebozoa | Discosea | Discosea_X | Centramoebia | Himatismenida | Parvamoebidae | *Parvamoeba* | *Parvamoeba_sp.* | Bacterivore |
| 2823 | Amoebozoa | Discosea | Discosea_X | Flabellinia | Dermamoebida | Mayorellidae | *Mayorella* | *Mayorella_gemmifera* | Bacterivore |
| 74786 | Amoebozoa | Discosea | Discosea_X | Flabellinia | Flabellinia_X | LKM74-lineage | *Mycamoeba* | *Mycamoeba_gemmipara* | Bacterivore |
| 74781 | Amoebozoa | Discosea | Discosea_X | Flabellinia | Vannellida | Vannellidae | *Ripella* | *Ripella_decalvata* | Bacterivore |
| 2803 | Amoebozoa | Discosea | Discosea_X | Flabellinia | Vannellida | Vannellidae | *Ripella* | *Ripella_sp.* | Bacterivore |
| 74783 | Amoebozoa | Discosea | Discosea_X | Flabellinia | Vannellida | Vannellidae | *Ripella* | *Ripella_tribonemae* | Bacterivore |
| 2809 | Amoebozoa | Discosea | Discosea_X | Flabellinia | Vannellida | Vannellidae | *Vannella* | *Vannella_contorta* | Bacterivore |
| 2819 | Amoebozoa | Discosea | Discosea_X | Flabellinia | Vannellida | Vannellidae | *Vannella* | *Vannella_sp.* | Bacterivore |
| 2423 | Amoebozoa | Evosea | Evosea_X | Archamoebea | Archamoebea_X | Entamoebidae | *Entamoeba* | *Entamoeba_equi* | Bacterivore |
| 2731 | Amoebozoa | Evosea | Evosea_X | Variosea | Flamellidae | ATCC50593-lineage | *ATCC50593-lineage_X* | *ATCC50593-lineage_X_sp.* | Bacterivore |
| 74803 | Amoebozoa | Evosea | Evosea_X | Variosea | Flamellidae | Flamella-lineage | *Flamella* | *Flamella_beringiana* | Bacterivore |
| 2738 | Amoebozoa | Evosea | Evosea_X | Variosea | Flamellidae | Flamella-lineage | *Flamella-lineage_X* | *Flamella-lineage_X_sp.* | Bacterivore |
| 2764 | Amoebozoa | Evosea | Evosea_X | Variosea | Fractovitellida | Schizoplasmodiidae | *Ceratiomyxella* | *Ceratiomyxella_tahitiensis* | Bacterivore |
| 2766 | Amoebozoa | Evosea | Evosea_X | Variosea | Fractovitellida | Schizoplasmodiidae | *Schizoplasmodium* | *Schizoplasmodium_cavostelioides* | Bacterivore |
| 2767 | Amoebozoa | Evosea | Evosea_X | Variosea | Fractovitellida | Soliformoviidae | *Grellamoeba* | *Grellamoeba_robusta* | Bacterivore |
| 74811 | Amoebozoa | Evosea | Evosea_X | Variosea | Variosea_X | Ischnamoebidae | *Darbyshirella* | *Darbyshirella_terrestris* | Bacterivore |
| 2770 | Amoebozoa | Evosea | Evosea_X | Variosea | Variosea_X | Variosea_XX | *Variosea_XXX* | *Variosea_XXX_sp.* | Bacterivore |
| 2773 | Amoebozoa | Evosea | Evosea_X | Variosea | Variosea_X | WIM1-lineage | *WIM1-lineage_X* | *WIM1-lineage_X_sp.* | Bacterivore |
| 2931 | Amoebozoa | Tubulinea | Tubulinea_X | Echinamoebida | Echinamoebida_X | Echinamoebidae | *Echinamoeba* | *Echinamoeba_exundans* | Bacterivore |
| 2932 | Amoebozoa | Tubulinea | Tubulinea_X | Echinamoebida | Echinamoebida_X | Echinamoebidae | *Echinamoeba* | *Echinamoeba_sp.* | Bacterivore |
| 2893 | Amoebozoa | Tubulinea | Tubulinea_X | Elardia | Arcellinida | Phryganellina | *Cryptodifflugia* | *Cryptodifflugia_operculata* | Bacterivore |
| 74815 | Amoebozoa | Tubulinea | Tubulinea_X | Elardia | Euamoebida | Hartmannellidae | *Copromyxa* | *Copromyxa_laresi* | Bacterivore |
| 2948 | Amoebozoa | Tubulinea | Tubulinea_X | Elardia | Euamoebida | Hartmannellidae | *Hartmannellidae_X* | *Hartmannellidae_X_sp.* | Bacterivore |
| 2951 | Amoebozoa | Tubulinea | Tubulinea_X | Elardia | Euamoebida | Hartmannellidae | *Saccamoeba* | *Saccamoeba_sp.* | Bacterivore |
| 2952 | Amoebozoa | Tubulinea | Tubulinea_X | Elardia | Euamoebida | LOS7N/I-lineage | *LOS7N/I-lineage_X* | *LOS7N/I-lineage_X_sp.* | Bacterivore |
| 2953 | Amoebozoa | Tubulinea | Tubulinea_X | Elardia | Euamoebida | LOS7N/I-lineage | *Ptolemeba* | *Ptolemeba_bulliensis* | Bacterivore |
| 2954 | Amoebozoa | Tubulinea | Tubulinea_X | Elardia | Euamoebida | LOS7N/I-lineage | *Ptolemeba* | *Ptolemeba_noxubium* | Bacterivore |
| 2968 | Amoebozoa | Tubulinea | Tubulinea_X | Elardia | Euamoebida | Nolandellidae | *Nolandellidae_X* | *Nolandellidae_X_sp.* | Bacterivore |
| 74819 | Amoebozoa | Tubulinea | Tubulinea_X | Elardia | Leptomyxida | Flabellulidae | *Flabellula* | *Flabellula_pomeranica* | Bacterivore |
| 2960 | Amoebozoa | Tubulinea | Tubulinea_X | Elardia | Leptomyxida | Flabellulidae | *Paraflabellula* | *Paraflabellula_sp.* | Bacterivore |
| 77341 | Archaeplastida | Chlorophyta | Chlorophyta_X | Chlorophyceae | Chaetopeltidales | Chaetopeltidaceae | *Planophila* | *Planophila_bipyrenoidosa* | Phototrophic |
| 4355 | Archaeplastida | Chlorophyta | Chlorophyta_X | Chlorophyceae | Chaetopeltidales | Chaetopeltidaceae | *Planophila* | *Planophila_laetevirens* | Phototrophic |
| 3998 | Archaeplastida | Chlorophyta | Chlorophyta_X | Chlorophyceae | Chaetopeltidales | Chaetopeltidaceae | *Planophila* | *Planophila_sp.* | Phototrophic |
| 3030 | Archaeplastida | Chlorophyta | Chlorophyta_X | Chlorophyceae | Chaetopeltidales | Chaetopeltidales_X | *Chaetopeltis* | *Chaetopeltis_orbicularis* | Phototrophic |
| 3031 | Archaeplastida | Chlorophyta | Chlorophyta_X | Chlorophyceae | Chaetopeltidales | Chaetopeltidales_X | *Floydiella* | *Floydiella_terrestris* | Phototrophic |
| 3032 | Archaeplastida | Chlorophyta | Chlorophyta_X | Chlorophyceae | Chaetopeltidales | Chaetopeltidales_X | *Hormotilopsis* | *Hormotilopsis_gelatinosa* | Phototrophic |
| 3088 | Archaeplastida | Chlorophyta | Chlorophyta_X | Chlorophyceae | Chlamydomonadales | Chlamydomonadales_X | *Actinochloris* | *Actinochloris_sp.* | Phototrophic |
| 3100 | Archaeplastida | Chlorophyta | Chlorophyta_X | Chlorophyceae | Chlamydomonadales | Chlamydomonadales_X | *Brachiomonas* | *Brachiomonas_sp.* | Phototrophic |
| 3105 | Archaeplastida | Chlorophyta | Chlorophyta_X | Chlorophyceae | Chlamydomonadales | Chlamydomonadales_X | *Carteria* | *Carteria_lunzensis* | Phototrophic |
| 3109 | Archaeplastida | Chlorophyta | Chlorophyta_X | Chlorophyceae | Chlamydomonadales | Chlamydomonadales_X | *Carteria* | *Carteria_sp.* | Phototrophic |
| 3115 | Archaeplastida | Chlorophyta | Chlorophyta_X | Chlorophyceae | Chlamydomonadales | Chlamydomonadales_X | *Chlamydocapsa* | *Chlamydocapsa_sp.* | Phototrophic |
| 3243 | Archaeplastida | Chlorophyta | Chlorophyta_X | Chlorophyceae | Chlamydomonadales | Chlamydomonadales_X | *Chlamydomonadales_XX* | *Chlamydomonadales_XX_sp.* | Phototrophic |
| 76071 | Archaeplastida | Chlorophyta | Chlorophyta_X | Chlorophyceae | Chlamydomonadales | Chlamydomonadales_X | *Chlamydomonas* | *Chlamydomonas_gloeophila* | Phototrophic |
| 3131 | Archaeplastida | Chlorophyta | Chlorophyta_X | Chlorophyceae | Chlamydomonadales | Chlamydomonadales_X | *Chlamydomonas* | *Chlamydomonas_hedleyi* | Phototrophic |
| 3132 | Archaeplastida | Chlorophyta | Chlorophyta_X | Chlorophyceae | Chlamydomonadales | Chlamydomonadales_X | *Chlamydomonas* | *Chlamydomonas_hydra* | Phototrophic |
| 3146 | Archaeplastida | Chlorophyta | Chlorophyta_X | Chlorophyceae | Chlamydomonadales | Chlamydomonadales_X | *Chlamydomonas* | *Chlamydomonas_noctigama* | Phototrophic |
| 3150 | Archaeplastida | Chlorophyta | Chlorophyta_X | Chlorophyceae | Chlamydomonadales | Chlamydomonadales_X | *Chlamydomonas* | *Chlamydomonas_perpusilla_var._perpusilla* | Phototrophic |
| 3157 | Archaeplastida | Chlorophyta | Chlorophyta_X | Chlorophyceae | Chlamydomonadales | Chlamydomonadales_X | *Chlamydomonas* | *Chlamydomonas_pseudopulsatilla_CCCryo_050-99* | Phototrophic |
| 3162 | Archaeplastida | Chlorophyta | Chlorophyta_X | Chlorophyceae | Chlamydomonadales | Chlamydomonadales_X | *Chlamydomonas* | *Chlamydomonas_raudensis* | Phototrophic |
| 3164 | Archaeplastida | Chlorophyta | Chlorophyta_X | Chlorophyceae | Chlamydomonadales | Chlamydomonadales_X | *Chlamydomonas* | *Chlamydomonas_reinhardtii* | Phototrophic |
| 3165 | Archaeplastida | Chlorophyta | Chlorophyta_X | Chlorophyceae | Chlamydomonadales | Chlamydomonadales_X | *Chlamydomonas* | *Chlamydomonas_rosae* | Phototrophic |
| 3169 | Archaeplastida | Chlorophyta | Chlorophyta_X | Chlorophyceae | Chlamydomonadales | Chlamydomonadales_X | *Chlamydomonas* | *Chlamydomonas_sp.* | Phototrophic |
| 3073 | Archaeplastida | Chlorophyta | Chlorophyta_X | Chlorophyceae | Chlamydomonadales | Chlamydomonadales_X | *Chlorochytrium* | *Chlorochytrium_lemnae* | Phototrophic |
| 3186 | Archaeplastida | Chlorophyta | Chlorophyta_X | Chlorophyceae | Chlamydomonadales | Chlamydomonadales_X | *Chlorococcum* | *Chlorococcum_citriforme* | Phototrophic |
| 76096 | Archaeplastida | Chlorophyta | Chlorophyta_X | Chlorophyceae | Chlamydomonadales | Chlamydomonadales_X | *Chlorococcum* | *Chlorococcum_microstigmatum* | Phototrophic |
| 76097 | Archaeplastida | Chlorophyta | Chlorophyta_X | Chlorophyceae | Chlamydomonadales | Chlamydomonadales_X | *Chlorococcum* | *Chlorococcum_nivale* | Phototrophic |
| 3198 | Archaeplastida | Chlorophyta | Chlorophyta_X | Chlorophyceae | Chlamydomonadales | Chlamydomonadales_X | *Chlorococcum* | *Chlorococcum_sp.* | Phototrophic |
| 3204 | Archaeplastida | Chlorophyta | Chlorophyta_X | Chlorophyceae | Chlamydomonadales | Chlamydomonadales_X | *Chlorogonium* | *Chlorogonium_elongatum* | Phototrophic |
| 3212 | Archaeplastida | Chlorophyta | Chlorophyta_X | Chlorophyceae | Chlamydomonadales | Chlamydomonadales_X | *Chloromonas* | *Chloromonas_carrizoensis* | Phototrophic |
| 76112 | Archaeplastida | Chlorophyta | Chlorophyta_X | Chlorophyceae | Chlamydomonadales | Chlamydomonadales_X | *Chloromonas* | *Chloromonas_chlorococcoides* | Phototrophic |
| 3216 | Archaeplastida | Chlorophyta | Chlorophyta_X | Chlorophyceae | Chlamydomonadales | Chlamydomonadales_X | *Chloromonas* | *Chloromonas_insignis* | Phototrophic |
| 76114 | Archaeplastida | Chlorophyta | Chlorophyta_X | Chlorophyceae | Chlamydomonadales | Chlamydomonadales_X | *Chloromonas* | *Chloromonas_krienitzii* | Phototrophic |
| 3222 | Archaeplastida | Chlorophyta | Chlorophyta_X | Chlorophyceae | Chlamydomonadales | Chlamydomonadales_X | *Chloromonas* | *Chloromonas_palmelloides* | Phototrophic |
| 3228 | Archaeplastida | Chlorophyta | Chlorophyta_X | Chlorophyceae | Chlamydomonadales | Chlamydomonadales_X | *Chloromonas* | *Chloromonas_pseudoplatyrhyncha* | Phototrophic |
| 3230 | Archaeplastida | Chlorophyta | Chlorophyta_X | Chlorophyceae | Chlamydomonadales | Chlamydomonadales_X | *Chloromonas* | *Chloromonas_reticulata* | Phototrophic |
| 3235 | Archaeplastida | Chlorophyta | Chlorophyta_X | Chlorophyceae | Chlamydomonadales | Chlamydomonadales_X | *Chloromonas* | *Chloromonas_schussnigii* | Phototrophic |
| 3237 | Archaeplastida | Chlorophyta | Chlorophyta_X | Chlorophyceae | Chlamydomonadales | Chlamydomonadales_X | *Chloromonas* | *Chloromonas_sp.* | Phototrophic |
| 3241 | Archaeplastida | Chlorophyta | Chlorophyta_X | Chlorophyceae | Chlamydomonadales | Chlamydomonadales_X | *Chloromonas* | *Chloromonas_typhlos* | Phototrophic |
| 3259 | Archaeplastida | Chlorophyta | Chlorophyta_X | Chlorophyceae | Chlamydomonadales | Chlamydomonadales_X | *Cystomonas* | *Cystomonas_sp.* | Phototrophic |
| 3302 | Archaeplastida | Chlorophyta | Chlorophyta_X | Chlorophyceae | Chlamydomonadales | Chlamydomonadales_X | *Haematococcus* | *Haematococcus_sp.* | Phototrophic |
| 3307 | Archaeplastida | Chlorophyta | Chlorophyta_X | Chlorophyceae | Chlamydomonadales | Chlamydomonadales_X | *Hafniomonas* | *Hafniomonas_reticulata* | Phototrophic |
| 3323 | Archaeplastida | Chlorophyta | Chlorophyta_X | Chlorophyceae | Chlamydomonadales | Chlamydomonadales_X | *Lobochlamys* | *Lobochlamys_culleus* | Phototrophic |
| 3324 | Archaeplastida | Chlorophyta | Chlorophyta_X | Chlorophyceae | Chlamydomonadales | Chlamydomonadales_X | *Lobochlamys* | *Lobochlamys_segnis* | Phototrophic |
| 76808 | Archaeplastida | Chlorophyta | Chlorophyta_X | Chlorophyceae | Chlamydomonadales | Chlamydomonadales_X | *Lobochlamys* | *Lobochlamys_sp.* | Phototrophic |
| 76827 | Archaeplastida | Chlorophyta | Chlorophyta_X | Chlorophyceae | Chlamydomonadales | Chlamydomonadales_X | *Macrochloris* | *Macrochloris_rubrioleum* | Phototrophic |
| 3331 | Archaeplastida | Chlorophyta | Chlorophyta_X | Chlorophyceae | Chlamydomonadales | Chlamydomonadales_X | *Neochlorosarcina* | *Neochlorosarcina_negevensis* | Phototrophic |
| 3336 | Archaeplastida | Chlorophyta | Chlorophyta_X | Chlorophyceae | Chlamydomonadales | Chlamydomonadales_X | *Neospongiococcum* | *Neospongiococcum_gelatinosum* | Phototrophic |
| 3340 | Archaeplastida | Chlorophyta | Chlorophyta_X | Chlorophyceae | Chlamydomonadales | Chlamydomonadales_X | *Oogamochlamys* | *Oogamochlamys_zimbabwiensis* | Phototrophic |
| 3345 | Archaeplastida | Chlorophyta | Chlorophyta_X | Chlorophyceae | Chlamydomonadales | Chlamydomonadales_X | *Pandorina* | *Pandorina_morum* | Phototrophic |
| 3359 | Archaeplastida | Chlorophyta | Chlorophyta_X | Chlorophyceae | Chlamydomonadales | Chlamydomonadales_X | *Pleurastrum* | *Pleurastrum_insigne* | Phototrophic |
| 3360 | Archaeplastida | Chlorophyta | Chlorophyta_X | Chlorophyceae | Chlamydomonadales | Chlamydomonadales_X | *Pleurastrum* | *Pleurastrum_sp.* | Phototrophic |
| 3388 | Archaeplastida | Chlorophyta | Chlorophyta_X | Chlorophyceae | Chlamydomonadales | Chlamydomonadales_X | *Tetrabaena* | *Tetrabaena_socialis* | Phototrophic |
| 3391 | Archaeplastida | Chlorophyta | Chlorophyta_X | Chlorophyceae | Chlamydomonadales | Chlamydomonadales_X | *Tetracystis* | *Tetracystis_aplanospora* | Phototrophic |
| 3394 | Archaeplastida | Chlorophyta | Chlorophyta_X | Chlorophyceae | Chlamydomonadales | Chlamydomonadales_X | *Tetracystis* | *Tetracystis_excentrica* | Phototrophic |
| 74347 | Archaeplastida | Chlorophyta | Chlorophyta_X | Chlorophyceae | Chlamydomonadales | Chlamydomonadales_X | *Yamagishiella* | *Yamagishiella_unicocca* | Phototrophic |
| 76168 | Archaeplastida | Chlorophyta | Chlorophyta_X | Chlorophyceae | Chlamydomonadales | Coccomyxaceae | *Coccomyxa* | *Coccomyxa_subellipsoidea* | Phototrophic |
| 3460 | Archaeplastida | Chlorophyta | Chlorophyta_X | Chlorophyceae | Sphaeropleales | Sphaeropleales_X | *Bracteacoccus* | *Bracteacoccus_KF-2012h* | Phototrophic |
| 3072 | Archaeplastida | Chlorophyta | Chlorophyta_X | Chlorophyceae | Sphaeropleales | Sphaeropleales_X | *Bracteacoccus* | *Bracteacoccus_sp.* | Phototrophic |
| 3512 | Archaeplastida | Chlorophyta | Chlorophyta_X | Chlorophyceae | Sphaeropleales | Sphaeropleales_X | *Dictyococcus* | *Dictyococcus_sp.* | Phototrophic |
| 3513 | Archaeplastida | Chlorophyta | Chlorophyta_X | Chlorophyceae | Sphaeropleales | Sphaeropleales_X | *Dictyococcus* | *Dictyococcus_varians* | Phototrophic |
| 3625 | Archaeplastida | Chlorophyta | Chlorophyta_X | Chlorophyceae | Sphaeropleales | Sphaeropleales_X | *Scenedesmus* | *Scenedesmus_sp.* | Phototrophic |
| 3672 | Archaeplastida | Chlorophyta | Chlorophyta_X | Chlorophyta_XX | Chlorophyta_XXX | Chlorophyta_XXXX | *Scotinosphaera* | *Scotinosphaera_gibberosa* | Phototrophic |
| 3674 | Archaeplastida | Chlorophyta | Chlorophyta_X | Chlorophyta_XX | Chlorophyta_XXX | Chlorophyta_XXXX | *Scotinosphaera* | *Scotinosphaera_sp.* | Phototrophic |
| 3698 | Archaeplastida | Chlorophyta | Chlorophyta_X | Mamiellophyceae | Mamiellales | Bathycoccaceae | *Ostreococcus* | *Ostreococcus_sp.* | Phototrophic |
| 49109 | Archaeplastida | Chlorophyta | Chlorophyta_X | Mamiellophyceae | Mamiellales | Mamiellaceae | *Mantoniella* | *Mantoniella_beaufortii* | Phototrophic |
| 49110 | Archaeplastida | Chlorophyta | Chlorophyta_X | Mamiellophyceae | Mamiellales | Mamiellaceae | *Mantoniella* | *Mantoniella_clade_A* | Phototrophic |
| 3704 | Archaeplastida | Chlorophyta | Chlorophyta_X | Mamiellophyceae | Mamiellales | Mamiellaceae | *Mantoniella* | *Mantoniella_squamata* | Phototrophic |
| 49120 | Archaeplastida | Chlorophyta | Chlorophyta_X | Mamiellophyceae | Mamiellales | Mamiellaceae | *Micromonas* | *Micromonas_polaris* | Phototrophic |
| 70121 | Archaeplastida | Chlorophyta | Chlorophyta_X | Pyramimonadophyceae | Pyramimonadales | Pyramimonadaceae | *Pyramimonas* | *Pyramimonas_diskoicola* | Phototrophic |
| 3787 | Archaeplastida | Chlorophyta | Chlorophyta_X | Pyramimonadophyceae | Pyramimonadales | Pyramimonadaceae | *Pyramimonas* | *Pyramimonas_gelidicola* | Phototrophic |
| 3793 | Archaeplastida | Chlorophyta | Chlorophyta_X | Pyramimonadophyceae | Pyramimonadales | Pyramimonadaceae | *Pyramimonas* | *Pyramimonas_sp.* | Phototrophic |
| 3795 | Archaeplastida | Chlorophyta | Chlorophyta_X | Pyramimonadophyceae | Pyramimonadales | Pyramimonadaceae | *Pyramimonas* | *Pyramimonas_tetrarhynchus* | Phototrophic |
| 3797 | Archaeplastida | Chlorophyta | Chlorophyta_X | Trebouxiophyceae | Chlorellales | Chlorellales_X | *Actinastrum* | *Actinastrum_hantzschii* | Phototrophic |
| 3811 | Archaeplastida | Chlorophyta | Chlorophyta_X | Trebouxiophyceae | Chlorellales | Chlorellales_X | *Chlorella* | *Chlorella_sorokiniana* | Phototrophic |
| 3475 | Archaeplastida | Chlorophyta | Chlorophyta_X | Trebouxiophyceae | Chlorellales | Chlorellales_X | *Chlorella* | *Chlorella_sp.* | Phototrophic |
| 3815 | Archaeplastida | Chlorophyta | Chlorophyta_X | Trebouxiophyceae | Chlorellales | Chlorellales_X | *Chlorella* | *Chlorella_vulgaris* | Phototrophic |
| 3818 | Archaeplastida | Chlorophyta | Chlorophyta_X | Trebouxiophyceae | Chlorellales | Chlorellales_X | *Chlorellales_XX* | *Chlorellales_XX_sp.* | Phototrophic |
| 3825 | Archaeplastida | Chlorophyta | Chlorophyta_X | Trebouxiophyceae | Chlorellales | Chlorellales_X | *Coccobotrys* | *Coccobotrys_mucosus* | Phototrophic |
| 3829 | Archaeplastida | Chlorophyta | Chlorophyta_X | Trebouxiophyceae | Chlorellales | Chlorellales_X | *Coenocystis* | *Coenocystis_inconstans* | Phototrophic |
| 3841 | Archaeplastida | Chlorophyta | Chlorophyta_X | Trebouxiophyceae | Chlorellales | Chlorellales_X | *Dictyosphaerium* | *Dictyosphaerium_sp.* | Phototrophic |
| 3873 | Archaeplastida | Chlorophyta | Chlorophyta_X | Trebouxiophyceae | Chlorellales | Chlorellales_X | *Lobosphaera* | *Lobosphaera_tirolensis* | Phototrophic |
| 3879 | Archaeplastida | Chlorophyta | Chlorophyta_X | Trebouxiophyceae | Chlorellales | Chlorellales_X | *Marvania* | *Marvania_sp.* | Phototrophic |
| 3887 | Archaeplastida | Chlorophyta | Chlorophyta_X | Trebouxiophyceae | Chlorellales | Chlorellales_X | *Micractinium* | *Micractinium_sp.* | Phototrophic |
| 3893 | Archaeplastida | Chlorophyta | Chlorophyta_X | Trebouxiophyceae | Chlorellales | Chlorellales_X | *Muriella* | *Muriella_terrestris* | Phototrophic |
| 3899 | Archaeplastida | Chlorophyta | Chlorophyta_X | Trebouxiophyceae | Chlorellales | Chlorellales_X | *Nannochloris* | *Nannochloris_sp.* | Phototrophic |
| 3902 | Archaeplastida | Chlorophyta | Chlorophyta_X | Trebouxiophyceae | Chlorellales | Chlorellales_X | *Neocystis* | *Neocystis_brevis* | Phototrophic |
| 3903 | Archaeplastida | Chlorophyta | Chlorophyta_X | Trebouxiophyceae | Chlorellales | Chlorellales_X | *Neocystis* | *Neocystis_mucosa* | Phototrophic |
| 3904 | Archaeplastida | Chlorophyta | Chlorophyta_X | Trebouxiophyceae | Chlorellales | Chlorellales_X | *Neocystis* | *Neocystis_sp.* | Phototrophic |
| 3922 | Archaeplastida | Chlorophyta | Chlorophyta_X | Trebouxiophyceae | Chlorellales | Chlorellales_X | *Parietochloris* | *Parietochloris_incisa* | Phototrophic |
| 77227 | Archaeplastida | Chlorophyta | Chlorophyta_X | Trebouxiophyceae | Chlorellales | Chlorellales_X | *Parietochloris* | *Parietochloris_pseudoalveolaris* | Phototrophic |
| 3951 | Archaeplastida | Chlorophyta | Chlorophyta_X | Trebouxiophyceae | Chlorellales | Chlorellales_X | *Trochisciopsis* | *Trochisciopsis_tetraspora* | Phototrophic |
| 3963 | Archaeplastida | Chlorophyta | Chlorophyta_X | Trebouxiophyceae | Microthamniales | Microthamniales_X | *Myrmecia* | *Myrmecia_biatorellae* | Phototrophic |
| 3964 | Archaeplastida | Chlorophyta | Chlorophyta_X | Trebouxiophyceae | Microthamniales | Microthamniales_X | *Myrmecia* | *Myrmecia_sp.* | Phototrophic |
| 3971 | Archaeplastida | Chlorophyta | Chlorophyta_X | Trebouxiophyceae | Microthamniales | Microthamniales_X | *Trebouxia* | *Trebouxia_sp.* | Phototrophic |
| 4055 | Archaeplastida | Chlorophyta | Chlorophyta_X | Trebouxiophyceae | Prasiolales | Prasiolales_X | *Elliptochloris* | *Elliptochloris_bilobata* | Phototrophic |
| 4058 | Archaeplastida | Chlorophyta | Chlorophyta_X | Trebouxiophyceae | Prasiolales | Prasiolales_X | *Elliptochloris* | *Elliptochloris_reniformis* | Phototrophic |
| 3660 | Archaeplastida | Chlorophyta | Chlorophyta_X | Trebouxiophyceae | Prasiolales | Prasiolales_X | *Elliptochloris* | *Elliptochloris_sp.* | Phototrophic |
| 3852 | Archaeplastida | Chlorophyta | Chlorophyta_X | Trebouxiophyceae | Prasiolales | Prasiolales_X | *Elliptochloris* | *Elliptochloris_subsphaerica* | Phototrophic |
| 3992 | Archaeplastida | Chlorophyta | Chlorophyta_X | Trebouxiophyceae | Prasiolales | Prasiolales_X | *Koliella* | *Koliella_longiseta* | Phototrophic |
| 4009 | Archaeplastida | Chlorophyta | Chlorophyta_X | Trebouxiophyceae | Prasiolales | Prasiolales_X | *Prasiolales_XX* | *Prasiolales_XX_sp.* | Phototrophic |
| 77443 | Archaeplastida | Chlorophyta | Chlorophyta_X | Trebouxiophyceae | Prasiolales | Prasiolales_X | *Pseudochlorella* | *Pseudochlorella_subsphaerica* | Phototrophic |
| 4016 | Archaeplastida | Chlorophyta | Chlorophyta_X | Trebouxiophyceae | Prasiolales | Prasiolales_X | *Pseudostichococcus* | *Pseudostichococcus_monallantoides* | Phototrophic |
| 77459 | Archaeplastida | Chlorophyta | Chlorophyta_X | Trebouxiophyceae | Prasiolales | Prasiolales_X | *Pseudostichococcus* | *Pseudostichococcus_sp.* | Phototrophic |
| 4026 | Archaeplastida | Chlorophyta | Chlorophyta_X | Trebouxiophyceae | Prasiolales | Prasiolales_X | *Stichococcus* | *Stichococcus_bacillaris* | Phototrophic |
| 3947 | Archaeplastida | Chlorophyta | Chlorophyta_X | Trebouxiophyceae | Prasiolales | Prasiolales_X | *Stichococcus* | *Stichococcus_sp.* | Phototrophic |
| 4082 | Archaeplastida | Chlorophyta | Chlorophyta_X | Trebouxiophyceae | Trebouxiophyceae_X | Trebouxiophyceae_XX | *Trebouxiophyceae_XXX* | *Trebouxiophyceae_XXX_sp.* | Phototrophic |
| 4085 | Archaeplastida | Chlorophyta | Chlorophyta_X | Trebouxiophyceae | Trebouxiophyceae_X | Trebouxiophyceae_XX | *Xylochloris* | *Xylochloris_sp.* | Phototrophic |
| 4095 | Archaeplastida | Chlorophyta | Chlorophyta_X | Trebouxiophyceae | Watanabea-Clade | Watanabea-Clade_X | *Chloroidium* | *Chloroidium_saccharophila* | Phototrophic |
| 3820 | Archaeplastida | Chlorophyta | Chlorophyta_X | Trebouxiophyceae | Watanabea-Clade | Watanabea-Clade_X | *Chloroidium* | *Chloroidium_saccharophilum* | Phototrophic |
| 4097 | Archaeplastida | Chlorophyta | Chlorophyta_X | Trebouxiophyceae | Watanabea-Clade | Watanabea-Clade_X | *Chloroidium* | *Chloroidium_sp.* | Phototrophic |
| 4344 | Archaeplastida | Chlorophyta | Chlorophyta_X | Ulvophyceae | Ulotrichales | Ulotrichales_X | *Chlorothrix* | *Chlorothrix_sp.* | Phototrophic |
| 4348 | Archaeplastida | Chlorophyta | Chlorophyta_X | Ulvophyceae | Ulotrichales | Ulotrichales_X | *Gloeotilopsis* | *Gloeotilopsis_paucicellularis* | Phototrophic |
| 74309 | Archaeplastida | Chlorophyta | Chlorophyta_X | Ulvophyceae | Ulotrichales | Ulotrichales_X | *Hazenia* | *Hazenia_capsulata* | Phototrophic |
| 4352 | Archaeplastida | Chlorophyta | Chlorophyta_X | Ulvophyceae | Ulotrichales | Ulotrichales_X | *Hazenia* | *Hazenia_sp.* | Phototrophic |
| 4359 | Archaeplastida | Chlorophyta | Chlorophyta_X | Ulvophyceae | Ulotrichales | Ulotrichales_X | *Pseudendocloniopsis* | *Pseudendocloniopsis_botryoides* | Phototrophic |
| 4361 | Archaeplastida | Chlorophyta | Chlorophyta_X | Ulvophyceae | Ulotrichales | Ulotrichales_X | *Pseudendoclonium* | *Pseudendoclonium_basiliense* | Phototrophic |
| 4364 | Archaeplastida | Chlorophyta | Chlorophyta_X | Ulvophyceae | Ulotrichales | Ulotrichales_X | *Trichosarcina* | *Trichosarcina_polymorpha* | Phototrophic |
| 4366 | Archaeplastida | Chlorophyta | Chlorophyta_X | Ulvophyceae | Ulotrichales | Ulotrichales_X | *Ulothrix* | *Ulothrix_sp.* | Phototrophic |
| 4367 | Archaeplastida | Chlorophyta | Chlorophyta_X | Ulvophyceae | Ulotrichales | Ulotrichales_X | *Ulothrix* | *Ulothrix_zonata* | Phototrophic |
| 4369 | Archaeplastida | Chlorophyta | Chlorophyta_X | Ulvophyceae | Ulotrichales | Ulotrichales_X | *Urospora* | *Urospora_neglecta* | Phototrophic |
| 4370 | Archaeplastida | Chlorophyta | Chlorophyta_X | Ulvophyceae | Ulotrichales | Ulotrichales_X | *Urospora* | *Urospora_penicilliformis* | Phototrophic |
| 4372 | Archaeplastida | Chlorophyta | Chlorophyta_X | Ulvophyceae | Ulotrichales | Ulotrichales_X | *Urospora* | *Urospora_wormskioldii* | Phototrophic |
| 4524 | Archaeplastida | Rhodophyta | Rhodophyta_X | Bangiophyceae | Cyanidiales | Cyanidiales_X | *Cyanidiales_XX* | *Cyanidiales_XX_sp.* | Phototrophic |
| 5403 | Archaeplastida | Rhodophyta | Rhodophyta_X | Stylonematophyceae | Stylonematales | Stylonemataceae | *Rhodospora* | *Rhodospora_sordida* | Phototrophic |
| 10103 | Cryptista | Cryptophyta | Cryptophyta_X | Cryptophyceae | Cryptomonadales | Cryptomonadales_X | *Cryptomonadales_XX* | *Cryptomonadales_XX_sp.* | Mixotrophic |
| 10122 | Cryptista | Cryptophyta | Cryptophyta_X | Cryptophyceae | Cryptomonadales | Cryptomonadales_X | *Cryptomonas* | *Cryptomonas_pyrenoidifera* | Mixotrophic |
| 10125 | Cryptista | Cryptophyta | Cryptophyta_X | Cryptophyceae | Cryptomonadales | Cryptomonadales_X | *Cryptomonas* | *Cryptomonas_sp.* | Mixotrophic |
| 10128 | Cryptista | Cryptophyta | Cryptophyta_X | Cryptophyceae | Cryptomonadales | Cryptomonadales_X | *Falcomonas* | *Falcomonas_daucoides* | Mixotrophic |
| 10130 | Cryptista | Cryptophyta | Cryptophyta_X | Cryptophyceae | Cryptomonadales | Cryptomonadales_X | *Geminigera* | *Geminigera_cryophila* | Mixotrophic |
| 75692 | Cryptista | Cryptophyta | Cryptophyta_X | Cryptophyceae | Cryptomonadales | Hemiselmidaceae | *Chroomonas* | *Chroomonas_debatzensis* | Mixotrophic |
| 10101 | Cryptista | Cryptophyta | Cryptophyta_X | Cryptophyceae | Cryptomonadales | Hemiselmidaceae | *Chroomonas* | *Chroomonas_sp.* | Mixotrophic |
| 10138 | Cryptista | Cryptophyta | Cryptophyta_X | Cryptophyceae | Cryptomonadales | Hemiselmidaceae | *Hemiselmis* | *Hemiselmis_sp.* | Mixotrophic |
| 10139 | Cryptista | Cryptophyta | Cryptophyta_X | Cryptophyceae | Cryptomonadales | Hemiselmidaceae | *Hemiselmis* | *Hemiselmis_tepida* | Mixotrophic |
| 10140 | Cryptista | Cryptophyta | Cryptophyta_X | Cryptophyceae | Cryptomonadales | Hemiselmidaceae | *Hemiselmis* | *Hemiselmis_virescens* | Mixotrophic |
| 10170 | Cryptista | Cryptophyta | Cryptophyta_X | Cryptophyceae | Goniomonadales | Goniomonadales_X | *Goniomonadales_XX* | *Goniomonadales_XX_sp.* | Bacterivore |
| 10171 | Cryptista | Cryptophyta | Cryptophyta_X | Cryptophyceae | Goniomonadales | Goniomonadales_X | *Goniomonas* | *Goniomonas_amphinema* | Bacterivore |
| 10174 | Cryptista | Cryptophyta | Cryptophyta_X | Cryptophyceae | Goniomonadales | Goniomonadales_X | *Goniomonas* | *Goniomonas_sp.* | Bacterivore |
| 50716 | Cryptista | Cryptophyta | Cryptophyta_X | Cryptophyceae | Pyrenomonadales | Baffinellaceae | *Baffinella* | *Baffinella_frigidus* | Mixotrophic |
| 10150 | Cryptista | Cryptophyta | Cryptophyta_X | Cryptophyceae | Pyrenomonadales | Pyrenomonadaceae | *Rhodomonas* | *Rhodomonas_abbreviata* | Phototrophic |
| 10160 | Cryptista | Cryptophyta | Cryptophyta_X | Cryptophyceae | Pyrenomonadales | Pyrenomonadaceae | *Rhodomonas* | *Rhodomonas_sp.* | Phototrophic |
| 10328 | Cryptista | Kathablepharidacea | Kathablepharida | Kathablepharidea | Kathablepharidida | Kathablepharidida_X | *Kathablepharidida_XX* | *Kathablepharidida_XX_sp.* | Eukaryvore |
| 73536 | Eukaryota_X | Hemimastigophora | Hemimastigophora_X | Hemimastigophora_XX | Hemimastigophora_XXX | Spironemidae | *Hemimastix* | *Hemimastix_sp.* | NA |
| 71697 | Excavata | Discoba | Discoba_X | Discoba_XX | Jakobida | Moramonadidae | *Seculamonas* | *Seculamonas_ecuadoriensis* | Bacterivore |
| 71190 | Excavata | Discoba | Discoba_X | Heterolobosea | Acrasida | Acrasidae | *Allovahlkampfia* | *Allovahlkampfia_sp.* | Bacterivore |
| 71732 | Excavata | Discoba | Discoba_X | Heterolobosea | Percolatea | Stephanopogonidae | *Stephanopogon* | *Stephanopogon_apogon* | Bacterivore |
| 71572 | Excavata | Discoba | Discoba_X | Heterolobosea | Tetramitia_I | Neovahlkampfiidae | *Neovahlkampfia* | *Neovahlkampfia_sp.* | Bacterivore |
| 71903 | Excavata | Discoba | Discoba_X | Heterolobosea | Tetramitia_V | Vahlkampfiidae_V | *Vahlkampfia* | *Vahlkampfia_avara* | Bacterivore |
| 71316 | Excavata | Discoba | Euglenozoa | Euglenida | Euglenophyceae | Euglenaceae | *Euglena* | *Euglena_geniculata* | Mixotrophic |
| 71795 | Excavata | Discoba | Euglenozoa | Euglenida | Euglenophyceae | Euglenaceae | *Trachelomonas* | *Trachelomonas_lefevrei* | Mixotrophic |
| 71606 | Excavata | Discoba | Euglenozoa | Euglenida | Euglenophyceae | Phacaceae | *Phacus* | *Phacus_acuminatus* | Mixotrophic |
| 71636 | Excavata | Discoba | Euglenozoa | Euglenida | Euglenophyceae | Phacaceae | *Phacus* | *Phacus_raciborskii* | Mixotrophic |
| 71347 | Excavata | Discoba | Euglenozoa | Euglenida | Petalomonadida | Eupetalomonads_X | *Eupetalomonads_XX* | *Eupetalomonads_XX_sp.* | Bacterivore |
| 71575 | Excavata | Discoba | Euglenozoa | Euglenida | Petalomonadida | Scytomonadidae | *Notosolenus* | *Notosolenus_urceolatus* | Bacterivore |
| 71602 | Excavata | Discoba | Euglenozoa | Euglenida | Petalomonadida | Scytomonadidae | *Petalomonas* | *Petalomonas_cantuscygni* | Bacterivore |
| 71440 | Excavata | Discoba | Euglenozoa | Kinetoplastea | Kinetoplastea_X | Kinetoplastea_XX | *Kinetoplastea_XX_MET6* | *Kinetoplastea_XX_MET6_sp.* | NA |
| 71444 | Excavata | Discoba | Euglenozoa | Kinetoplastea | Kinetoplastea_X | Kinetoplastea_XX | *Kinetoplastea_XXX* | *Kinetoplastea_XXX_sp.* | NA |
| 71568 | Excavata | Discoba | Euglenozoa | Kinetoplastea | Neobodonida | Neobodonidae | *Neobodo* | *Neobodo_sp.* | Bacterivore |
| 71661 | Excavata | Discoba | Euglenozoa | Kinetoplastea | Parabodonida | Parabodonidae | *Procryptobia* | *Procryptobia_sorokini* | Bacterivore |
| 71679 | Excavata | Metamonada | Fornicata | Fornicata_X | Fornicata_XX | Retortamonadidae | *Retortamonas* | *Retortamonas_sp.* | Animal Parasite |
| 10064 | Haptista | Centroplasthelida | Centroplasthelida_X | Centroplasthelida_XX | Centroplasthelida_XXX | Centroplasthelida_XXXX | *Centroplasthelida_XXXXX* | *Centroplasthelida_XXXXX_sp.* | Omnivore |
| 10185 | Haptista | Haptophyta | Haptophyta_X | Pavlovophyceae | Pavlovales | Pavlovaceae | *Diacronema* | *Diacronema_lutheri* | Phototrophic |
| 10187 | Haptista | Haptophyta | Haptophyta_X | Pavlovophyceae | Pavlovales | Pavlovaceae | *Diacronema* | *Diacronema_sp.* | Phototrophic |
| 10190 | Haptista | Haptophyta | Haptophyta_X | Pavlovophyceae | Pavlovales | Pavlovaceae | *Diacronema* | *Diacronema_vlkianum* | Phototrophic |
| 10315 | Haptista | Haptophyta | Haptophyta_X | Prymnesiophyceae | Prymnesiales | Prymnesiaceae | *Pseudohaptolina* | *Pseudohaptolina_birgeri* | Phototrophic |
| 2977 | Obazoa | Apusomonada | Apusomonada_X | Apusomonadidae | Apusomonadidae_Group-1 | Apusomonadidae_Group-1_X | *Apusomonas* | *Apusomonas_proboscidea* | Bacterivore |
| 10371 | Obazoa | Opisthokonta | Choanoflagellata | Choanoflagellatea | Acanthoecida | Stephanoecidae_Group_D | *Stephanoecidae_Group_D_X* | *Stephanoecidae_Group_D_X_sp.* | Bacterivore |
| 10415 | Obazoa | Opisthokonta | Choanoflagellata | Choanoflagellatea | Craspedida | Monosigidae_Group_O | *Monosigidae_Group_O_X* | *Monosigidae_Group_O_X_sp.* | Bacterivore |
| 10421 | Obazoa | Opisthokonta | Choanoflagellata | Choanoflagellatea | Craspedida | Salpingoecidae_Group_C1 | *Salpingoecidae_Group_C1_X* | *Salpingoecidae_Group_C1_X_sp.* | Bacterivore |
| 18944 | Obazoa | Opisthokonta | Ichthyosporea | Ichthyophonida | Pseudoperkinsidae | Pseudoperkinsidae_X | *Anurofeca* | *Anurofeca_richardsi* | Animal Parasite |
| 18945 | Obazoa | Opisthokonta | Ichthyosporea | Ichthyophonida | Pseudoperkinsidae | Pseudoperkinsidae_X | *Anurofeca* | *Anurofeca_sp.* | Animal Parasite |
| 18947 | Obazoa | Opisthokonta | Ichthyosporea | Ichthyophonida | Pseudoperkinsidae | Pseudoperkinsidae_X | *Pseudoperkinsidae_XX* | *Pseudoperkinsidae_XX_sp.* | Animal Parasite |
| 45036 | Obazoa | Opisthokonta | Opisthokonta_X | Opisthokonta_XX | Opisthokonta_XXX | Opisthokonta_XXXX | *Opisthokonta_XXXXX* | *Opisthokonta_XXXXX_sp.* | NA |
| 18972 | Obazoa | Opisthokonta | Rotosphaerida | Nucleariidea | Nucleariida | Nucleariida_X | *Nuclearia* | *Nuclearia_pattersoni* | Pathogen |
| 1 | TSAR | Alveolata | Alveolata_X | Alveolata_XX | Alveolata_XXX | Alveolata_XXXX | *Alveolata_XXXXX* | *Alveolata_XXXXX_sp.* | NA |
| 73990 | TSAR | Alveolata | Apicomplexa | Gregarinomorphea | Eugregarinorida | Eugregarinorida_EUG1 | *Eugregarinorida_EUG1_X* | *Eugregarinorida_EUG1_X_sp.* | Animal Parasite |
| 73992 | TSAR | Alveolata | Apicomplexa | Gregarinomorphea | Eugregarinorida | Eugregarinorida_EUG3 | *Eugregarinorida_EUG3_X* | *Eugregarinorida_EUG3_X_sp.* | Animal Parasite |
| 74025 | TSAR | Alveolata | Apicomplexa | Gregarinomorphea | Eugregarinorida | Lecudinidae | *Lankesteria* | *Lankesteria_chelyosomae* | Animal Parasite |
| 74026 | TSAR | Alveolata | Apicomplexa | Gregarinomorphea | Eugregarinorida | Lecudinidae | *Lankesteria* | *Lankesteria_cystodytae* | Animal Parasite |
| 74027 | TSAR | Alveolata | Apicomplexa | Gregarinomorphea | Eugregarinorida | Lecudinidae | *Lecudina* | *Lecudina_longissima* | Animal Parasite |
| 74028 | TSAR | Alveolata | Apicomplexa | Gregarinomorphea | Eugregarinorida | Lecudinidae | *Lecudina* | *Lecudina_phyllochaetopteri* | Animal Parasite |
| 74029 | TSAR | Alveolata | Apicomplexa | Gregarinomorphea | Eugregarinorida | Lecudinidae | *Lecudina* | *Lecudina_tuzetae* | Animal Parasite |
| 74030 | TSAR | Alveolata | Apicomplexa | Gregarinomorphea | Eugregarinorida | Lecudinidae | *Lecudinidae_X* | *Lecudinidae_X_sp.* | Animal Parasite |
| 74034 | TSAR | Alveolata | Apicomplexa | Gregarinomorphea | Eugregarinorida | Lecudinidae | *Urosporids* | *Urosporids_sp.* | Animal Parasite |
| 74052 | TSAR | Alveolata | Apicomplexa | Gregarinomorphea | Eugregarinorida | Urosporidae | *Difficilina* | *Difficilina_tubulani* | Animal Parasite |
| 75507 | TSAR | Alveolata | Apicomplexa | Gregarinomorphea | Gregarinomorphea_X | Gregarinomorphea_XX | *Selenidium* | *Selenidium_oshoroense* | Animal Parasite |
| 75513 | TSAR | Alveolata | Apicomplexa | Gregarinomorphea | Gregarinomorphea_X | Gregarinomorphea_XX | *Selenidium* | *Selenidium_sabellariae* | Animal Parasite |
| 74057 | TSAR | Alveolata | Apicomplexa | Gregarinomorphea | Gregarinomorphea_X | Gregarinomorphea_XX | *Selenidium1* | *Selenidium1_echinatum* | Animal Parasite |
| 74059 | TSAR | Alveolata | Apicomplexa | Gregarinomorphea | Gregarinomorphea_X | Gregarinomorphea_XX | *Selenidium1* | *Selenidium1_mesnili* | Animal Parasite |
| 74061 | TSAR | Alveolata | Apicomplexa | Gregarinomorphea | Gregarinomorphea_X | Gregarinomorphea_XX | *Selenidium1* | *Selenidium1_sensimae* | Animal Parasite |
| 74063 | TSAR | Alveolata | Apicomplexa | Gregarinomorphea | Gregarinomorphea_X | Gregarinomorphea_XX | *Selenidium1* | *Selenidium1_sp.* | Animal Parasite |
| 74065 | TSAR | Alveolata | Apicomplexa | Gregarinomorphea | Gregarinomorphea_X | Gregarinomorphea_XX | *Selenidium2* | *Selenidium2_pisinnus* | Animal Parasite |
| 74074 | TSAR | Alveolata | Apicomplexa | Gregarinomorphea | Gregarinomorphea_X_GRE6 | Gregarinomorphea_X_GRE6_X | *Gregarinomorphea_X_GRE6_XX* | *Gregarinomorphea_X_GRE6_XX_sp.* | Animal Parasite |
| 74085 | TSAR | Alveolata | Apicomplexa | Gregarinomorphea | Neogregarinorida | Actinocephalidae | *Hyplorynchus* | *Hyplorynchus_sp.* | Animal Parasite |
| 74111 | TSAR | Alveolata | Chrompodellids | Colpodellidea | Colpodellida | Alphamonaceae | *Alphamonas* | *Alphamonas_sp.* | Eukaryvore |
| 675 | TSAR | Alveolata | Ciliophora | Ciliophora_X | Ciliophora_XX | Ciliophora_XXX | *Mesodinium* | *Mesodinium_pulex* | Mixotrophic |
| 678 | TSAR | Alveolata | Ciliophora | Ciliophora_X | Ciliophora_XX | Ciliophora_XXX | *Mesodinium* | *Mesodinium_sp.* | Mixotrophic |
| 514 | TSAR | Alveolata | Ciliophora | Colpodea | Colpodea_X | Bursariomorphida | *Bryometopus* | *Bryometopus_atypicus* | Bacterivore |
| 515 | TSAR | Alveolata | Ciliophora | Colpodea | Colpodea_X | Bursariomorphida | *Bryometopus* | *Bryometopus_pseudochilodon* | Bacterivore |
| 517 | TSAR | Alveolata | Ciliophora | Colpodea | Colpodea_X | Bursariomorphida | *Bryometopus* | *Bryometopus_sphagni* | Bacterivore |
| 520 | TSAR | Alveolata | Ciliophora | Colpodea | Colpodea_X | Bursariomorphida | *Bryometopus* | *Bryometopus_triquetrus* | Bacterivore |
| 49141 | TSAR | Alveolata | Ciliophora | Colpodea | Colpodea_X | Bursariomorphida | *Bursariomorphida_X* | *Bursariomorphida_X_sp.* | Bacterivore |
| 556 | TSAR | Alveolata | Ciliophora | Colpodea | Colpodea_X | Colpodida | *Bresslauides* | *Bresslauides_discoideus* | Bacterivore |
| 527 | TSAR | Alveolata | Ciliophora | Colpodea | Colpodea_X | Colpodida | *Bryophrya* | *Bryophrya_gemmea* | Bacterivore |
| 49142 | TSAR | Alveolata | Ciliophora | Colpodea | Colpodea_X | Colpodida | *Colpodida_X* | *Colpodida_X_sp.* | Bacterivore |
| 559 | TSAR | Alveolata | Ciliophora | Colpodea | Colpodea_X | Colpodida | *Maryna* | *Maryna_ovata* | Bacterivore |
| 554 | TSAR | Alveolata | Ciliophora | Colpodea | Colpodea_X | Colpodida | *Mykophagophrys* | *Mykophagophrys_terricola* | Bacterivore |
| 555 | TSAR | Alveolata | Ciliophora | Colpodea | Colpodea_X | Colpodida | *Pseudoplatyophrya* | *Pseudoplatyophrya_nana* | Bacterivore |
| 569 | TSAR | Alveolata | Ciliophora | Colpodea | Colpodea_X | Cyrtolophosidida | *Pseudocyrtolophosis* | *Pseudocyrtolophosis_alpestris* | Bacterivore |
| 571 | TSAR | Alveolata | Ciliophora | Colpodea | Colpodea_X | Platyophryida | *Etoschophrya* | *Etoschophrya_inornata* | Eukaryvore |
| 576 | TSAR | Alveolata | Ciliophora | Colpodea | Colpodea_X | Platyophryida | *Platyophrya* | *Platyophrya_sp.* | Eukaryvore |
| 584 | TSAR | Alveolata | Ciliophora | Colpodea | Colpodea_X | Platyophryida | *Woodruffides* | *Woodruffides_metabolicus* | Eukaryvore |
| 49153 | TSAR | Alveolata | Ciliophora | CONTH_4 | CONTH_4_X | CONTH_4_XX | *CONTH_4_XXX* | *CONTH_4_XXX_sp.* | NA |
| 671 | TSAR | Alveolata | Ciliophora | CONThreeP | CONThreeP_X | CONThreeP_XX | *Askenasia* | *Askenasia_sp.* | Eukaryvore |
| 49158 | TSAR | Alveolata | Ciliophora | CONThreeP | CONThreeP_X | CONThreeP_XX | *CONThreeP_XXX* | *CONThreeP_XXX_sp.* | NA |
| 588 | TSAR | Alveolata | Ciliophora | Heterotrichea | Heterotrichea_X | Blepharismidae | *Blepharisma* | *Blepharisma_elongatum* | Bacterivore |
| 49162 | TSAR | Alveolata | Ciliophora | Heterotrichea | Heterotrichea_X | Blepharismidae | *Blepharisma* | *Blepharisma_penardi* | Bacterivore |
| 49163 | TSAR | Alveolata | Ciliophora | Heterotrichea | Heterotrichea_X | Blepharismidae | *Blepharisma* | *Blepharisma_sp.* | Bacterivore |
| 594 | TSAR | Alveolata | Ciliophora | Heterotrichea | Heterotrichea_X | Blepharismidae | *Blepharisma* | *Blepharisma_undulans* | Bacterivore |
| 596 | TSAR | Alveolata | Ciliophora | Heterotrichea | Heterotrichea_X | Climacostomidae | *Climacostomum* | *Climacostomum_virens* | Omnivore |
| 618 | TSAR | Alveolata | Ciliophora | Heterotrichea | Heterotrichea_X | Condylostomatidae | *Condylostentor* | *Condylostentor_auriculatus* | Bacterivore |
| 600 | TSAR | Alveolata | Ciliophora | Heterotrichea | Heterotrichea_X | Condylostomatidae | *Condylostoma* | *Condylostoma_sp.* | Bacterivore |
| 602 | TSAR | Alveolata | Ciliophora | Heterotrichea | Heterotrichea_X | Condylostomatidae | *Condylostomides* | *Condylostomides_sp.* | Bacterivore |
| 622 | TSAR | Alveolata | Ciliophora | Heterotrichea | Heterotrichea_X | Stentoridae | *Stentor* | *Stentor_elegans* | Omnivore |
| 75440 | TSAR | Alveolata | Ciliophora | Litostomatea | Haptoria_3 | Haptoria_3_X | *Fuscheria* | *Fuscheria_nodosa* | Bacterivore |
| 49193 | TSAR | Alveolata | Ciliophora | Litostomatea | Haptoria_3 | Haptoria_3_X | *Haptoria_3_XX* | *Haptoria_3_XX_sp.* | Bacterivore |
| 687 | TSAR | Alveolata | Ciliophora | Litostomatea | Haptoria_4 | Didiniidae | *Didiniidae_X* | *Didiniidae_X_sp.* | Bacterivore |
| 698 | TSAR | Alveolata | Ciliophora | Litostomatea | Haptoria_6 | Lacrymariidae | *Lacrymariidae_X* | *Lacrymariidae_X_sp.* | Bacterivore |
| 75449 | TSAR | Alveolata | Ciliophora | Litostomatea | Haptoria_7 | Spathidiida | *Paraenchelys* | *Paraenchelys_terricola* | Bacterivore |
| 49205 | TSAR | Alveolata | Ciliophora | Litostomatea | Litostomatea_X | Litostomatea_XX | *Epispathidium* | *Epispathidium_terricola* | NA |
| 752 | TSAR | Alveolata | Ciliophora | Litostomatea | Litostomatea_X | Litostomatea_XX | *Lagynophrya* | *Lagynophrya_acuminata* | Omnivore |
| 755 | TSAR | Alveolata | Ciliophora | Litostomatea | Litostomatea_X | Litostomatea_XX | *Litostomatea_XXX* | *Litostomatea_XXX_sp.* | Bacterivore |
| 729 | TSAR | Alveolata | Ciliophora | Litostomatea | Litostomatea_X | Litostomatea_XX | *Spathidium* | *Spathidium_foissneri* | Bacterivore |
| 49210 | TSAR | Alveolata | Ciliophora | Litostomatea | Litostomatea_X | Litostomatea_XX | *Spathidium* | *Spathidium_papilliferum* | Bacterivore |
| 733 | TSAR | Alveolata | Ciliophora | Litostomatea | Litostomatea_X | Litostomatea_XX | *Spathidium* | *Spathidium_stammeri* | Bacterivore |
| 49213 | TSAR | Alveolata | Ciliophora | Litostomatea | Litostomatea_X | Litostomatea_XX | *Trachelophyllum* | *Trachelophyllum_brachypharynx* | Bacterivore |
| 734 | TSAR | Alveolata | Ciliophora | Litostomatea | Rhynchostomatia | Dileptidae | *Apodileptus* | *Apodileptus_visscheri* | Bacterivore |
| 49214 | TSAR | Alveolata | Ciliophora | Litostomatea | Rhynchostomatia | Dileptidae | *Dileptidae_X* | *Dileptidae_X_sp.* | Bacterivore |
| 735 | TSAR | Alveolata | Ciliophora | Litostomatea | Rhynchostomatia | Dileptidae | *Dileptus* | *Dileptus_costaricanus* | Bacterivore |
| 736 | TSAR | Alveolata | Ciliophora | Litostomatea | Rhynchostomatia | Dileptidae | *Dileptus* | *Dileptus_microstoma* | Bacterivore |
| 49221 | TSAR | Alveolata | Ciliophora | Litostomatea | Rhynchostomatia | Dileptidae | *Pseudomonilicaryon* | *Pseudomonilicaryon_fraterculum* | Bacterivore |
| 744 | TSAR | Alveolata | Ciliophora | Litostomatea | Rhynchostomatia | Tracheliidae | *Trachelius* | *Trachelius_ovum* | Omnivore |
| 866 | TSAR | Alveolata | Ciliophora | Litostomatea | Trichostomatia | Trichostomatia_X | *Trichostomatia_XX* | *Trichostomatia_XX_sp.* | Animal Parasite |
| 868 | TSAR | Alveolata | Ciliophora | Nassophorea | Nassophorea_X | Colpodidiidae | *Colpodidiidae_X* | *Colpodidiidae_X_sp.* | Omnivore |
| 871 | TSAR | Alveolata | Ciliophora | Nassophorea | Nassophorea_X | Nassulida | *Furgasonia* | *Furgasonia_blochmanni* | Eukaryvore |
| 49246 | TSAR | Alveolata | Ciliophora | Nassophorea | Nassophorea_X | Nassulida | *Nassulida_X* | *Nassulida_X_sp.* | Eukaryvore |
| 75371 | TSAR | Alveolata | Ciliophora | Nassophorea | Nassophorea_X | Nassulida | *Naxella* | *Naxella_paralucida* | Eukaryvore |
| 880 | TSAR | Alveolata | Ciliophora | Nassophorea | Nassophorea_X | Nassulida | *Obertrumia* | *Obertrumia_georgiana* | Eukaryvore |
| 75372 | TSAR | Alveolata | Ciliophora | Oligohymenophorea | Astomatia | Astomatida | *Anoplophrya* | *Anoplophrya_nodulata* | Animal Parasite |
| 75375 | TSAR | Alveolata | Ciliophora | Oligohymenophorea | Astomatia | Astomatida | *Metaradiophrya* | *Metaradiophrya_lumbrici* | Animal Parasite |
| 75376 | TSAR | Alveolata | Ciliophora | Oligohymenophorea | Astomatia | Astomatida | *Metaradiophrya* | *Metaradiophrya_varians* | Animal Parasite |
| 898 | TSAR | Alveolata | Ciliophora | Oligohymenophorea | Hymenostomatia | Ophryoglenida | *Ichthyophthirius* | *Ichthyophthirius_multifiliis* | Animal Parasite |
| 899 | TSAR | Alveolata | Ciliophora | Oligohymenophorea | Hymenostomatia | Ophryoglenida | *Ophryoglena* | *Ophryoglena_catenula* | Animal Parasite |
| 49259 | TSAR | Alveolata | Ciliophora | Oligohymenophorea | Hymenostomatia | Tetrahymenida | *Tetrahymenida_X* | *Tetrahymenida_X_sp.* | Animal Parasite |
| 1027 | TSAR | Alveolata | Ciliophora | Oligohymenophorea | Oligohymenophorea_X | Oligohymenophorea_XX | *Cardiostomatella* | *Cardiostomatella_vermiformis* | Animal Parasite |
| 49278 | TSAR | Alveolata | Ciliophora | Oligohymenophorea | Peniculia | Frontoniidae_2 | *Frontoniidae_2_X* | *Frontoniidae_2_X_sp.* | Omnivore |
| 941 | TSAR | Alveolata | Ciliophora | Oligohymenophorea | Peniculia | Parameciidae | *Paramecium* | *Paramecium_caudatum* | Omnivore |
| 75381 | TSAR | Alveolata | Ciliophora | Oligohymenophorea | Peniculia | Peniculia_X | *Frontonia* | *Frontonia_terricola* | Omnivore |
| 49289 | TSAR | Alveolata | Ciliophora | Oligohymenophorea | Peritrichia_2 | OLIGO2 | *OLIGO2_X* | *OLIGO2_X_sp.* | Omnivore |
| 49292 | TSAR | Alveolata | Ciliophora | Oligohymenophorea | Peritrichia_2 | Operculariidae | *Operculariidae_X* | *Operculariidae_X_sp.* | Omnivore |
| 961 | TSAR | Alveolata | Ciliophora | Oligohymenophorea | Peritrichia_2 | Sessilida | *Astylozoon* | *Astylozoon_enriquesi* | Omnivore |
| 75387 | TSAR | Alveolata | Ciliophora | Oligohymenophorea | Peritrichia_2 | Sessilida | *Cothurnia* | *Cothurnia_salina* | Omnivore |
| 1105 | TSAR | Alveolata | Ciliophora | Oligohymenophorea | Peritrichia_2 | Sessilida | *Epicarchesium* | *Epicarchesium_abrae* | Omnivore |
| 973 | TSAR | Alveolata | Ciliophora | Oligohymenophorea | Peritrichia_2 | Sessilida | *Ophrydium* | *Ophrydium_versatile* | Omnivore |
| 975 | TSAR | Alveolata | Ciliophora | Oligohymenophorea | Peritrichia_2 | Sessilida | *Opisthonecta* | *Opisthonecta_minima* | Omnivore |
| 1106 | TSAR | Alveolata | Ciliophora | Oligohymenophorea | Peritrichia_2 | Sessilida | *Pseudovorticella* | *Pseudovorticella_paracratera* | Omnivore |
| 1107 | TSAR | Alveolata | Ciliophora | Oligohymenophorea | Peritrichia_2 | Sessilida | *Pseudovorticella* | *Pseudovorticella_punctata* | Omnivore |
| 49305 | TSAR | Alveolata | Ciliophora | Oligohymenophorea | Peritrichia_2 | Sessilida | *Sessilida_X* | *Sessilida_X_sp.* | Omnivore |
| 1113 | TSAR | Alveolata | Ciliophora | Oligohymenophorea | Peritrichia_2 | Sessilida | *Vorticella* | *Vorticella_convallaria* | Omnivore |
| 1117 | TSAR | Alveolata | Ciliophora | Oligohymenophorea | Peritrichia_2 | Sessilida | *Vorticella* | *Vorticella_microstoma* | Omnivore |
| 1124 | TSAR | Alveolata | Ciliophora | Oligohymenophorea | Peritrichia_2 | Sessilida | *Vorticellides* | *Vorticellides_astyliformis* | Omnivore |
| 986 | TSAR | Alveolata | Ciliophora | Oligohymenophorea | Peritrichia_2 | Vaginicolidae | *Vaginicolidae_X* | *Vaginicolidae_X_sp.* | Omnivore |
| 49320 | TSAR | Alveolata | Ciliophora | Oligohymenophorea | Scuticociliatia_1 | Philasterida | *Philasterida_X* | *Philasterida_X_sp.* | Omnivore |
| 49324 | TSAR | Alveolata | Ciliophora | Oligohymenophorea | Scuticociliatia_1 | Thigmophryidae | *Myxophyllum* | *Myxophyllum_sp.* | Omnivore |
| 49336 | TSAR | Alveolata | Ciliophora | Oligohymenophorea | Scuticociliatia_2 | Pleuronematidae_2 | *Pleuronema_2* | *Pleuronema_2_sp.* | Omnivore |
| 49342 | TSAR | Alveolata | Ciliophora | Oligohymenophorea | Scuticociliatia_2 | Scuticociliatia_2_X | *Protocyclidium* | *Protocyclidium_sinica* | Omnivore |
| 49343 | TSAR | Alveolata | Ciliophora | Oligohymenophorea | Scuticociliatia_2 | Scuticociliatia_2_X | *Scuticociliatia_2_XX* | *Scuticociliatia_2_XX_sp.* | Omnivore |
| 49345 | TSAR | Alveolata | Ciliophora | Phyllopharyngea | Cyrtophoria_1 | PHYLL_4 | *PHYLL_4_X* | *PHYLL_4_X_sp.* | Omnivore |
| 49350 | TSAR | Alveolata | Ciliophora | Phyllopharyngea | Cyrtophoria_3 | Lynchellidae_2 | *Lynchellidae_2_X* | *Lynchellidae_2_X_sp.* | Omnivore |
| 1180 | TSAR | Alveolata | Ciliophora | Phyllopharyngea | Cyrtophoria_4 | Chilodonellidae | *Chilodonellidae_X* | *Chilodonellidae_X_sp.* | Omnivore |
| 75405 | TSAR | Alveolata | Ciliophora | Phyllopharyngea | Cyrtophoria_4 | Chilodonellidae | *Odontochlamys* | *Odontochlamys_alpestris* | Omnivore |
| 75406 | TSAR | Alveolata | Ciliophora | Phyllopharyngea | Cyrtophoria_4 | Chilodonellidae | *Phascolodon* | *Phascolodon_vorticella* | Omnivore |
| 1181 | TSAR | Alveolata | Ciliophora | Phyllopharyngea | Cyrtophoria_4 | Chilodonellidae | *Pseudochilodonopsis* | *Pseudochilodonopsis_fluviatilis* | Omnivore |
| 49356 | TSAR | Alveolata | Ciliophora | Phyllopharyngea | Cyrtophoria_4 | Chilodonellidae | *Trithigmostoma* | *Trithigmostoma_cucullulus* | Omnivore |
| 1183 | TSAR | Alveolata | Ciliophora | Phyllopharyngea | Cyrtophoria_4 | Chilodonellidae | *Trithigmostoma* | *Trithigmostoma_steini* | Omnivore |
| 1228 | TSAR | Alveolata | Ciliophora | Phyllopharyngea | Suctoria | Ephelotidae | *Ephelota* | *Ephelota_sp.* | Bacterivore |
| 49375 | TSAR | Alveolata | Ciliophora | Phyllopharyngea | Suctoria | PHYLL_2 | *PHYLL_2_X* | *PHYLL_2_X_sp.* | Bacterivore |
| 1232 | TSAR | Alveolata | Ciliophora | Phyllopharyngea | Suctoria | Suctoria_X | *Suctoria_XX* | *Suctoria_XX_sp.* | Bacterivore |
| 49377 | TSAR | Alveolata | Ciliophora | Phyllopharyngea | Suctoria | Tokophryidae | *Tokophrya* | *Tokophrya_huangmeiensis* | Bacterivore |
| 1233 | TSAR | Alveolata | Ciliophora | Phyllopharyngea | Suctoria | Tokophryidae | *Tokophrya* | *Tokophrya_infusionum* | Bacterivore |
| 1234 | TSAR | Alveolata | Ciliophora | Phyllopharyngea | Suctoria | Tokophryidae | *Tokophrya* | *Tokophrya_lemnarum* | Bacterivore |
| 1235 | TSAR | Alveolata | Ciliophora | Phyllopharyngea | Suctoria | Tokophryidae | *Tokophrya* | *Tokophrya_quadripartita* | Bacterivore |
| 1236 | TSAR | Alveolata | Ciliophora | Phyllopharyngea | Suctoria | Tokophryidae | *Tokophrya* | *Tokophrya_sp.* | Bacterivore |
| 49161 | TSAR | Alveolata | Ciliophora | Prostomatea | Prorodontida | Urotrichidae | *Urotricha* | *Urotricha_sp.* | Eukaryvore |
| 74195 | TSAR | Alveolata | Ciliophora | Spirotrichea | Choreotrichida | Strobilidiidae | *Rimostrombidium* | *Rimostrombidium_sp.* | Omnivore |
| 48679 | TSAR | Alveolata | Ciliophora | Spirotrichea | Choreotrichida | Strobilidiidae | *Strobilidiidae_X* | *Strobilidiidae_X_sp.* | Omnivore |
| 48887 | TSAR | Alveolata | Ciliophora | Spirotrichea | Choreotrichida-Tintinnina | TIN_03 | *Tintinnopsis_03* | *Tintinnopsis_03_sp.* | Omnivore |
| 48929 | TSAR | Alveolata | Ciliophora | Spirotrichea | Choreotrichida-Tintinnina | Tintinnidae | *Tintinnidae_X* | *Tintinnidae_X_sp.* | Omnivore |
| 76439 | TSAR | Alveolata | Ciliophora | Spirotrichea | Euplotia | Euplotidae | *Euplotes* | *Euplotes_enigma* | Omnivore |
| 1459 | TSAR | Alveolata | Ciliophora | Spirotrichea | Euplotia | Euplotidae | *Euplotes* | *Euplotes_euryhalinus* | Omnivore |
| 1471 | TSAR | Alveolata | Ciliophora | Spirotrichea | Euplotia | Euplotidae | *Euplotes* | *Euplotes_orientalis* | Omnivore |
| 1481 | TSAR | Alveolata | Ciliophora | Spirotrichea | Euplotia | Euplotidae | *Euplotes* | *Euplotes_sp.* | Omnivore |
| 1512 | TSAR | Alveolata | Ciliophora | Spirotrichea | Hypotrichia | Halteriidae | *Halteria* | *Halteria_grandinella* | Omnivore |
| 1513 | TSAR | Alveolata | Ciliophora | Spirotrichea | Hypotrichia | Halteriidae | *Halteriidae_X* | *Halteriidae_X_sp.* | Omnivore |
| 1518 | TSAR | Alveolata | Ciliophora | Spirotrichea | Hypotrichia | Holostichidae | *Anteholosticha* | *Anteholosticha_monilata* | Omnivore |
| 1522 | TSAR | Alveolata | Ciliophora | Spirotrichea | Hypotrichia | Holostichidae | *Anteholosticha* | *Anteholosticha_sp.* | Omnivore |
| 1527 | TSAR | Alveolata | Ciliophora | Spirotrichea | Hypotrichia | Holostichidae | *Holosticha* | *Holosticha_multistylata* | Omnivore |
| 1544 | TSAR | Alveolata | Ciliophora | Spirotrichea | Hypotrichia | Hypotrichia_X | *Hypotrichia_XX* | *Hypotrichia_XX_sp.* | Omnivore |
| 1553 | TSAR | Alveolata | Ciliophora | Spirotrichea | Hypotrichia | Hypotrichia_X | *Perisincirra* | *Perisincirra_paucicirrata* | Omnivore |
| 1566 | TSAR | Alveolata | Ciliophora | Spirotrichea | Hypotrichia | Oxytrichidae | *Amphisiella* | *Amphisiella_magnigranulosa* | Omnivore |
| 1569 | TSAR | Alveolata | Ciliophora | Spirotrichea | Hypotrichia | Oxytrichidae | *Bistichella* | *Bistichella_variabilis* | Omnivore |
| 1570 | TSAR | Alveolata | Ciliophora | Spirotrichea | Hypotrichia | Oxytrichidae | *Cyrtohymena* | *Cyrtohymena_citrina* | Omnivore |
| 1574 | TSAR | Alveolata | Ciliophora | Spirotrichea | Hypotrichia | Oxytrichidae | *Gastrostyla* | *Gastrostyla_steinii* | Omnivore |
| 1575 | TSAR | Alveolata | Ciliophora | Spirotrichea | Hypotrichia | Oxytrichidae | *Gonostomum* | *Gonostomum_namibiense* | Omnivore |
| 1576 | TSAR | Alveolata | Ciliophora | Spirotrichea | Hypotrichia | Oxytrichidae | *Gonostomum* | *Gonostomum_sp.* | Omnivore |
| 1577 | TSAR | Alveolata | Ciliophora | Spirotrichea | Hypotrichia | Oxytrichidae | *Gonostomum* | *Gonostomum_strenuum* | Omnivore |
| 1579 | TSAR | Alveolata | Ciliophora | Spirotrichea | Hypotrichia | Oxytrichidae | *Hemiurosoma* | *Hemiurosoma_terricola* | Omnivore |
| 1581 | TSAR | Alveolata | Ciliophora | Spirotrichea | Hypotrichia | Oxytrichidae | *Kahliella* | *Kahliella_sp.* | Omnivore |
| 1584 | TSAR | Alveolata | Ciliophora | Spirotrichea | Hypotrichia | Oxytrichidae | *Onychodromopsis* | *Onychodromopsis_flexilis* | Omnivore |
| 1585 | TSAR | Alveolata | Ciliophora | Spirotrichea | Hypotrichia | Oxytrichidae | *Orthamphisiella* | *Orthamphisiella_breviseries* | Omnivore |
| 1586 | TSAR | Alveolata | Ciliophora | Spirotrichea | Hypotrichia | Oxytrichidae | *Orthoamphisiella* | *Orthoamphisiella_sp.* | Omnivore |
| 1589 | TSAR | Alveolata | Ciliophora | Spirotrichea | Hypotrichia | Oxytrichidae | *Oxytricha* | *Oxytricha_granulifera* | Omnivore |
| 1591 | TSAR | Alveolata | Ciliophora | Spirotrichea | Hypotrichia | Oxytrichidae | *Oxytricha* | *Oxytricha_longa* | Omnivore |
| 1592 | TSAR | Alveolata | Ciliophora | Spirotrichea | Hypotrichia | Oxytrichidae | *Oxytricha* | *Oxytricha_longigranulosa* | Omnivore |
| 1550 | TSAR | Alveolata | Ciliophora | Spirotrichea | Hypotrichia | Oxytrichidae | *Oxytricha* | *Oxytricha_sp.* | Omnivore |
| 1597 | TSAR | Alveolata | Ciliophora | Spirotrichea | Hypotrichia | Oxytrichidae | *Oxytrichidae_X* | *Oxytrichidae_X_sp.* | Omnivore |
| 1612 | TSAR | Alveolata | Ciliophora | Spirotrichea | Hypotrichia | Oxytrichidae | *Sterkiella* | *Sterkiella_histriomuscorum* | Omnivore |
| 1614 | TSAR | Alveolata | Ciliophora | Spirotrichea | Hypotrichia | Oxytrichidae | *Sterkiella* | *Sterkiella_sp.* | Omnivore |
| 1562 | TSAR | Alveolata | Ciliophora | Spirotrichea | Hypotrichia | Oxytrichidae | *Stichotrichia* | *Stichotrichia_sp.* | Omnivore |
| 1618 | TSAR | Alveolata | Ciliophora | Spirotrichea | Hypotrichia | Oxytrichidae | *Stylonychia* | *Stylonychia_lemnae* | Omnivore |
| 75470 | TSAR | Alveolata | Ciliophora | Spirotrichea | Hypotrichia | Pseudokeronopsidae | *Eschaneustyla* | *Eschaneustyla_lugeri* | Omnivore |
| 1536 | TSAR | Alveolata | Ciliophora | Spirotrichea | Hypotrichia | Pseudokeronopsidae | *Pseudokeronopsis* | *Pseudokeronopsis_sp.* | Omnivore |
| 1646 | TSAR | Alveolata | Ciliophora | Spirotrichea | Hypotrichia | Spirofilidae | *Pseudouroleptus* | *Pseudouroleptus_caudatus* | Omnivore |
| 1648 | TSAR | Alveolata | Ciliophora | Spirotrichea | Hypotrichia | Spirofilidae | *Pseudouroleptus* | *Pseudouroleptus_sp.* | Omnivore |
| 75475 | TSAR | Alveolata | Ciliophora | Spirotrichea | Hypotrichia | Strongylidiidae | *Hemiamphisiella* | *Hemiamphisiella_terricola* | Omnivore |
| 1656 | TSAR | Alveolata | Ciliophora | Spirotrichea | Hypotrichia | Urostylidae | *Paruroleptus* | *Paruroleptus_lepisma* | Omnivore |
| 75477 | TSAR | Alveolata | Ciliophora | Spirotrichea | Hypotrichia | Urostylidae | *Pseudonotohymena* | *Pseudonotohymena_antarctica* | Omnivore |
| 1660 | TSAR | Alveolata | Ciliophora | Spirotrichea | Hypotrichia | Urostylidae | *Uroleptus* | *Uroleptus_gallina* | Omnivore |
| 1661 | TSAR | Alveolata | Ciliophora | Spirotrichea | Hypotrichia | Urostylidae | *Uroleptus* | *Uroleptus_pisces* | Omnivore |
| 1662 | TSAR | Alveolata | Ciliophora | Spirotrichea | Hypotrichia | Urostylidae | *Uroleptus* | *Uroleptus_sp.* | Omnivore |
| 1663 | TSAR | Alveolata | Ciliophora | Spirotrichea | Hypotrichia | Urostylidae | *Urostyla* | *Urostyla_grandis* | Omnivore |
| 77905 | TSAR | Alveolata | Ciliophora | Spirotrichea | Hypotrichia | Urostylidae | *Urostyla* | *Urostyla_sp.* | Omnivore |
| 1664 | TSAR | Alveolata | Ciliophora | Spirotrichea | Licnophoria | Licnophoridae | *Licnophora* | *Licnophora_lyngbycola* | Omnivore |
| 74222 | TSAR | Alveolata | Ciliophora | Spirotrichea | Oligotrichida | Oligotrichida_X | *Oligotrichida_XX* | *Oligotrichida_XX_sp.* | Omnivore |
| 48728 | TSAR | Alveolata | Ciliophora | Spirotrichea | Oligotrichida | Strombidiidae | *Strombidiidae_X* | *Strombidiidae_X_sp.* | Omnivore |
| 48776 | TSAR | Alveolata | Ciliophora | Spirotrichea | Oligotrichida | Strombidiidae | *Strombidium* | *Strombidium_biarmatum* | Omnivore |
| 48777 | TSAR | Alveolata | Ciliophora | Spirotrichea | Oligotrichida | Strombidiidae | *Strombidium* | *Strombidium_paracapitatum* | Omnivore |
| 48731 | TSAR | Alveolata | Ciliophora | Spirotrichea | Oligotrichida | Strombidiidae | *Strombidium* | *Strombidium_sp.* | Omnivore |
| 1732 | TSAR | Alveolata | Ciliophora | Spirotrichea | Spirotrichea_X | Spirotrichea_XX | *Phacodinium* | *Phacodinium_metchnikoffi* | NA |
| 1765 | TSAR | Alveolata | Dinoflagellata | Dinophyceae | Dinophyceae_X | Dinophyceae_XX | *Dinophyceae_XXX* | *Dinophyceae_XXX_sp.* | NA |
| 2202 | TSAR | Alveolata | Dinoflagellata | Dinophyceae | Dinophysiales | Oxyphysiaceae | *Phalachroma* | *Phalacroma_oxytoxoides* | Mixotrophic |
| 74250 | TSAR | Alveolata | Dinoflagellata | Dinophyceae | Gonyaulacales | Pyrocystaceae | *Alexandrium* | *Alexandrium_catenella* | Mixotrophic |
| 1749 | TSAR | Alveolata | Dinoflagellata | Dinophyceae | Gonyaulacales | Pyrocystaceae | *Alexandrium* | *Alexandrium_margalefii* | Mixotrophic |
| 48959 | TSAR | Alveolata | Dinoflagellata | Dinophyceae | Gonyaulacales | Pyrocystaceae | *Alexandrium* | *Alexandrium_pacificum* | Mixotrophic |
| 2153 | TSAR | Alveolata | Dinoflagellata | Dinophyceae | Gonyaulacales | Pyrocystaceae | *Coolia* | *Coolia_malayensis* | Mixotrophic |
| 1892 | TSAR | Alveolata | Dinoflagellata | Dinophyceae | Gonyaulacales | Pyrocystaceae | *Gambierdiscus* | *Gambierdiscus_belizeanus* | Mixotrophic |
| 1893 | TSAR | Alveolata | Dinoflagellata | Dinophyceae | Gonyaulacales | Pyrocystaceae | *Gambierdiscus* | *Gambierdiscus_caribaeus* | Mixotrophic |
| 74254 | TSAR | Alveolata | Dinoflagellata | Dinophyceae | Gonyaulacales | Pyrocystaceae | *Gambierdiscus* | *Gambierdiscus_honu* | Mixotrophic |
| 1895 | TSAR | Alveolata | Dinoflagellata | Dinophyceae | Gonyaulacales | Pyrocystaceae | *Gambierdiscus* | *Gambierdiscus_pacificus* | Mixotrophic |
| 49003 | TSAR | Alveolata | Dinoflagellata | Dinophyceae | Gonyaulacales | Pyrocystaceae | *Gambierdiscus* | *Gambierdiscus_scabrosus* | Mixotrophic |
| 1898 | TSAR | Alveolata | Dinoflagellata | Dinophyceae | Gonyaulacales | Pyrocystaceae | *Gambierdiscus* | *Gambierdiscus_sp.* | Mixotrophic |
| 2157 | TSAR | Alveolata | Dinoflagellata | Dinophyceae | Gonyaulacales | Pyrocystaceae | *Ostreopsis* | *Ostreopsis_ovata* | Mixotrophic |
| 1829 | TSAR | Alveolata | Dinoflagellata | Dinophyceae | Gymnodiniales | Chytriodiniaceae | *Chytriodinium* | *Chytriodinium_roseum* | Animal Parasite |
| 1917 | TSAR | Alveolata | Dinoflagellata | Dinophyceae | Gymnodiniales | Gymnodiniaceae | *Gymnodinium* | *Gymnodinium_aureolum* | Mixotrophic |
| 1927 | TSAR | Alveolata | Dinoflagellata | Dinophyceae | Gymnodiniales | Gymnodiniaceae | *Gymnodinium* | *Gymnodinium_sp.* | Mixotrophic |
| 1935 | TSAR | Alveolata | Dinoflagellata | Dinophyceae | Gymnodiniales | Gymnodiniaceae | *Gyrodinium* | *Gyrodinium_fusiforme* | Mixotrophic |
| 1940 | TSAR | Alveolata | Dinoflagellata | Dinophyceae | Gymnodiniales | Gymnodiniaceae | *Gyrodinium* | *Gyrodinium_sp.* | Mixotrophic |
| 1978 | TSAR | Alveolata | Dinoflagellata | Dinophyceae | Gymnodiniales | Gymnodiniaceae | *Lepidodinium* | *Lepidodinium_sp.* | Phototrophic |
| 1979 | TSAR | Alveolata | Dinoflagellata | Dinophyceae | Gymnodiniales | Gymnodiniaceae | *Lepidodinium* | *Lepidodinium_viride* | Phototrophic |
| 49036 | TSAR | Alveolata | Dinoflagellata | Dinophyceae | Gymnodiniales | Gymnodiniaceae | *Nusuttodinium* | *Nusuttodinium_amphidinioides* | Mixotrophic |
| 49040 | TSAR | Alveolata | Dinoflagellata | Dinophyceae | Gymnodiniales | Gymnodiniaceae | *Nusuttodinium* | *Nusuttodinium_poecilochroum* | Mixotrophic |
| 2130 | TSAR | Alveolata | Dinoflagellata | Dinophyceae | Gymnodiniales | Gymnodiniaceae | *Spiniferodinium* | *Spiniferodinium_galeiforme* | Mixotrophic |
| 75531 | TSAR | Alveolata | Dinoflagellata | Dinophyceae | Gymnodiniales | Gymnodiniaceae | *Wangodinium* | *Wangodinium_sinense* | Phototrophic |
| 2217 | TSAR | Alveolata | Dinoflagellata | Dinophyceae | Gymnodiniales | Kareniaceae | *Karlodinium* | *Karlodinium_veneficum* | Mixotrophic |
| 2147 | TSAR | Alveolata | Dinoflagellata | Dinophyceae | Gymnodiniales | Warnowiaceae | *Warnowia* | *Warnowia_sp.* | Eukaryvore |
| 1948 | TSAR | Alveolata | Dinoflagellata | Dinophyceae | Peridiniales | Amphidiniopsidaceae | *Herdmania* | *Herdmania_litoralis* | Eukaryvore |
| 1949 | TSAR | Alveolata | Dinoflagellata | Dinophyceae | Peridiniales | Heterocapsaceae | *Heterocapsa* | *Heterocapsa_arctica* | Mixotrophic |
| 48663 | TSAR | Alveolata | Dinoflagellata | Dinophyceae | Peridiniales | Heterocapsaceae | *Heterocapsa* | *Heterocapsa_nei/rotundata* | Mixotrophic |
| 49016 | TSAR | Alveolata | Dinoflagellata | Dinophyceae | Peridiniales | Heterocapsaceae | *Heterocapsa* | *Heterocapsa_niei* | Mixotrophic |
| 1953 | TSAR | Alveolata | Dinoflagellata | Dinophyceae | Peridiniales | Heterocapsaceae | *Heterocapsa* | *Heterocapsa_pygmaea* | Mixotrophic |
| 49018 | TSAR | Alveolata | Dinoflagellata | Dinophyceae | Peridiniales | Heterocapsaceae | *Heterocapsa* | *Heterocapsa_rotundata* | Mixotrophic |
| 1955 | TSAR | Alveolata | Dinoflagellata | Dinophyceae | Peridiniales | Heterocapsaceae | *Heterocapsa* | *Heterocapsa_sp.* | Mixotrophic |
| 2035 | TSAR | Alveolata | Dinoflagellata | Dinophyceae | Peridiniales | Peridiniaceae | *Peridinium* | *Peridinium_sp.* | Phototrophic |
| 48986 | TSAR | Alveolata | Dinoflagellata | Dinophyceae | Peridiniales | Peridiniales_X | *Bysmatrum* | *Bysmatrum_gregarium* | Mixotrophic |
| 49046 | TSAR | Alveolata | Dinoflagellata | Dinophyceae | Peridiniales | Peridiniopsidaceae | *Parvodinium* | *Parvodinium_umbonatum* | Phototrophic |
| 48971 | TSAR | Alveolata | Dinoflagellata | Dinophyceae | Peridiniales | Thoracosphaeraceae | *Apocalathium* | *Apocalathium_aciculiferum* | Phototrophic |
| 2118 | TSAR | Alveolata | Dinoflagellata | Dinophyceae | Peridiniales | Thoracosphaeraceae | *Apocalathium* | *Apocalathium_malmogiense* | Phototrophic |
| 2166 | TSAR | Alveolata | Dinoflagellata | Dinophyceae | Peridiniales | Thoracosphaeraceae | *Luciella* | *Luciella_sp.* | Eukaryvore |
| 2167 | TSAR | Alveolata | Dinoflagellata | Dinophyceae | Peridiniales | Thoracosphaeraceae | *Paulsenella* | *Paulsenella_vonstoschii* | Eukaryvore |
| 2012 | TSAR | Alveolata | Dinoflagellata | Dinophyceae | Peridiniales | Thoracosphaeraceae | *Pentapharsodinium* | *Pentapharsodinium_tyrrhenicum* | Phototrophic |
| 77597 | TSAR | Alveolata | Dinoflagellata | Dinophyceae | Peridiniales | Thoracosphaeraceae | *Scrippsiella* | *Scrippsiella_hangoei* | Mixotrophic |
| 2121 | TSAR | Alveolata | Dinoflagellata | Dinophyceae | Peridiniales | Thoracosphaeraceae | *Scrippsiella* | *Scrippsiella_sp.* | Mixotrophic |
| 2170 | TSAR | Alveolata | Dinoflagellata | Dinophyceae | Peridiniales | Thoracosphaeraceae | *Thoracosphaeraceae_X* | *Thoracosphaeraceae_X_sp.* | NA |
| 2211 | TSAR | Alveolata | Dinoflagellata | Dinophyceae | Suessiales | Borghiellaceae | *Borghiella* | *Borghiella_tenuissima* | Phototrophic |
| 74246 | TSAR | Alveolata | Dinoflagellata | Dinophyceae | Suessiales | Suessiaceae | *Ansanella* | *Ansanella_catalana* | Phototrophic |
| 48970 | TSAR | Alveolata | Dinoflagellata | Dinophyceae | Suessiales | Suessiaceae | *Ansanella* | *Ansanella_granifera* | Phototrophic |
| 48978 | TSAR | Alveolata | Dinoflagellata | Dinophyceae | Suessiales | Suessiaceae | *Biecheleria* | *Biecheleria_baltica* | Mixotrophic |
| 48979 | TSAR | Alveolata | Dinoflagellata | Dinophyceae | Suessiales | Suessiaceae | *Biecheleria* | *Biecheleria_brevisulcata* | Mixotrophic |
| 70089 | TSAR | Alveolata | Dinoflagellata | Dinophyceae | Suessiales | Suessiaceae | *Biecheleria* | *Biecheleria_sp.* | Mixotrophic |
| 48983 | TSAR | Alveolata | Dinoflagellata | Dinophyceae | Suessiales | Suessiaceae | *Biecheleriopsis* | *Biecheleriopsis_adriatica* | Phototrophic |
| 74247 | TSAR | Alveolata | Dinoflagellata | Dinophyceae | Suessiales | Suessiaceae | *Biecheleriopsis* | *Biecheleriopsis_sp.* | Phototrophic |
| 2218 | TSAR | Alveolata | Dinoflagellata | Dinophyceae | Suessiales | Suessiaceae | *Pelagodinium* | *Pelagodinium_beii* | Phototrophic |
| 2219 | TSAR | Alveolata | Dinoflagellata | Dinophyceae | Suessiales | Suessiaceae | *Pelagodinium* | *Pelagodinium_sp.* | Phototrophic |
| 2222 | TSAR | Alveolata | Dinoflagellata | Dinophyceae | Suessiales | Suessiaceae | *Polarella* | *Polarella_glacialis* | Phototrophic |
| 70091 | TSAR | Alveolata | Dinoflagellata | Dinophyceae | Suessiales | Suessiaceae | *Suessiaceae_X* | *Suessiaceae_X_sp.* | Phototrophic |
| 2234 | TSAR | Alveolata | Dinoflagellata | Dinophyceae | Suessiales | Symbiodiniaceae | *Symbiodinium* | *Symbiodinium_sp.* | Mixotrophic |
| 2261 | TSAR | Alveolata | Dinoflagellata | Syndiniales | Dino-Group-I | Dino-Group-I-Clade-4 | *Dino-Group-I-Clade-4_X* | *Dino-Group-I-Clade-4_X_sp.* | NA |
| 2276 | TSAR | Alveolata | Dinoflagellata | Syndiniales | Dino-Group-I | Dino-Group-I-Clade-7 | *Dino-Group-I-Clade-7_X* | *Dino-Group-I-Clade-7_X_sp.* | NA |
| 2278 | TSAR | Alveolata | Dinoflagellata | Syndiniales | Dino-Group-II | Dino-Group-II_X | *Dino-Group-II_XX* | *Dino-Group-II_XX_sp.* | NA |
| 2281 | TSAR | Alveolata | Dinoflagellata | Syndiniales | Dino-Group-II | Dino-Group-II-Clade-1 | *Dino-Group-II-Clade-1_X* | *Dino-Group-II-Clade-1_X_sp.* | NA |
| 2300 | TSAR | Alveolata | Dinoflagellata | Syndiniales | Dino-Group-II | Dino-Group-II-Clade-12 | *Dino-Group-II-Clade-12_X* | *Dino-Group-II-Clade-12_X_sp.* | NA |
| 2316 | TSAR | Alveolata | Dinoflagellata | Syndiniales | Dino-Group-II | Dino-Group-II-Clade-19 | *Dino-Group-II-Clade-19_X* | *Dino-Group-II-Clade-19_X_sp.* | NA |
| 2321 | TSAR | Alveolata | Dinoflagellata | Syndiniales | Dino-Group-II | Dino-Group-II-Clade-21 | *Dino-Group-II-Clade-21_X* | *Dino-Group-II-Clade-21_X_sp.* | NA |
| 2327 | TSAR | Alveolata | Dinoflagellata | Syndiniales | Dino-Group-II | Dino-Group-II-Clade-24 | *Dino-Group-II-Clade-24_X* | *Dino-Group-II-Clade-24_X_sp.* | NA |
| 2355 | TSAR | Alveolata | Dinoflagellata | Syndiniales | Dino-Group-II | Dino-Group-II-Clade-4 | *Amoebophrya* | *Amoebophrya_ceratii* | Dinoflagellate Parasite |
| 2357 | TSAR | Alveolata | Dinoflagellata | Syndiniales | Dino-Group-II | Dino-Group-II-Clade-4 | *Dino-Group-II-Clade-4_X* | *Dino-Group-II-Clade-4_X_sp.* | NA |
| 2364 | TSAR | Alveolata | Dinoflagellata | Syndiniales | Dino-Group-II | Dino-Group-II-Clade-5 | *Dino-Group-II-Clade-5_X* | *Dino-Group-II-Clade-5_X_sp.* | NA |
| 2386 | TSAR | Alveolata | Dinoflagellata | Syndiniales | Dino-Group-IV | Dino-Group-IV-Hematodinium-Group | *Hematodinium* | *Hematodinium_sp.* | Animal Parasite |
| 75559 | TSAR | Alveolata | Perkinsea | Perkinsida | Perkinsida_X | Parviluciferaceae | *Dinovorax* | *Dinovorax_pyriformis* | Dinoflagellate Parasite |
| 2397 | TSAR | Alveolata | Perkinsea | Perkinsida | Perkinsida_X | Perkinsida_XX | *Perkinsida_XXX* | *Perkinsida_XXX_sp.* | Dinoflagellate Parasite |
| 45040 | TSAR | Rhizaria | Cercozoa | Cercozoa_X | Cercozoa_XX | Cercozoa_XXX | *Cercozoa_XXXX* | *Cercozoa_XXXX_sp.* | Heterotrophic |
| 75565 | TSAR | Rhizaria | Cercozoa | Cercozoa_X | Cercozoa_XX | Cercozoa_XXX | *Kraken* | *Kraken_carinae* | Bacterivore |
| 45061 | TSAR | Rhizaria | Cercozoa | Endomyxa | Vampyrellida | Leptophryidae | *Arachnula* | *Arachnula_impatiens* | Eukaryvore |
| 45062 | TSAR | Rhizaria | Cercozoa | Endomyxa | Vampyrellida | Leptophryidae | *Leptophryidae_X* | *Leptophryidae_X_sp.* | Eukaryvore |
| 45066 | TSAR | Rhizaria | Cercozoa | Endomyxa | Vampyrellida | Leptophryidae | *Leptophrys* | *Leptophrys_vorax* | Eukaryvore |
| 45067 | TSAR | Rhizaria | Cercozoa | Endomyxa | Vampyrellida | Leptophryidae | *Platyreta* | *Platyreta_germanica* | Eukaryvore |
| 75606 | TSAR | Rhizaria | Cercozoa | Endomyxa | Vampyrellida | Leptophryidae | *Vernalophrys* | *Vernalophrys_algivore* | Eukaryvore |
| 45069 | TSAR | Rhizaria | Cercozoa | Endomyxa | Vampyrellida | op14-lineage | *op14-lineage_X* | *op14-lineage_X_sp.* | Eukaryvore |
| 45070 | TSAR | Rhizaria | Cercozoa | Endomyxa | Vampyrellida | Penardia-lineage | *Penardia-lineage_X* | *Penardia-lineage_X_sp.* | Eukaryvore |
| 45071 | TSAR | Rhizaria | Cercozoa | Endomyxa | Vampyrellida | Penardia-lineage | *Vampyrellida_clade_C* | *Penardia_sp.* | Eukaryvore |
| 45072 | TSAR | Rhizaria | Cercozoa | Endomyxa | Vampyrellida | Sey055-lineage | *Sey055-lineage_X* | *Sey055-lineage_X_sp.* | Eukaryvore |
| 45073 | TSAR | Rhizaria | Cercozoa | Endomyxa | Vampyrellida | sm27-lineage | *sm27-lineage_X* | *sm27-lineage_X_sp.* | Eukaryvore |
| 45075 | TSAR | Rhizaria | Cercozoa | Endomyxa | Vampyrellida | Thalassomyxa-lineage | *Thalassomyxa* | *Thalassomyxa_sp.* | Eukaryvore |
| 45074 | TSAR | Rhizaria | Cercozoa | Endomyxa | Vampyrellida | Thalassomyxa-lineage | *Thalassomyxa-lineage_X* | *Thalassomyxa-lineage_X_sp.* | Eukaryvore |
| 45076 | TSAR | Rhizaria | Cercozoa | Endomyxa | Vampyrellida | Vampyrellida_X | *Vampyrellida_clade_B* | *Vampyrellida_sp.* | Eukaryvore |
| 45079 | TSAR | Rhizaria | Cercozoa | Endomyxa | Vampyrellida | Vampyrellidae | *Vampyrella* | *Vampyrella_pendula* | Eukaryvore |
| 45080 | TSAR | Rhizaria | Cercozoa | Endomyxa | Vampyrellida | Vampyrellidae | *Vampyrella* | *Vampyrella_sp.* | Eukaryvore |
| 45130 | TSAR | Rhizaria | Cercozoa | Endomyxa-Phytomyxea | Plasmodiophorida | Plasmodiophorida_X | *Spongospora* | *Spongospora_nasturtii* | Plant Parasite |
| 45131 | TSAR | Rhizaria | Cercozoa | Endomyxa-Phytomyxea | Plasmodiophorida | Plasmodiophorida_X | *Spongospora* | *Spongospora_sp.* | Plant Parasite |
| 45161 | TSAR | Rhizaria | Cercozoa | Filosa-Granofilosea | Cryptofilida | Mesofilidae | *Mesofila* | *Mesofila_limnetica* | Bacterivore |
| 45184 | TSAR | Rhizaria | Cercozoa | Filosa-Granofilosea | Filosa-Granofilosea_X | Novel-Gran-5 | *Novel-Gran-5_X* | *Novel-Gran-5_X_sp.* | Heterotrophic |
| 45185 | TSAR | Rhizaria | Cercozoa | Filosa-Granofilosea | Filosa-Granofilosea_X | Novel-Gran-6 | *Novel-Gran-6_X* | *Novel-Gran-6_X_sp.* | Heterotrophic |
| 45191 | TSAR | Rhizaria | Cercozoa | Filosa-Granofilosea | Limnofilida | Limnofilidae | *Limnofilidae_X* | *Limnofilidae_X_sp.* | Bacterivore |
| 45203 | TSAR | Rhizaria | Cercozoa | Filosa-Imbricatea | Euglyphida | Euglyphida_X | *Euglyphida_XX* | *Euglyphida_XX_sp.* | Omnivore |
| 45206 | TSAR | Rhizaria | Cercozoa | Filosa-Imbricatea | Euglyphida | Euglyphidae | *Assulina* | *Assulina_muscorum* | Omnivore |
| 45210 | TSAR | Rhizaria | Cercozoa | Filosa-Imbricatea | Euglyphida | Euglyphidae | *Euglypha* | *Euglypha_compressa* | Omnivore |
| 45212 | TSAR | Rhizaria | Cercozoa | Filosa-Imbricatea | Euglyphida | Euglyphidae | *Euglypha* | *Euglypha_penardi* | Omnivore |
| 45213 | TSAR | Rhizaria | Cercozoa | Filosa-Imbricatea | Euglyphida | Euglyphidae | *Euglypha* | *Euglypha_rotunda* | Omnivore |
| 45227 | TSAR | Rhizaria | Cercozoa | Filosa-Imbricatea | Euglyphida | Paulinellidae | *Paulinella* | *Paulinella_chromatophora* | Omnivore |
| 45233 | TSAR | Rhizaria | Cercozoa | Filosa-Imbricatea | Euglyphida | Trinematidae | *Trinema* | *Trinema_lineare* | Omnivore |
| 45237 | TSAR | Rhizaria | Cercozoa | Filosa-Imbricatea | Filosa-Imbricatea_X | Novel-clade-2 | *Novel-clade-2_X* | *Novel-clade-2_X_sp.* | Heterotrophic |
| 45249 | TSAR | Rhizaria | Cercozoa | Filosa-Imbricatea | Marimonadida | Marimonadida_X | *Marimonadida_XX* | *Marimonadida_XX_sp.* | Heterotrophic |
| 45256 | TSAR | Rhizaria | Cercozoa | Filosa-Imbricatea | Spongomonadida | Spongomonadidae | *Spongomonas* | *Spongomonas_minima* | Bacterivore |
| 45261 | TSAR | Rhizaria | Cercozoa | Filosa-Imbricatea | Thaumatomonadida | Peregriniidae | *Peregrinia* | *Peregrinia_sp.* | Bacterivore |
| 45262 | TSAR | Rhizaria | Cercozoa | Filosa-Imbricatea | Thaumatomonadida | Thaumatomonadida_X | *Thaumatomonadida_XX* | *Thaumatomonadida_XX_sp.* | Heterotrophic |
| 45265 | TSAR | Rhizaria | Cercozoa | Filosa-Imbricatea | Thaumatomonadida | Thaumatomonadidae | *Reckertia* | *Reckertia_filosa* | Bacterivore |
| 45273 | TSAR | Rhizaria | Cercozoa | Filosa-Imbricatea | Thaumatomonadida | Thaumatomonadidae | *Thaumatomonas* | *Thaumatomonas_sp.* | Bacterivore |
| 45309 | TSAR | Rhizaria | Cercozoa | Filosa-Sarcomonadea | Cercomonadida | Cercomonadidae | *Cercomonas* | *Cercomonas_plasmodialis* | Omnivore |
| 45314 | TSAR | Rhizaria | Cercozoa | Filosa-Sarcomonadea | Cercomonadida | Cercomonadidae | *Cercomonas* | *Cercomonas_sp.* | Omnivore |
| 45322 | TSAR | Rhizaria | Cercozoa | Filosa-Sarcomonadea | Cercomonadida | Cercomonadidae | *Eocercomonas* | *Eocercomonas_sp.* | Omnivore |
| 75562 | TSAR | Rhizaria | Cercozoa | Filosa-Sarcomonadea | Cercomonadida | Cercomonadidae | *Neocercomonas* | *Neocercomonas_nitschei* | Omnivore |
| 76997 | TSAR | Rhizaria | Cercozoa | Filosa-Sarcomonadea | Cercomonadida | Cercomonadidae | *Neocercomonas* | *Neocercomonas_sp.* | Omnivore |
| 75563 | TSAR | Rhizaria | Cercozoa | Filosa-Sarcomonadea | Cercomonadida | Cercomonadidae | *Neocercomonas* | *Neocercomonas_tuberculata* | Omnivore |
| 45327 | TSAR | Rhizaria | Cercozoa | Filosa-Sarcomonadea | Cercomonadida | Paracercomonadidae | *Metabolomonas* | *Metabolomonas_insania* | Bacterivore |
| 45328 | TSAR | Rhizaria | Cercozoa | Filosa-Sarcomonadea | Cercomonadida | Paracercomonadidae | *Nucleocercomonas* | *Nucleocercomonas_praelonga* | Bacterivore |
| 45347 | TSAR | Rhizaria | Cercozoa | Filosa-Sarcomonadea | Cercomonadida | Paracercomonadidae | *Paracercomonas* | *Paracercomonas_sp.* | Bacterivore |
| 45355 | TSAR | Rhizaria | Cercozoa | Filosa-Sarcomonadea | Glissomonadida | Allapsidae | *Allantion* | *Allantion_sp.* | Bacterivore |
| 45360 | TSAR | Rhizaria | Cercozoa | Filosa-Sarcomonadea | Glissomonadida | Allapsidae | *Allapsidae_X* | *Allapsidae_X_sp.* | Bacterivore |
| 45361 | TSAR | Rhizaria | Cercozoa | Filosa-Sarcomonadea | Glissomonadida | Allapsidae | *Group-Te* | *Group_Te_sp.* | Bacterivore |
| 45363 | TSAR | Rhizaria | Cercozoa | Filosa-Sarcomonadea | Glissomonadida | Allapsidae | *Orciraptor* | *Orciraptor_agilis* | Bacterivore |
| 45366 | TSAR | Rhizaria | Cercozoa | Filosa-Sarcomonadea | Glissomonadida | Allapsidae | *Viridiraptor* | *Viridiraptor_invadens* | Eukaryvore |
| 45370 | TSAR | Rhizaria | Cercozoa | Filosa-Sarcomonadea | Glissomonadida | Bodomorphidae | *Bodomorpha* | *Bodomorpha_sp.* | Bacterivore |
| 45371 | TSAR | Rhizaria | Cercozoa | Filosa-Sarcomonadea | Glissomonadida | Glissomonadida_Clade-Y | *Glissomonadida_Clade-Y_X* | *Glissomonadida_Clade-Y_X_sp.* | Heterotrophic |
| 45374 | TSAR | Rhizaria | Cercozoa | Filosa-Sarcomonadea | Glissomonadida | Glissomonadida_X | *Glissomonadida_XX* | *Glissomonadida_XX_sp.* | Heterotrophic |
| 45379 | TSAR | Rhizaria | Cercozoa | Filosa-Sarcomonadea | Glissomonadida | Sandonidae | *Flectomonas* | *Flectomonas_ekelundi* | Bacterivore |
| 45386 | TSAR | Rhizaria | Cercozoa | Filosa-Sarcomonadea | Glissomonadida | Sandonidae | *Sandona* | *Sandona_aporians* | Bacterivore |
| 77572 | TSAR | Rhizaria | Cercozoa | Filosa-Sarcomonadea | Glissomonadida | Sandonidae | *Sandona* | *Sandona_mutans* | Bacterivore |
| 45390 | TSAR | Rhizaria | Cercozoa | Filosa-Sarcomonadea | Glissomonadida | Sandonidae | *Sandona* | *Sandona_sp.* | Bacterivore |
| 45391 | TSAR | Rhizaria | Cercozoa | Filosa-Sarcomonadea | Glissomonadida | Sandonidae | *Sandonidae_X* | *Sandonidae_X_sp.* | Bacterivore |
| 45401 | TSAR | Rhizaria | Cercozoa | Filosa-Sarcomonadea | Pansomonadida | Pansomonadida_X | *Aurigamonas* | *Aurigamonas_solis* | Omnivore |
| 45402 | TSAR | Rhizaria | Cercozoa | Filosa-Sarcomonadea | Pansomonadida | Pansomonadida_X | *Pansomonadida_XX* | *Pansomonadida_XX_sp.* | Heterotrophic |
| 45405 | TSAR | Rhizaria | Cercozoa | Filosa-Thecofilosea | Cryomonadida | Cryothecomonas-lineage | *Cryothecomonas* | *Cryothecomonas_aestivalis* | Eukaryvore |
| 45408 | TSAR | Rhizaria | Cercozoa | Filosa-Thecofilosea | Cryomonadida | Cryothecomonas-lineage | *Cryothecomonas* | *Cryothecomonas_sp.* | Eukaryvore |
| 45409 | TSAR | Rhizaria | Cercozoa | Filosa-Thecofilosea | Cryomonadida | Cryothecomonas-lineage | *Cryothecomonas-lineage_X* | *Cryothecomonas-lineage_X_sp.* | Eukaryvore |
| 45410 | TSAR | Rhizaria | Cercozoa | Filosa-Thecofilosea | Cryomonadida | Protaspa-lineage | *Protaspa* | *Protaspa_grandis* | Eukaryvore |
| 45411 | TSAR | Rhizaria | Cercozoa | Filosa-Thecofilosea | Cryomonadida | Protaspa-lineage | *Protaspa* | *Protaspa_longipes* | Eukaryvore |
| 45413 | TSAR | Rhizaria | Cercozoa | Filosa-Thecofilosea | Cryomonadida | Protaspa-lineage | *Protaspa* | *Protaspa_sp.* | Eukaryvore |
| 45414 | TSAR | Rhizaria | Cercozoa | Filosa-Thecofilosea | Cryomonadida | Protaspa-lineage | *Protaspa-lineage_X* | *Protaspa-lineage_X_sp.* | Eukaryvore |
| 77539 | TSAR | Rhizaria | Cercozoa | Filosa-Thecofilosea | Cryomonadida | Rhogostoma-lineage | *Rhogostoma* | *Rhogostoma_bowseri* | Omnivore |
| 75590 | TSAR | Rhizaria | Cercozoa | Filosa-Thecofilosea | Cryomonadida | Rhogostoma-lineage | *Rhogostoma* | *Rhogostoma_cylindrica* | Omnivore |
| 77543 | TSAR | Rhizaria | Cercozoa | Filosa-Thecofilosea | Cryomonadida | Rhogostoma-lineage | *Rhogostoma* | *Rhogostoma_leviosa* | Omnivore |
| 77545 | TSAR | Rhizaria | Cercozoa | Filosa-Thecofilosea | Cryomonadida | Rhogostoma-lineage | *Rhogostoma* | *Rhogostoma_pseudocylindrica* | Omnivore |
| 45431 | TSAR | Rhizaria | Cercozoa | Filosa-Thecofilosea | Cryomonadida | Rhogostoma-lineage | *Rhogostoma-lineage_X* | *Rhogostoma-lineage_X_sp.* | Omnivore |
| 45437 | TSAR | Rhizaria | Cercozoa | Filosa-Thecofilosea | Ebriacea | TAGIRI1-lineage | *TAGIRI1-lineage_X* | *TAGIRI1-lineage_X_sp.* | Heterotrophic |
| 45438 | TSAR | Rhizaria | Cercozoa | Filosa-Thecofilosea | Filosa-Thecofilosea_X | Filosa-Thecofilosea_XX | *Filosa-Thecofilosea_XXX* | *Filosa-Thecofilosea_XXX_sp.* | Heterotrophic |
| 45442 | TSAR | Rhizaria | Cercozoa | Filosa-Thecofilosea | Filosa-Thecofilosea_X | Mataza-lineage | *Mataza-lineage_X* | *Mataza-lineage_X_sp.* | Heterotrophic |
| 45451 | TSAR | Rhizaria | Cercozoa | Filosa-Thecofilosea | Ventricleftida | CCW10-lineage | *CCW10-lineage_X* | *CCW10-lineage_X_sp.* | Eukaryvore |
| 45452 | TSAR | Rhizaria | Cercozoa | Filosa-Thecofilosea | Ventricleftida | Ventricleftida_X | *Ventricleftida_XX* | *Ventricleftida_XX_sp.* | Eukaryvore |
| 75600 | TSAR | Rhizaria | Cercozoa | Filosa-Thecofilosea | Ventricleftida | Ventrifissuridae | *Ventrifissura* | *Ventrifissura_oblonga* | Eukaryvore |
| 75601 | TSAR | Rhizaria | Cercozoa | Filosa-Thecofilosea | Ventricleftida | Ventrifissuridae | *Ventrifissura* | *Ventrifissura_velata* | Eukaryvore |
| 45460 | TSAR | Rhizaria | Cercozoa | Novel-clade-10-12 | Novel-clade-10 | Novel-clade-10_X | *Novel-clade-10_XX* | *Novel-clade-10_XX_sp.* | Eukaryvore |
| 45463 | TSAR | Rhizaria | Cercozoa | Novel-clade-10-12 | Novel-clade-12 | NC12B-lineage | *NC12B-lineage_X* | *NC12B-lineage_X_sp.* | Heterotrophic |
| 45535 | TSAR | Rhizaria | Foraminifera | Globothalamea | Rotaliida | Cassidulinidae | *Glandulina* | *Glandulina_antarctica* | Bacterivore |
| 45722 | TSAR | Rhizaria | Foraminifera | Monothalamids | Monothalamids_X | Lagynidae | *Nemogullmia* | *Nemogullmia_sp.* | Bacterivore |
| 70205 | TSAR | Rhizaria | Radiolaria | Acantharea | Acantharea_1 | Acantharea_1_X | *Acantharea_1_XX* | *Acantharea_1_XX_sp.* | Mixotrophic |
| 70418 | TSAR | Rhizaria | Radiolaria | RAD-A | RAD-A_X | RAD-A_Xg | *RAD-A_Xg_X* | *RAD-A_Xg_X_sp.* | Mixotrophic |
| 46476 | TSAR | Stramenopiles | Bacillariophyceae | Bacillariophyceae | Achnanthales | Achnanthidiaceae | *Achnanthidium* | *Achnanthidium_minutissimum* | Phototrophic |
| 46512 | TSAR | Stramenopiles | Bacillariophyceae | Bacillariophyceae | Bacillariales | Bacillariaceae | *Bacillaria* | *Bacillaria_paxillifer* | Phototrophic |
| 46544 | TSAR | Stramenopiles | Bacillariophyceae | Bacillariophyceae | Bacillariales | Bacillariaceae | *Cylindrotheca* | *Cylindrotheca_closterium* | Phototrophic |
| 46545 | TSAR | Stramenopiles | Bacillariophyceae | Bacillariophyceae | Bacillariales | Bacillariaceae | *Cylindrotheca* | *Cylindrotheca_fusiformis* | Phototrophic |
| 46642 | TSAR | Stramenopiles | Bacillariophyceae | Bacillariophyceae | Bacillariales | Bacillariaceae | *Denticula* | *Denticula_kuetzingii* | Phototrophic |
| 46678 | TSAR | Stramenopiles | Bacillariophyceae | Bacillariophyceae | Bacillariales | Bacillariaceae | *Fragilariopsis* | *Fragilariopsis_cylindrus* | Phototrophic |
| 46716 | TSAR | Stramenopiles | Bacillariophyceae | Bacillariophyceae | Bacillariales | Bacillariaceae | *Hantzschia* | *Hantzschia_amphioxys* | Phototrophic |
| 46767 | TSAR | Stramenopiles | Bacillariophyceae | Bacillariophyceae | Bacillariales | Bacillariaceae | *Nitzschia* | *Nitzschia_acidoclinata* | Phototrophic |
| 77021 | TSAR | Stramenopiles | Bacillariophyceae | Bacillariophyceae | Bacillariales | Bacillariaceae | *Nitzschia* | *Nitzschia_aequorea* | Phototrophic |
| 46768 | TSAR | Stramenopiles | Bacillariophyceae | Bacillariophyceae | Bacillariales | Bacillariaceae | *Nitzschia* | *Nitzschia_amphibia* | Phototrophic |
| 46771 | TSAR | Stramenopiles | Bacillariophyceae | Bacillariophyceae | Bacillariales | Bacillariaceae | *Nitzschia* | *Nitzschia_clade_epithemoides/thermalis* | Phototrophic |
| 46779 | TSAR | Stramenopiles | Bacillariophyceae | Bacillariophyceae | Bacillariales | Bacillariaceae | *Nitzschia* | *Nitzschia_fonticola* | Phototrophic |
| 46781 | TSAR | Stramenopiles | Bacillariophyceae | Bacillariophyceae | Bacillariales | Bacillariaceae | *Nitzschia* | *Nitzschia_frustulum* | Phototrophic |
| 46782 | TSAR | Stramenopiles | Bacillariophyceae | Bacillariophyceae | Bacillariales | Bacillariaceae | *Nitzschia* | *Nitzschia_inconspicua* | Phototrophic |
| 46793 | TSAR | Stramenopiles | Bacillariophyceae | Bacillariophyceae | Bacillariales | Bacillariaceae | *Nitzschia* | *Nitzschia_sp.* | Phototrophic |
| 46798 | TSAR | Stramenopiles | Bacillariophyceae | Bacillariophyceae | Bacillariales | Bacillariaceae | *Nitzschia* | *Nitzschia_thermalis* | Phototrophic |
| 46861 | TSAR | Stramenopiles | Bacillariophyceae | Bacillariophyceae | Bacillariales | Bacillariaceae | *Pseudo-nitzschia* | *Pseudo-nitzschia_lineola* | Phototrophic |
| 46864 | TSAR | Stramenopiles | Bacillariophyceae | Bacillariophyceae | Bacillariales | Bacillariaceae | *Pseudo-nitzschia* | *Pseudo-nitzschia_pungens* | Phototrophic |
| 46865 | TSAR | Stramenopiles | Bacillariophyceae | Bacillariophyceae | Bacillariales | Bacillariaceae | *Pseudo-nitzschia* | *Pseudo-nitzschia_seriata* | Phototrophic |
| 46866 | TSAR | Stramenopiles | Bacillariophyceae | Bacillariophyceae | Bacillariales | Bacillariaceae | *Pseudo-nitzschia* | *Pseudo-nitzschia_sp.* | Phototrophic |
| 70131 | TSAR | Stramenopiles | Bacillariophyceae | Bacillariophyceae | Bacillariales | Bacillariaceae | *Pseudo-nitzschia* | *Pseudo-nitzschia_subcurvata* | Phototrophic |
| 46513 | TSAR | Stramenopiles | Bacillariophyceae | Bacillariophyceae | Bacillariophyceae_X | Bacillariophyceae_XX | *Bacillariophyceae_XXX* | *Bacillariophyceae_XXX_sp.* | Phototrophic |
| 46555 | TSAR | Stramenopiles | Bacillariophyceae | Bacillariophyceae | Cymbellales | Cymbellaceae | *Cymbella* | *Cymbella_affinis* | Phototrophic |
| 76269 | TSAR | Stramenopiles | Bacillariophyceae | Bacillariophyceae | Cymbellales | Cymbellaceae | *Cymbella* | *Cymbella_cistula* | Phototrophic |
| 46577 | TSAR | Stramenopiles | Bacillariophyceae | Bacillariophyceae | Cymbellales | Cymbellaceae | *Cymbella* | *Cymbella_delicatula* | Phototrophic |
| 46585 | TSAR | Stramenopiles | Bacillariophyceae | Bacillariophyceae | Cymbellales | Cymbellaceae | *Cymbella* | *Cymbella_fulva* | Phototrophic |
| 46634 | TSAR | Stramenopiles | Bacillariophyceae | Bacillariophyceae | Cymbellales | Cymbellaceae | *Cymbella* | *Cymbella_sp.* | Phototrophic |
| 46649 | TSAR | Stramenopiles | Bacillariophyceae | Bacillariophyceae | Cymbellales | Gomphonemataceae | *Encyonema* | *Encyonema_minutum* | Phototrophic |
| 46696 | TSAR | Stramenopiles | Bacillariophyceae | Bacillariophyceae | Cymbellales | Gomphonemataceae | *Gomphonema* | *Gomphonema_angustatum* | Phototrophic |
| 46700 | TSAR | Stramenopiles | Bacillariophyceae | Bacillariophyceae | Cymbellales | Gomphonemataceae | *Gomphonema* | *Gomphonema_micropus* | Phototrophic |
| 46137 | TSAR | Stramenopiles | Bacillariophyceae | Bacillariophyceae | Fragilariales | Fragilariaceae | *Synedra* | *Synedra_hyperborea* | Phototrophic |
| 46125 | TSAR | Stramenopiles | Bacillariophyceae | Bacillariophyceae | Fragilariales | Staurosiraceae | *Staurosira* | *Staurosira_mutabilis* | Phototrophic |
| 46127 | TSAR | Stramenopiles | Bacillariophyceae | Bacillariophyceae | Fragilariales | Staurosiraceae | *Staurosira* | *Staurosira_subsalina* | Phototrophic |
| 76560 | TSAR | Stramenopiles | Bacillariophyceae | Bacillariophyceae | Naviculales | Amphipleuraceae | *Halamphora* | *Halamphora_siqueirosii* | Phototrophic |
| 76821 | TSAR | Stramenopiles | Bacillariophyceae | Bacillariophyceae | Naviculales | Diadesmidaceae | *Luticola* | *Luticola_permuticopsis* | Phototrophic |
| 76979 | TSAR | Stramenopiles | Bacillariophyceae | Bacillariophyceae | Naviculales | Naviculaceae | *Navicula* | *Navicula_arenaria* | Phototrophic |
| 46741 | TSAR | Stramenopiles | Bacillariophyceae | Bacillariophyceae | Naviculales | Naviculaceae | *Navicula* | *Navicula_cryptotenella* | Phototrophic |
| 46749 | TSAR | Stramenopiles | Bacillariophyceae | Bacillariophyceae | Naviculales | Naviculaceae | *Navicula* | *Navicula_phyllepta* | Phototrophic |
| 46752 | TSAR | Stramenopiles | Bacillariophyceae | Bacillariophyceae | Naviculales | Naviculaceae | *Navicula* | *Navicula_radiosa* | Phototrophic |
| 46753 | TSAR | Stramenopiles | Bacillariophyceae | Bacillariophyceae | Naviculales | Naviculaceae | *Navicula* | *Navicula_reinhardtii* | Phototrophic |
| 76986 | TSAR | Stramenopiles | Bacillariophyceae | Bacillariophyceae | Naviculales | Naviculaceae | *Navicula* | *Navicula_salinarum* | Phototrophic |
| 46757 | TSAR | Stramenopiles | Bacillariophyceae | Bacillariophyceae | Naviculales | Naviculaceae | *Navicula* | *Navicula_sp.* | Phototrophic |
| 76991 | TSAR | Stramenopiles | Bacillariophyceae | Bacillariophyceae | Naviculales | Naviculaceae | *Navicula* | *Navicula_veneta* | Phototrophic |
| 46850 | TSAR | Stramenopiles | Bacillariophyceae | Bacillariophyceae | Naviculales | Naviculaceae | *Pseudogomphonema* | *Pseudogomphonema_sp.* | Phototrophic |
| 46890 | TSAR | Stramenopiles | Bacillariophyceae | Bacillariophyceae | Naviculales | Naviculaceae | *Seminavis* | *Seminavis_sp.* | Phototrophic |
| 46762 | TSAR | Stramenopiles | Bacillariophyceae | Bacillariophyceae | Naviculales | Naviculales_X | *Naviculales_XX* | *Naviculales_XX_sp.* | Phototrophic |
| 46885 | TSAR | Stramenopiles | Bacillariophyceae | Bacillariophyceae | Naviculales | Sellaphoraceae | *Sellaphora* | *Sellaphora_pupula* | Phototrophic |
| 46675 | TSAR | Stramenopiles | Bacillariophyceae | Bacillariophyceae | Naviculales | Stauroneidaceae | *Fistulifera* | *Fistulifera_pelliculosa* | Phototrophic |
| 46676 | TSAR | Stramenopiles | Bacillariophyceae | Bacillariophyceae | Naviculales | Stauroneidaceae | *Fistulifera* | *Fistulifera_saprophila* | Phototrophic |
| 46892 | TSAR | Stramenopiles | Bacillariophyceae | Bacillariophyceae | Naviculales | Stauroneidaceae | *Stauroneis* | *Stauroneis_anceps* | Phototrophic |
| 46036 | TSAR | Stramenopiles | Bacillariophyceae | Bacillariophyceae | Rhabdonematales | Tabellariaceae | *Diatoma* | *Diatoma_tenue* | Phototrophic |
| 46037 | TSAR | Stramenopiles | Bacillariophyceae | Bacillariophyceae | Rhabdonematales | Tabellariaceae | *Diatoma* | *Diatoma_tenuis* | Phototrophic |
| 46039 | TSAR | Stramenopiles | Bacillariophyceae | Bacillariophyceae | Rhabdonematales | Tabellariaceae | *Diatoma* | *Diatoma_vulgaris* | Phototrophic |
| 46145 | TSAR | Stramenopiles | Bacillariophyceae | Bacillariophyceae | Rhabdonematales | Tabellariaceae | *Tabellaria* | *Tabellaria_flocculosa* | Phototrophic |
| 46903 | TSAR | Stramenopiles | Bacillariophyceae | Bacillariophyceae | Surirellales | Surirellaceae | *Surirella* | *Surirella_sp.* | Phototrophic |
| 46485 | TSAR | Stramenopiles | Bacillariophyceae | Bacillariophyceae | Thalassiophysales | Catenulaceae | *Amphora* | *Amphora_capitellata* | Phototrophic |
| 46487 | TSAR | Stramenopiles | Bacillariophyceae | Bacillariophyceae | Thalassiophysales | Catenulaceae | *Amphora* | *Amphora_coffeaeformis* | Phototrophic |
| 46506 | TSAR | Stramenopiles | Bacillariophyceae | Bacillariophyceae | Thalassiophysales | Catenulaceae | *Amphora* | *Amphora_sp.* | Phototrophic |
| 47446 | TSAR | Stramenopiles | Bigyra | Bicoecea | Pseudodendromonadales | Pseudodendromonadales_X | *Adriamonas* | *Adriamonas_peritocrescens* | Bacterivore |
| 47448 | TSAR | Stramenopiles | Bigyra | Bicoecea | Pseudodendromonadales | Pseudodendromonadales_X | *Nerada* | *Nerada_mexicana* | Bacterivore |
| 47450 | TSAR | Stramenopiles | Bigyra | Bicoecea | Pseudodendromonadales | Pseudodendromonadales_X | *Pseudodendromonadales_XX* | *Pseudodendromonadales_XX_sp.* | Bacterivore |
| 47866 | TSAR | Stramenopiles | Bigyra | Opalozoa | Opalinata | Blastocystis-Group | *Blastocystis* | *Blastocystis_hominis* | Animal Parasite |
| 47528 | TSAR | Stramenopiles | Bigyra | Opalozoa | Opalozoa_X | MAST-12 | *MAST-12C* | *MAST-12C_sp.* | Animal Parasite |
| 74127 | TSAR | Stramenopiles | Bigyra | Sagenista | Labyrinthulomycetes | Amphifilaceae | *Amphifilaceae_X* | *Amphifilaceae_X_sp.* | Plant Parasite |
| 74129 | TSAR | Stramenopiles | Bigyra | Sagenista | Labyrinthulomycetes | Amphifilaceae | *Fibrophrys* | *Fibrophrys_sp.* | Plant Parasite |
| 74137 | TSAR | Stramenopiles | Bigyra | Sagenista | Labyrinthulomycetes | Labyrinthulaceae | *Aplanochytrium* | *Aplanochytrium_minutum* | Plant Parasite |
| 74138 | TSAR | Stramenopiles | Bigyra | Sagenista | Labyrinthulomycetes | Labyrinthulaceae | *Aplanochytrium* | *Aplanochytrium_sp.* | Plant Parasite |
| 74143 | TSAR | Stramenopiles | Bigyra | Sagenista | Labyrinthulomycetes | Labyrinthulaceae | *Labyrinthulaceae_ANT10_3* | *Labyrinthulaceae_ANT10_3_sp.* | Plant Parasite |
| 74164 | TSAR | Stramenopiles | Bigyra | Sagenista | Labyrinthulomycetes | Oblongichytridiaceae | *Oblongichytrium* | *Oblongichytrium_sp.* | Plant Parasite |
| 74167 | TSAR | Stramenopiles | Bigyra | Sagenista | Labyrinthulomycetes | Thraustochytriaceae | *Aurantiochytrium* | *Aurantiochytrium_sp.* | Plant Parasite |
| 74182 | TSAR | Stramenopiles | Bigyra | Sagenista | Labyrinthulomycetes | Thraustochytriaceae | *Thraustochytriaceae_SEK690* | *Thraustochytriaceae_SEK690_sp.* | Plant Parasite |
| 48648 | TSAR | Stramenopiles | Bolidophyceae | Bolidophyceae | Parmales | Parmales_env_2 | *Parmales_env_2_X* | *Parmales_env_2_X_sp.* | Eukaryvore |
| 74358 | TSAR | Stramenopiles | Chrysophyceae | Chrysophyceae | Chromulinales | Chromulinales_X | *Chromulinales_XX* | *Chromulinales_XX_sp.* | Mixotrophic |
| 74363 | TSAR | Stramenopiles | Chrysophyceae | Chrysophyceae | Chromulinales | Chrysosphaerellaceae | *Chrysosphaerella* | *Chrysosphaerella_brevispina* | Mixotrophic |
| 74365 | TSAR | Stramenopiles | Chrysophyceae | Chrysophyceae | Chromulinales | Chrysosphaerellaceae | *Chrysosphaerella* | *Chrysosphaerella_sp.* | Mixotrophic |
| 74366 | TSAR | Stramenopiles | Chrysophyceae | Chrysophyceae | Chrysophyceae_Clade-EC2H | Chrysophyceae_Clade-EC2H_X | *Chrysophyceae_Clade-EC2H_XX* | *Chrysophyceae_Clade-EC2H_XX_sp.* | Mixotrophic |
| 74368 | TSAR | Stramenopiles | Chrysophyceae | Chrysophyceae | Chrysophyceae_Cluster-I | Chrysophyceae_Cluster-I_X | *Chrysophyceae_Cluster-I_XX* | *Chrysophyceae_Cluster-I_XX_sp.* | Mixotrophic |
| 74369 | TSAR | Stramenopiles | Chrysophyceae | Chrysophyceae | Chrysophyceae_Cluster-II | Chrysophyceae_Cluster-II_X | *Chrysophyceae_Cluster-II_XX* | *Chrysophyceae_Cluster-II_XX_sp.* | Mixotrophic |
| 74370 | TSAR | Stramenopiles | Chrysophyceae | Chrysophyceae | Chrysophyceae_X | Chrysophyceae_XX | *Chrysastrella* | *Chrysastrella_breviappendiculata* | Mixotrophic |
| 74373 | TSAR | Stramenopiles | Chrysophyceae | Chrysophyceae | Chrysophyceae_X | Chrysophyceae_XX | *Chrysophyceae_XXX* | *Chrysophyceae_XXX_sp.* | Mixotrophic |
| 74376 | TSAR | Stramenopiles | Chrysophyceae | Chrysophyceae | Chrysosaccales | Chrysophyceae_Clade-E | *Chrysosaccales_XXX* | *Chrysosaccales_XXX_sp.* | Mixotrophic |
| 74378 | TSAR | Stramenopiles | Chrysophyceae | Chrysophyceae | Chrysosaccales | Chrysosaccaceae | *Chromophyton* | *Chromophyton_vischeri* | Mixotrophic |
| 74379 | TSAR | Stramenopiles | Chrysophyceae | Chrysophyceae | Chrysosaccales | Chrysosaccaceae | *Chrysosaccus* | *Chrysosaccus_sp.* | Mixotrophic |
| 74388 | TSAR | Stramenopiles | Chrysophyceae | Chrysophyceae | Hibberdiales | Hibberdiaceae | *Hibberdia* | *Hibberdia_magna* | Mixotrophic |
| 74395 | TSAR | Stramenopiles | Chrysophyceae | Chrysophyceae | Hydrurales | Hydruraceae | *Hydrurus* | *Hydrurus_foetidus* | Mixotrophic |
| 74398 | TSAR | Stramenopiles | Chrysophyceae | Chrysophyceae | Hydrurales | Hydrurales_Clade-II | *Hydrurales_Clade-II_X* | *Hydrurales_Clade-II_X_sp.* | Mixotrophic |
| 74400 | TSAR | Stramenopiles | Chrysophyceae | Chrysophyceae | Hydrurales | Hydrurales_X | *Hydrurales_XX* | *Hydrurales_XX_sp.* | Mixotrophic |
| 74405 | TSAR | Stramenopiles | Chrysophyceae | Chrysophyceae | Ochromonadales | Ochromonadaceae | *Chrysolepidomonas* | *Chrysolepidomonas_dendrolepidota* | Mixotrophic |
| 76125 | TSAR | Stramenopiles | Chrysophyceae | Chrysophyceae | Ochromonadales | Ochromonadaceae | *Chrysolepidomonas* | *Chrysolepidomonas_sp.* | Mixotrophic |
| 74408 | TSAR | Stramenopiles | Chrysophyceae | Chrysophyceae | Ochromonadales | Ochromonadaceae | *Dinobryon* | *Dinobryon_faculiferum* | Mixotrophic |
| 74418 | TSAR | Stramenopiles | Chrysophyceae | Chrysophyceae | Ochromonadales | Ochromonadaceae | *Dinobryon* | *Dinobryon_sp.* | Mixotrophic |
| 74432 | TSAR | Stramenopiles | Chrysophyceae | Chrysophyceae | Ochromonadales | Ochromonadaceae | *Spumella* | *Spumella_benthica* | Bacterivore |
| 74433 | TSAR | Stramenopiles | Chrysophyceae | Chrysophyceae | Ochromonadales | Ochromonadaceae | *Spumella* | *Spumella_bureschii* | Bacterivore |
| 74434 | TSAR | Stramenopiles | Chrysophyceae | Chrysophyceae | Ochromonadales | Ochromonadaceae | *Spumella* | *Spumella_communis* | Bacterivore |
| 74437 | TSAR | Stramenopiles | Chrysophyceae | Chrysophyceae | Ochromonadales | Ochromonadaceae | *Spumella* | *Spumella_longicolla* | Bacterivore |
| 74438 | TSAR | Stramenopiles | Chrysophyceae | Chrysophyceae | Ochromonadales | Ochromonadaceae | *Spumella* | *Spumella_oblata* | Bacterivore |
| 74442 | TSAR | Stramenopiles | Chrysophyceae | Chrysophyceae | Ochromonadales | Ochromonadaceae | *Spumella* | *Spumella_similis* | Bacterivore |
| 74443 | TSAR | Stramenopiles | Chrysophyceae | Chrysophyceae | Ochromonadales | Ochromonadaceae | *Spumella* | *Spumella_sinechrysos* | Bacterivore |
| 74444 | TSAR | Stramenopiles | Chrysophyceae | Chrysophyceae | Ochromonadales | Ochromonadaceae | *Spumella* | *Spumella_sp.* | Bacterivore |
| 74445 | TSAR | Stramenopiles | Chrysophyceae | Chrysophyceae | Ochromonadales | Ochromonadaceae | *Spumella* | *Spumella_vulgaris* | Bacterivore |
| 77900 | TSAR | Stramenopiles | Chrysophyceae | Chrysophyceae | Ochromonadales | Ochromonadaceae | *Uroglena* | *Uroglena_botrys* | Mixotrophic |
| 74449 | TSAR | Stramenopiles | Chrysophyceae | Chrysophyceae | Ochromonadales | Ochromonadaceae | *Uroglenopsis* | *Uroglenopsis_americana* | Mixotrophic |
| 74451 | TSAR | Stramenopiles | Chrysophyceae | Chrysophyceae | Ochromonadales | Ochromonadaceae | *Uroglenopsis* | *Uroglenopsis_sp.* | Mixotrophic |
| 74461 | TSAR | Stramenopiles | Chrysophyceae | Chrysophyceae | Ochromonadales | Ochromonadales_clade-VIII | *Ochromonadales_clade-VIII_X* | *Ochromonadales_clade-VIII_X_sp.* | Mixotrophic |
| 74464 | TSAR | Stramenopiles | Chrysophyceae | Chrysophyceae | Ochromonadales | Ochromonadales_clade-XII | *Ochromonadales_clade-XII_X* | *Ochromonadales_clade-XII_X_sp.* | Mixotrophic |
| 74457 | TSAR | Stramenopiles | Chrysophyceae | Chrysophyceae | Ochromonadales | Ochromonadales_X | *Ochromonadales_XX* | *Ochromonadales_XX_sp.* | Mixotrophic |
| 74468 | TSAR | Stramenopiles | Chrysophyceae | Chrysophyceae | Paraphysomonadales | Lepidochromonadaceae | *Lepidochromonas* | *Lepidochromonas_sp.* | Mixotrophic |
| 74475 | TSAR | Stramenopiles | Chrysophyceae | Chrysophyceae | Paraphysomonadales | Paraphysomonadaceae | *Paraphysomonas* | *Paraphysomonas_hebetispina* | Omnivore |
| 74488 | TSAR | Stramenopiles | Chrysophyceae | Chrysophyceae | Paraphysomonadales | Paraphysomonadaceae | *Paraphysomonas* | *Paraphysomonas_sp.* | Omnivore |
| 74494 | TSAR | Stramenopiles | Chrysophyceae | Chrysophyceae | Paraphysomonadales | Paraphysomonadales_Clade-EC1Ga | *Paraphysomonadales_Clade-EC1Ga_X* | *Paraphysomonadales_Clade-EC1Ga_X_sp.* | Omnivore |
| 74523 | TSAR | Stramenopiles | Chrysophyceae | Chrysophyceae | Synurales | Synuraceae | *Mallomonas* | *Mallomonas_mangofera* | Phototrophic |
| 74550 | TSAR | Stramenopiles | Chrysophyceae | Chrysophyceae | Synurales | Synuraceae | *Synura* | *Synura_curtispina* | Phototrophic |
| 46167 | TSAR | Stramenopiles | Diatomeae_X | Diatomeae_X | Diatomeae_XX | Phaeodactylaceae | *Phaeodactylum* | *Phaeodactylum_tricornutum* | Phototrophic |
| 47132 | TSAR | Stramenopiles | Dictyochophyceae | Dictyochophyceae | Dictyochophyceae_X | Pedinellales | *Pedinellales_X* | *Pedinellales_X_sp.* | Mixotrophic |
| 77466 | TSAR | Stramenopiles | Dictyochophyceae | Dictyochophyceae | Dictyochophyceae_X | Pedinellales | *Pteridomonas* | *Pteridomonas_sp.* | Mixotrophic |
| 76054 | TSAR | Stramenopiles | Eustigmatophyceae | Eustigmatophyceae | Eustigmatophyceae_X | Eustigmatophyceae_XX | *Characiopsis* | *Characiopsis_pernana* | Phototrophic |
| 47149 | TSAR | Stramenopiles | Eustigmatophyceae | Eustigmatophyceae | Eustigmatophyceae_X | Eustigmatophyceae_XX | *Eustigmatophyceae_XXX* | *Eustigmatophyceae_XXX_sp.* | Phototrophic |
| 47164 | TSAR | Stramenopiles | Eustigmatophyceae | Eustigmatophyceae | Eustigmatophyceae_X | Eustigmatophyceae_XX | *Nannochloropsis* | *Nannochloropsis_limnetica* | Mixotrophic |
| 47166 | TSAR | Stramenopiles | Eustigmatophyceae | Eustigmatophyceae | Eustigmatophyceae_X | Eustigmatophyceae_XX | *Nannochloropsis* | *Nannochloropsis_oceanica* | Mixotrophic |
| 47167 | TSAR | Stramenopiles | Eustigmatophyceae | Eustigmatophyceae | Eustigmatophyceae_X | Eustigmatophyceae_XX | *Nannochloropsis* | *Nannochloropsis_oculata* | Mixotrophic |
| 47169 | TSAR | Stramenopiles | Eustigmatophyceae | Eustigmatophyceae | Eustigmatophyceae_X | Eustigmatophyceae_XX | *Nannochloropsis* | *Nannochloropsis_sp.* | Mixotrophic |
| 47170 | TSAR | Stramenopiles | Eustigmatophyceae | Eustigmatophyceae | Eustigmatophyceae_X | Eustigmatophyceae_XX | *Pseudellipsoidion* | *Pseudellipsoidion_edaphicum* | Phototrophic |
| 47171 | TSAR | Stramenopiles | Eustigmatophyceae | Eustigmatophyceae | Eustigmatophyceae_X | Eustigmatophyceae_XX | *Pseudocharaciopsis* | *Pseudocharaciopsis_minuta* | Phototrophic |
| 47175 | TSAR | Stramenopiles | Eustigmatophyceae | Eustigmatophyceae | Eustigmatophyceae_X | Eustigmatophyceae_XX | *Pseudotetraedriella* | *Pseudotetraedriella_kamillae* | Phototrophic |
| 77796 | TSAR | Stramenopiles | Eustigmatophyceae | Eustigmatophyceae | Eustigmatophyceae_X | Eustigmatophyceae_XX | *Trachydiscus* | *Trachydiscus_sp.* | Mixotrophic |
| 75628 | TSAR | Stramenopiles | Eustigmatophyceae | Eustigmatophyceae | Eustigmatophyceae_X | Eustigmatophyceae_XX | *Vacuoliviride* | *Vacuoliviride_crystalliferum* | Phototrophic |
| 75646 | TSAR | Stramenopiles | Eustigmatophyceae | Sagenista | Labyrinthulomycetes | Amphifilaceae | *Sorodiplophrys* | *Sorodiplophrys_stercorea* | Plant Parasite |
| 47517 | TSAR | Stramenopiles | Gyrista_X | Gyrista_X | Gyrista_XX | MAST-1 | *MAST-1A* | *MAST-1A_sp.* | NA |
| 47519 | TSAR | Stramenopiles | Gyrista_X | Gyrista_X | Gyrista_XX | MAST-1 | *MAST-1C* | *MAST-1C_sp.* | NA |
| 47536 | TSAR | Stramenopiles | Gyrista_X | Gyrista_X | Gyrista_XX | MAST-2 | *MAST-2A* | *MAST-2A_sp.* | NA |
| 47539 | TSAR | Stramenopiles | Gyrista_X | Gyrista_X | Gyrista_XX | MAST-2 | *MAST-2D* | *MAST-2D_sp.* | NA |
| 46211 | TSAR | Stramenopiles | Mediophyceae | Mediophyceae | Chaetocerotales | Chaetocerotaceae | *Chaetoceros* | *Chaetoceros_neogracilis* | Phototrophic |
| 46223 | TSAR | Stramenopiles | Mediophyceae | Mediophyceae | Chaetocerotales | Chaetocerotaceae | *Chaetoceros* | *Chaetoceros_sp.* | Phototrophic |
| 46180 | TSAR | Stramenopiles | Mediophyceae | Mediophyceae | Mediophyceae_X | Mediophyceae_XX | *Mediophyceae_XXX* | *Mediophyceae_XXX_sp.* | Phototrophic |
| 46321 | TSAR | Stramenopiles | Mediophyceae | Mediophyceae | Thalassiosirales | Thalassiosiraceae | *Shionodiscus* | *Shionodiscus_ritscheri* | Phototrophic |
| 46359 | TSAR | Stramenopiles | Mediophyceae | Mediophyceae | Thalassiosirales | Thalassiosiraceae | *Thalassiosira* | *Thalassiosira_eccentrica* | Phototrophic |
| 46364 | TSAR | Stramenopiles | Mediophyceae | Mediophyceae | Thalassiosirales | Thalassiosiraceae | *Thalassiosira* | *Thalassiosira_hispida* | Phototrophic |
| 46370 | TSAR | Stramenopiles | Mediophyceae | Mediophyceae | Thalassiosirales | Thalassiosiraceae | *Thalassiosira* | *Thalassiosira_nordenskioeldii* | Phototrophic |
| 46378 | TSAR | Stramenopiles | Mediophyceae | Mediophyceae | Thalassiosirales | Thalassiosiraceae | *Thalassiosira* | *Thalassiosira_sp.* | Phototrophic |
| 47590 | TSAR | Stramenopiles | MOCH-2 | MOCH-2 | MOCH-2_X | MOCH-2_XX | *MOCH-2_XXX* | *MOCH-2_XXX_sp.* | NA |
| 47187 | TSAR | Stramenopiles | Pelagophyceae | Pelagophyceae | Pelagomonadales | Pelagomonadaceae | *Aureococcus* | *Aureococcus_anophagefferens* | Phototrophic |
| 70189 | TSAR | Stramenopiles | Pelagophyceae | Pelagophyceae | Pelagomonadales | Pelagomonadales_clade_A | *Pelagomonadales_clade_A_X* | *Pelagomonadales_clade_A_X_sp.* | Phototrophic |
| 70186 | TSAR | Stramenopiles | Pelagophyceae | Pelagophyceae | Pelagomonadales | Pelagomonadales_clade_A | *Pelagomonadales_clade_A1* | *Pelagomonadales_clade_A1_sp.* | Phototrophic |
| 47605 | TSAR | Stramenopiles | Peronosporomycetes | Peronosporomycetes | Peronosporomycetes_X | Haptoglossales | *Haptoglossa* | *Haptoglossa_sp.* | Pathogen |
| 47608 | TSAR | Stramenopiles | Peronosporomycetes | Peronosporomycetes | Peronosporomycetes_X | Leptomitales | *Leptomitales_X* | *Leptomitales_X_sp.* | Pathogen |
| 47609 | TSAR | Stramenopiles | Peronosporomycetes | Peronosporomycetes | Peronosporomycetes_X | Olpidophydiales | *Olpidiopsis* | *Olpidiopsis_porphyrae* | Pathogen |
| 47687 | TSAR | Stramenopiles | Peronosporomycetes | Peronosporomycetes | Peronosporomycetes_X | Peronosporales | *Lagenidium* | *Lagenidium_giganteum* | Pathogen |
| 47695 | TSAR | Stramenopiles | Peronosporomycetes | Peronosporomycetes | Peronosporomycetes_X | Peronosporales | *Peronosporales_X* | *Peronosporales_X_sp.* | Pathogen |
| 47816 | TSAR | Stramenopiles | Peronosporomycetes | Peronosporomycetes | Peronosporomycetes_X | Peronosporales | *Pythium* | *Pythium_grandisporangium* | Pathogen |
| 47824 | TSAR | Stramenopiles | Peronosporomycetes | Peronosporomycetes | Peronosporomycetes_X | Peronosporales | *Pythium* | *Pythium_nagaii* | Pathogen |
| 47847 | TSAR | Stramenopiles | Peronosporomycetes | Peronosporomycetes | Peronosporomycetes_X | Peronosporales | *Pythium* | *Pythium_rostratifingens* | Pathogen |
| 47836 | TSAR | Stramenopiles | Peronosporomycetes | Peronosporomycetes | Peronosporomycetes_X | Peronosporales | *Pythium* | *Pythium_senticosum* | Pathogen |
| 47839 | TSAR | Stramenopiles | Peronosporomycetes | Peronosporomycetes | Peronosporomycetes_X | Peronosporales | *Pythium* | *Pythium_takayamanum* | Pathogen |
| 47620 | TSAR | Stramenopiles | Peronosporomycetes | Peronosporomycetes | Peronosporomycetes_X | Peronosporomycetes_XX | *Peronosporomycetes_XXX* | *Peronosporomycetes_XXX_sp.* | Pathogen |
| 47849 | TSAR | Stramenopiles | Peronosporomycetes | Peronosporomycetes | Peronosporomycetes_X | Saprolegniales | *Achlya* | *Achlya_bisexualis* | Pathogen |
| 47850 | TSAR | Stramenopiles | Peronosporomycetes | Peronosporomycetes | Peronosporomycetes_X | Saprolegniales | *Aphanomyces* | *Aphanomyces_invadans* | Pathogen |
| 47614 | TSAR | Stramenopiles | Peronosporomycetes | Peronosporomycetes | Peronosporomycetes_X | Saprolegniales | *Aphanomyces* | *Aphanomyces_sp.* | Pathogen |
| 47860 | TSAR | Stramenopiles | Peronosporomycetes | Peronosporomycetes | Peronosporomycetes_X | Saprolegniales | *Pythiopsis* | *Pythiopsis_cymosa* | Pathogen |
| 46933 | TSAR | Stramenopiles | Synchromophyceae | Synchromophyceae | Synchromophyceae_X | Synchromophyceae_XX | *Synchromophyceae_XXX* | *Synchromophyceae_XXX_sp.* | Phototrophic |
| 47369 | TSAR | Stramenopiles | Xanthophyceae | Xanthophyceae | Xanthophyceae_X | Xanthophyceae_XX | *Botrydiopsis* | *Botrydiopsis_intercedens* | Phototrophic |
| 47373 | TSAR | Stramenopiles | Xanthophyceae | Xanthophyceae | Xanthophyceae_X | Xanthophyceae_XX | *Botryidiopsidaceae* | *Botryidiopsidaceae_sp.* | Phototrophic |
| 47374 | TSAR | Stramenopiles | Xanthophyceae | Xanthophyceae | Xanthophyceae_X | Xanthophyceae_XX | *Botryochloris* | *Botryochloris_sp.* | Phototrophic |
| 47381 | TSAR | Stramenopiles | Xanthophyceae | Xanthophyceae | Xanthophyceae_X | Xanthophyceae_XX | *Chlorellidium* | *Chlorellidium_tetrabotrys* | Phototrophic |
| 47383 | TSAR | Stramenopiles | Xanthophyceae | Xanthophyceae | Xanthophyceae_X | Xanthophyceae_XX | *Heterococcus* | *Heterococcus_caespitosus* | Phototrophic |
| 47385 | TSAR | Stramenopiles | Xanthophyceae | Xanthophyceae | Xanthophyceae_X | Xanthophyceae_XX | *Heterococcus* | *Heterococcus_fuornensis* | Phototrophic |
| 47389 | TSAR | Stramenopiles | Xanthophyceae | Xanthophyceae | Xanthophyceae_X | Xanthophyceae_XX | *Heterothrix* | *Heterothrix_debilis* | Phototrophic |
| 47390 | TSAR | Stramenopiles | Xanthophyceae | Xanthophyceae | Xanthophyceae_X | Xanthophyceae_XX | *Mischococcus* | *Mischococcus_sphaerocephalus* | Phototrophic |
| 47393 | TSAR | Stramenopiles | Xanthophyceae | Xanthophyceae | Xanthophyceae_X | Xanthophyceae_XX | *Ophiocytium* | *Ophiocytium_parvulum* | Phototrophic |
| 77100 | TSAR | Stramenopiles | Xanthophyceae | Xanthophyceae | Xanthophyceae_X | Xanthophyceae_XX | *Ophiocytium* | *Ophiocytium_sp.* | Phototrophic |
| 47394 | TSAR | Stramenopiles | Xanthophyceae | Xanthophyceae | Xanthophyceae_X | Xanthophyceae_XX | *Phaeobotrys* | *Phaeobotrys_solitaria* | Phototrophic |
| 47395 | TSAR | Stramenopiles | Xanthophyceae | Xanthophyceae | Xanthophyceae_X | Xanthophyceae_XX | *Pleurochloris* | *Pleurochloris_meiringensis* | Phototrophic |
| 47410 | TSAR | Stramenopiles | Xanthophyceae | Xanthophyceae | Xanthophyceae_X | Xanthophyceae_XX | *Vaucheria* | *Vaucheria_terrestris* | Phototrophic |
| 47411 | TSAR | Stramenopiles | Xanthophyceae | Xanthophyceae | Xanthophyceae_X | Xanthophyceae_XX | *Xanthonema* | *Xanthonema_bristolianum* | Phototrophic |
| 47412 | TSAR | Stramenopiles | Xanthophyceae | Xanthophyceae | Xanthophyceae_X | Xanthophyceae_XX | *Xanthonema* | *Xanthonema_sessile* | Phototrophic |
| 47413 | TSAR | Stramenopiles | Xanthophyceae | Xanthophyceae | Xanthophyceae_X | Xanthophyceae_XX | *Xanthonema* | *Xanthonema_sp.* | Phototrophic |
| 47415 | TSAR | Stramenopiles | Xanthophyceae | Xanthophyceae | Xanthophyceae_X | Xanthophyceae_XX | *Xanthophyceae_XXX* | *Xanthophyceae_XXX_sp.* | Phototrophic |
| 10349 | TSAR | Telonemia | Telonemia_X | Telonemia_XX | Telonemia_XXX | Telonemia-Group-1 | *Telonema-Group-1* | *Telonema_sp.* | Bacterivore |
| 10351 | TSAR | Telonemia | Telonemia_X | Telonemia_XX | Telonemia_XXX | Telonemia-Group-1 | *Telonemia-Group-1_X* | *Telonemia-Group-1_X_sp.* | Bacterivore |
| 10354 | TSAR | Telonemia | Telonemia_X | Telonemia_XX | Telonemia_XXX | Telonemia-Group-2 | *Telonemia-Group-2_X* | *Telonemia-Group-2_X_sp.* | Bacterivore |

**Supplementary Table4.** Protist and fungi taxa identified in freshwater, coastal, and terrestrial ecosystems. TaxID is the designated ID of the species in the PR2 (v5.0.0) reference database used for taxonomic assignment within Emu (Curry et al. 2022). Total read counts for each taxon within the three ecosystems is provided.

| **TaxID** | **Freshwater** | **Coastal** | **Terrestrial** | **Supergroup** | **Division** | **Subdivision** | **Class** | **Order** | **Family** | **Genus** | **Species** |
| --- | --- | --- | --- | --- | --- | --- | --- | --- | --- | --- | --- |
| 2819 | 96 | 566 | 97 | Amoebozoa | Discosea | Discosea_X | Flabellinia | Vannellida | Vannellidae | *Vannella* | *Vannella_sp.* |
| 3998 | 3053 | 33 | 649 | Archaeplastida | Chlorophyta | Chlorophyta_X | Chlorophyceae | Chaetopeltidales | Chaetopeltidaceae | *Planophila* | *Planophila_sp.* |
| 3243 | 12882 | 10345 | 1407 | Archaeplastida | Chlorophyta | Chlorophyta_X | Chlorophyceae | Chlamydomonadales | Chlamydomonadales_X | *Chlamydomonadales_XX* | *Chlamydomonadales_XX_sp.* |
| 3162 | 69 | 173 | 22 | Archaeplastida | Chlorophyta | Chlorophyta_X | Chlorophyceae | Chlamydomonadales | Chlamydomonadales_X | *Chlamydomonas* | *Chlamydomonas_raudensis* |
| 3169 | 5545 | 21315 | 1792 | Archaeplastida | Chlorophyta | Chlorophyta_X | Chlorophyceae | Chlamydomonadales | Chlamydomonadales_X | *Chlamydomonas* | *Chlamydomonas_sp.* |
| 3198 | 331 | 214 | 18 | Archaeplastida | Chlorophyta | Chlorophyta_X | Chlorophyceae | Chlamydomonadales | Chlamydomonadales_X | *Chlorococcum* | *Chlorococcum_sp.* |
| 76114 | 37 | 17 | 37 | Archaeplastida | Chlorophyta | Chlorophyta_X | Chlorophyceae | Chlamydomonadales | Chlamydomonadales_X | *Chloromonas* | *Chloromonas_krienitzii* |
| 3625 | 282 | 16 | 32 | Archaeplastida | Chlorophyta | Chlorophyta_X | Chlorophyceae | Sphaeropleales | Sphaeropleales_X | *Scenedesmus* | *Scenedesmus_sp.* |
| 3475 | 977 | 54 | 1000 | Archaeplastida | Chlorophyta | Chlorophyta_X | Trebouxiophyceae | Chlorellales | Chlorellales_X | *Chlorella* | *Chlorella_sp.* |
| 4352 | 174 | 342 | 364 | Archaeplastida | Chlorophyta | Chlorophyta_X | Ulvophyceae | Ulotrichales | Ulotrichales_X | *Hazenia* | *Hazenia_sp.* |
| 4366 | 49 | 1479 | 33 | Archaeplastida | Chlorophyta | Chlorophyta_X | Ulvophyceae | Ulotrichales | Ulotrichales_X | *Ulothrix* | *Ulothrix_sp.* |
| 71903 | 45 | 14 | 130 | Excavata | Discoba | Discoba_X | Heterolobosea | Tetramitia_V | Vahlkampfiidae_V | *Vahlkampfia* | *Vahlkampfia_avara* |
| 10636 | 16 | 23 | 10342 | Obazoa | Opisthokonta | Fungi | Ascomycota | Pezizomycotina | Dothideomycetes | *Dothideomycetes_X* | *Dothideomycetes_X_sp.* |
| 11329 | 256 | 270 | 739 | Obazoa | Opisthokonta | Fungi | Ascomycota | Pezizomycotina | Pezizomycotina_X | *Pezizomycotina_XX* | *Pezizomycotina_XX_sp.* |
| 17498 | 157 | 189 | 1374 | Obazoa | Opisthokonta | Fungi | Basidiomycota | Pucciniomycotina | Microbotryomycetes | *Glaciozyma* | *Glaciozyma_antarctica* |
| 76796 | 124 | 691 | 28 | Obazoa | Opisthokonta | Fungi | Basidiomycota | Pucciniomycotina | Microbotryomycetes | *Leucosporidium* | *Leucosporidium_sp.* |
| 74602 | 1840 | 3105 | 1499 | Obazoa | Opisthokonta | Fungi | Chytridiomycota | Chytridiales | Chytridiales_X | *Chytridiales_XX* | *Chytridiales_XX_sp.* |
| 74715 | 340 | 9704 | 803 | Obazoa | Opisthokonta | Fungi | Chytridiomycota | Rhizophydiales | Rhizophydiales_X | *Rhizophydiales_XX* | *Rhizophydiales_XX_sp.* |
| 74738 | 411 | 428 | 573 | Obazoa | Opisthokonta | Fungi | Chytridiomycota | Spizellomycetales | Spizellomycetales_X | *Spizellomycetales_XX* | *Spizellomycetales_XX_sp.* |
| 74634 | 392 | 250 | 1428 | Obazoa | Opisthokonta | Fungi | Fungi_X | Fungi_XX | Fungi_XXX | *Fungi_XXXX* | *Fungi_XXXX_sp.* |
| 18071 | 139871 | 3679 | 2738 | Obazoa | Opisthokonta | Fungi | Rozellomycota | Rozellomycota_X | Rozellomycota_XX | *Rozellomycota_XXX* | *Rozellomycota_XXX_sp.* |
| 49163 | 1582 | 10 | 106 | TSAR | Alveolata | Ciliophora | Heterotrichea | Heterotrichea_X | Blepharismidae | *Blepharisma* | *Blepharisma_sp.* |
| 698 | 243 | 30 | 64 | TSAR | Alveolata | Ciliophora | Litostomatea | Haptoria_6 | Lacrymariidae | *Lacrymariidae_X* | *Lacrymariidae_X_sp.* |
| 755 | 1949 | 129 | 871 | TSAR | Alveolata | Ciliophora | Litostomatea | Litostomatea_X | Litostomatea_XX | *Litostomatea_XXX* | *Litostomatea_XXX_sp.* |
| 75371 | 1609 | 26 | 59 | TSAR | Alveolata | Ciliophora | Nassophorea | Nassophorea_X | Nassulida | *Naxella* | *Naxella_paralucida* |
| 1180 | 13 | 73 | 909 | TSAR | Alveolata | Ciliophora | Phyllopharyngea | Cyrtophoria_4 | Chilodonellidae | *Chilodonellidae_X* | *Chilodonellidae_X_sp.* |
| 75406 | 0 | 18 | 1369 | TSAR | Alveolata | Ciliophora | Phyllopharyngea | Cyrtophoria_4 | Chilodonellidae | *Phascolodon* | *Phascolodon_vorticella* |
| 48679 | 47149 | 11692 | 77 | TSAR | Alveolata | Ciliophora | Spirotrichea | Choreotrichida | Strobilidiidae | *Strobilidiidae_X* | *Strobilidiidae_X_sp.* |
| 1512 | 22588 | 30 | 259 | TSAR | Alveolata | Ciliophora | Spirotrichea | Hypotrichia | Halteriidae | *Halteria* | *Halteria_grandinella* |
| 1518 | 252 | 13 | 102 | TSAR | Alveolata | Ciliophora | Spirotrichea | Hypotrichia | Holostichidae | *Anteholosticha* | *Anteholosticha_monilata* |
| 1544 | 1173 | 531 | 3089 | TSAR | Alveolata | Ciliophora | Spirotrichea | Hypotrichia | Hypotrichia_X | *Hypotrichia_XX* | *Hypotrichia_XX_sp.* |
| 1569 | 1857 | 54 | 676 | TSAR | Alveolata | Ciliophora | Spirotrichea | Hypotrichia | Oxytrichidae | *Bistichella* | *Bistichella_variabilis* |
| 1570 | 36 | 22 | 334 | TSAR | Alveolata | Ciliophora | Spirotrichea | Hypotrichia | Oxytrichidae | *Cyrtohymena* | *Cyrtohymena_citrina* |
| 1585 | 6800 | 1249 | 3367 | TSAR | Alveolata | Ciliophora | Spirotrichea | Hypotrichia | Oxytrichidae | *Orthamphisiella* | *Orthamphisiella_breviseries* |
| 1591 | 2852 | 143 | 3788 | TSAR | Alveolata | Ciliophora | Spirotrichea | Hypotrichia | Oxytrichidae | *Oxytricha* | *Oxytricha_longa* |
| 1592 | 190 | 21 | 517 | TSAR | Alveolata | Ciliophora | Spirotrichea | Hypotrichia | Oxytrichidae | *Oxytricha* | *Oxytricha_longigranulosa* |
| 1550 | 697 | 391 | 818 | TSAR | Alveolata | Ciliophora | Spirotrichea | Hypotrichia | Oxytrichidae | *Oxytricha* | *Oxytricha_sp.* |
| 1597 | 5631 | 178 | 16689 | TSAR | Alveolata | Ciliophora | Spirotrichea | Hypotrichia | Oxytrichidae | *Oxytrichidae_X* | *Oxytrichidae_X_sp.* |
| 75470 | 349 | 45 | 2699 | TSAR | Alveolata | Ciliophora | Spirotrichea | Hypotrichia | Pseudokeronopsidae | *Eschaneustyla* | *Eschaneustyla_lugeri* |
| 48728 | 121 | 24184 | 145 | TSAR | Alveolata | Ciliophora | Spirotrichea | Oligotrichida | Strombidiidae | *Strombidiidae_X* | *Strombidiidae_X_sp.* |
| 1765 | 2475 | 1042 | 98 | TSAR | Alveolata | Dinoflagellata | Dinophyceae | Dinophyceae_X | Dinophyceae_XX | *Dinophyceae_XXX* | *Dinophyceae_XXX_sp.* |
| 1927 | 5138 | 494 | 1 | TSAR | Alveolata | Dinoflagellata | Dinophyceae | Gymnodiniales | Gymnodiniaceae | *Gymnodinium* | *Gymnodinium_sp.* |
| 2035 | 8328 | 415 | 1 | TSAR | Alveolata | Dinoflagellata | Dinophyceae | Peridiniales | Peridiniaceae | *Peridinium* | *Peridinium_sp.* |
| 2211 | 161384 | 70 | 1 | TSAR | Alveolata | Dinoflagellata | Dinophyceae | Suessiales | Borghiellaceae | *Borghiella* | *Borghiella_tenuissima* |
| 2222 | 10672 | 598 | 1 | TSAR | Alveolata | Dinoflagellata | Dinophyceae | Suessiales | Suessiaceae | *Polarella* | *Polarella_glacialis* |
| 2397 | 108 | 114 | 92 | TSAR | Alveolata | Perkinsea | Perkinsida | Perkinsida_X | Perkinsida_XX | *Perkinsida_XXX* | *Perkinsida_XXX_sp.* |
| 45062 | 943 | 19 | 730 | TSAR | Rhizaria | Cercozoa | Endomyxa | Vampyrellida | Leptophryidae | *Leptophryidae_X* | *Leptophryidae_X_sp.* |
| 45070 | 272 | 142 | 95 | TSAR | Rhizaria | Cercozoa | Endomyxa | Vampyrellida | Penardia-lineage | *Penardia-lineage_X* | *Penardia-lineage_X_sp.* |
| 45075 | 20 | 24 | 118 | TSAR | Rhizaria | Cercozoa | Endomyxa | Vampyrellida | Thalassomyxa-lineage | *Thalassomyxa* | *Thalassomyxa_sp.* |
| 45076 | 49 | 110 | 253 | TSAR | Rhizaria | Cercozoa | Endomyxa | Vampyrellida | Vampyrellida_X | *Vampyrellida_clade_B* | *Vampyrellida_sp.* |
| 45203 | 679 | 216 | 46 | TSAR | Rhizaria | Cercozoa | Filosa-Imbricatea | Euglyphida | Euglyphida_X | *Euglyphida_XX* | *Euglyphida_XX_sp.* |
| 45262 | 118 | 189 | 1 | TSAR | Rhizaria | Cercozoa | Filosa-Imbricatea | Thaumatomonadida | Thaumatomonadida_X | *Thaumatomonadida_XX* | *Thaumatomonadida_XX_sp.* |
| 45360 | 387 | 1001 | 4030 | TSAR | Rhizaria | Cercozoa | Filosa-Sarcomonadea | Glissomonadida | Allapsidae | *Allapsidae_X* | *Allapsidae_X_sp.* |
| 45361 | 611 | 463 | 983 | TSAR | Rhizaria | Cercozoa | Filosa-Sarcomonadea | Glissomonadida | Allapsidae | *Group-Te* | *Group_Te_sp.* |
| 45374 | 1506 | 25 | 541 | TSAR | Rhizaria | Cercozoa | Filosa-Sarcomonadea | Glissomonadida | Glissomonadida_X | *Glissomonadida_XX* | *Glissomonadida_XX_sp.* |
| 45379 | 280 | 247 | 566 | TSAR | Rhizaria | Cercozoa | Filosa-Sarcomonadea | Glissomonadida | Sandonidae | *Flectomonas* | *Flectomonas_ekelundi* |
| 45386 | 12 | 56 | 310 | TSAR | Rhizaria | Cercozoa | Filosa-Sarcomonadea | Glissomonadida | Sandonidae | *Sandona* | *Sandona_aporians* |
| 45390 | 26 | 329 | 145 | TSAR | Rhizaria | Cercozoa | Filosa-Sarcomonadea | Glissomonadida | Sandonidae | *Sandona* | *Sandona_sp.* |
| 45391 | 401 | 430 | 891 | TSAR | Rhizaria | Cercozoa | Filosa-Sarcomonadea | Glissomonadida | Sandonidae | *Sandonidae_X* | *Sandonidae_X_sp.* |
| 45414 | 7022 | 3350 | 2 | TSAR | Rhizaria | Cercozoa | Filosa-Thecofilosea | Cryomonadida | Protaspa-lineage | *Protaspa-lineage_X* | *Protaspa-lineage_X_sp.* |
| 45431 | 277 | 7744 | 49903 | TSAR | Rhizaria | Cercozoa | Filosa-Thecofilosea | Cryomonadida | Rhogostoma-lineage | *Rhogostoma-lineage_X* | *Rhogostoma-lineage_X_sp.* |
| 45722 | 33 | 29 | 11 | TSAR | Rhizaria | Foraminifera | Monothalamids | Monothalamids_X | Lagynidae | *Nemogullmia* | *Nemogullmia_sp.* |
| 46513 | 4708 | 10119 | 296 | TSAR | Stramenopiles | Bacillariophyceae | Bacillariophyceae | Bacillariophyceae_X | Bacillariophyceae_XX | *Bacillariophyceae_XXX* | *Bacillariophyceae_XXX_sp.* |
| 46634 | 106 | 88 | 237 | TSAR | Stramenopiles | Bacillariophyceae | Bacillariophyceae | Cymbellales | Cymbellaceae | *Cymbella* | *Cymbella_sp.* |
| 46741 | 84 | 46 | 267 | TSAR | Stramenopiles | Bacillariophyceae | Bacillariophyceae | Naviculales | Naviculaceae | *Navicula* | *Navicula_cryptotenella* |
| 46749 | 3235 | 49792 | 123 | TSAR | Stramenopiles | Bacillariophyceae | Bacillariophyceae | Naviculales | Naviculaceae | *Navicula* | *Navicula_phyllepta* |
| 46757 | 448 | 1166 | 93 | TSAR | Stramenopiles | Bacillariophyceae | Bacillariophyceae | Naviculales | Naviculaceae | *Navicula* | *Navicula_sp.* |
| 74127 | 230 | 91 | 137 | TSAR | Stramenopiles | Bigyra | Sagenista | Labyrinthulomycetes | Amphifilaceae | *Amphifilaceae_X* | *Amphifilaceae_X_sp.* |
| 74129 | 522 | 462 | 129 | TSAR | Stramenopiles | Bigyra | Sagenista | Labyrinthulomycetes | Amphifilaceae | *Fibrophrys* | *Fibrophrys_sp.* |
| 74373 | 231 | 55 | 21 | TSAR | Stramenopiles | Chrysophyceae | Chrysophyceae | Chrysophyceae_X | Chrysophyceae_XX | *Chrysophyceae_XXX* | *Chrysophyceae_XXX_sp.* |
| 74395 | 71 | 400 | 1207 | TSAR | Stramenopiles | Chrysophyceae | Chrysophyceae | Hydrurales | Hydruraceae | *Hydrurus* | *Hydrurus_foetidus* |
| 74400 | 812 | 801 | 24 | TSAR | Stramenopiles | Chrysophyceae | Chrysophyceae | Hydrurales | Hydrurales_X | *Hydrurales_XX* | *Hydrurales_XX_sp.* |
| 74433 | 87566 | 384 | 3 | TSAR | Stramenopiles | Chrysophyceae | Chrysophyceae | Ochromonadales | Ochromonadaceae | *Spumella* | *Spumella_bureschii* |
| 74437 | 7799 | 2122 | 11 | TSAR | Stramenopiles | Chrysophyceae | Chrysophyceae | Ochromonadales | Ochromonadaceae | *Spumella* | *Spumella_longicolla* |
| 74442 | 1507 | 2815 | 158 | TSAR | Stramenopiles | Chrysophyceae | Chrysophyceae | Ochromonadales | Ochromonadaceae | *Spumella* | *Spumella_similis* |
| 74443 | 1802 | 2511 | 1 | TSAR | Stramenopiles | Chrysophyceae | Chrysophyceae | Ochromonadales | Ochromonadaceae | *Spumella* | *Spumella_sinechrysos* |
| 74444 | 295 | 614 | 521 | TSAR | Stramenopiles | Chrysophyceae | Chrysophyceae | Ochromonadales | Ochromonadaceae | *Spumella* | *Spumella_sp.* |
| 74457 | 528 | 3949 | 25 | TSAR | Stramenopiles | Chrysophyceae | Chrysophyceae | Ochromonadales | Ochromonadales_X | *Ochromonadales_XX* | *Ochromonadales_XX_sp.* |
| 47839 | 18 | 20 | 29 | TSAR | Stramenopiles | Peronosporomycetes | Peronosporomycetes | Peronosporomycetes_X | Peronosporales | *Pythium* | *Pythium_takayamanum* |
| 47415 | 144 | 814 | 1075 | TSAR | Stramenopiles | Xanthophyceae | Xanthophyceae | Xanthophyceae_X | Xanthophyceae_XX | *Xanthophyceae_XXX* | *Xanthophyceae_XXX_sp.* |

**Supplementary Table 5.** Protist and fungi taxa identified in all sampling sites, Ward Hunt Lake (WHL), Walker Hill, Quttinirpaaq Lagoon (QL), Ward Hunt Ice Shelf (WHIS), and Ellesmere Island (EI), which includes Antoniades Pond and Lake A Inflow. TaxID is the designated ID of the species in the PR2 (v5.0.0) reference database used for taxonomic assignment within Emu (Curry et al. 2022). Total read counts for each taxon within the three ecosystems is provided.

| **TaxID** | **WHL** | **Walker Hill** | **QL** | **WHIS** | **EI** | **Supergroup** | **Division** | **Subdivision** | **Class** | **Order** | **Family** | **Genus** | **Species** |
| --- | --- | --- | --- | --- | --- | --- | --- | --- | --- | --- | --- | --- | --- |
| 3243 | 1409 | 1407 | 10345 | 4884 | 6589 | Archaeplastida | Chlorophyta | Chlorophyta_X | Chlorophyceae | Chlamydomonadales | Chlamydomonadales_X | *Chlamydomonadales_XX* | *Chlamydomonadales_XX_sp.* |
| 3169 | 82 | 1792 | 21315 | 5260 | 203 | Archaeplastida | Chlorophyta | Chlorophyta_X | Chlorophyceae | Chlamydomonadales | Chlamydomonadales_X | *Chlamydomonas* | *Chlamydomonas_sp.* |
| 74602 | 1115 | 1499 | 3105 | 587 | 138 | Obazoa | Opisthokonta | Fungi | Chytridiomycota | Chytridiales | Chytridiales_X | *Chytridiales_XX* | *Chytridiales_XX_sp.* |
| 74715 | 51 | 803 | 9704 | 171 | 118 | Obazoa | Opisthokonta | Fungi | Chytridiomycota | Rhizophydiales | Rhizophydiales_X | *Rhizophydiales_XX* | *Rhizophydiales_XX_sp.* |
| 18071 | 137976 | 2738 | 3679 | 302 | 1593 | Obazoa | Opisthokonta | Fungi | Rozellomycota | Rozellomycota_X | Rozellomycota_XX | *Rozellomycota_XXX* | *Rozellomycota_XXX_sp.* |
| 49163 | 20 | 106 | 10 | 646 | 916 | TSAR | Alveolata | Ciliophora | Heterotrichea | Heterotrichea_X | Blepharismidae | *Blepharisma* | *Blepharisma_sp.* |
| 755 | 101 | 871 | 129 | 1438 | 410 | TSAR | Alveolata | Ciliophora | Litostomatea | Litostomatea_X | Litostomatea_XX | *Litostomatea_XXX* | *Litostomatea_XXX_sp.* |
| 1512 | 17145 | 259 | 30 | 4849 | 594 | TSAR | Alveolata | Ciliophora | Spirotrichea | Hypotrichia | Halteriidae | *Halteria* | *Halteria_grandinella* |
| 1518 | 14 | 102 | 13 | 135 | 103 | TSAR | Alveolata | Ciliophora | Spirotrichea | Hypotrichia | Holostichidae | *Anteholosticha* | *Anteholosticha_monilata* |
| 1544 | 642 | 3089 | 531 | 370 | 161 | TSAR | Alveolata | Ciliophora | Spirotrichea | Hypotrichia | Hypotrichia_X | *Hypotrichia_XX* | *Hypotrichia_XX_sp.* |
| 1569 | 119 | 676 | 54 | 384 | 1354 | TSAR | Alveolata | Ciliophora | Spirotrichea | Hypotrichia | Oxytrichidae | *Bistichella* | *Bistichella_variabilis* |
| 1585 | 27 | 3367 | 1249 | 4058 | 2715 | TSAR | Alveolata | Ciliophora | Spirotrichea | Hypotrichia | Oxytrichidae | *Orthamphisiella* | *Orthamphisiella_breviseries* |
| 1592 | 29 | 517 | 21 | 67 | 94 | TSAR | Alveolata | Ciliophora | Spirotrichea | Hypotrichia | Oxytrichidae | *Oxytricha* | *Oxytricha_longigranulosa* |
| 1550 | 106 | 818 | 391 | 405 | 186 | TSAR | Alveolata | Ciliophora | Spirotrichea | Hypotrichia | Oxytrichidae | *Oxytricha* | *Oxytricha_sp.* |
| 1597 | 2221 | 16689 | 178 | 1579 | 1831 | TSAR | Alveolata | Ciliophora | Spirotrichea | Hypotrichia | Oxytrichidae | *Oxytrichidae_X* | *Oxytrichidae_X_sp.* |
| 75470 | 48 | 2699 | 45 | 290 | 11 | TSAR | Alveolata | Ciliophora | Spirotrichea | Hypotrichia | Pseudokeronopsidae | *Eschaneustyla* | *Eschaneustyla_lugeri* |
| 48728 | 62 | 145 | 24184 | 32 | 27 | TSAR | Alveolata | Ciliophora | Spirotrichea | Oligotrichida | Strombidiidae | *Strombidiidae_X* | *Strombidiidae_X_sp.* |
| 1765 | 839 | 98 | 1042 | 375 | 1261 | TSAR | Alveolata | Dinoflagellata | Dinophyceae | Dinophyceae_X | Dinophyceae_XX | *Dinophyceae_XXX* | *Dinophyceae_XXX_sp.* |
| 1927 | 5002 | 1 | 494 | 22 | 114 | TSAR | Alveolata | Dinoflagellata | Dinophyceae | Gymnodiniales | Gymnodiniaceae | *Gymnodinium* | *Gymnodinium_sp.* |
| 2222 | 1243 | 1 | 598 | 8677 | 752 | TSAR | Alveolata | Dinoflagellata | Dinophyceae | Suessiales | Suessiaceae | *Polarella* | *Polarella_glacialis* |
| 45360 | 12 | 4030 | 1001 | 229 | 146 | TSAR | Rhizaria | Cercozoa | Filosa-Sarcomonadea | Glissomonadida | Allapsidae | *Allapsidae_X* | *Allapsidae_X_sp.* |
| 45361 | 12 | 983 | 463 | 466 | 133 | TSAR | Rhizaria | Cercozoa | Filosa-Sarcomonadea | Glissomonadida | Allapsidae | *Group-Te* | *Group_Te_sp.* |
| 45391 | 51 | 891 | 430 | 241 | 109 | TSAR | Rhizaria | Cercozoa | Filosa-Sarcomonadea | Glissomonadida | Sandonidae | *Sandonidae_X* | *Sandonidae_X_sp.* |
| 74129 | 14 | 129 | 462 | 337 | 171 | TSAR | Stramenopiles | Bigyra | Sagenista | Labyrinthulomycetes | Amphifilaceae | *Fibrophrys* | *Fibrophrys_sp.* |

**Supplementary Table 6.** Contribution of terrestrial source microbial communities: soil, soil crust, water track microbial mat (WT mat), and water track microplankton (WT water) to Ward Hunt microplankton communities. Contribution provided as an estimated percentage of total reads per taxon, only taxa that were identified to have a terrestrial input were included. Taxa ordered by highest percentage of reads contributed from terrestrial sources (sum of all source percentages).

| **TaxID** | **Soil (%)** | **Soil Crust (%)** | **WT Mat (%)** | **WT Water (%)** | **Unknown (%)** | **Terrestrial (%)** | **Division** | **Subdivision** | **Class** | **Order** | **Family** | **Genus** | **Species** |
| --- | --- | --- | --- | --- | --- | --- | --- | --- | --- | --- | --- | --- | --- |
| 45431 | 98.8 | 0.0 | 0.0 | 0.0 | 1.3 | 98.8 | Rhizaria | Cercozoa | Filosa-Thecofilosea | Cryomonadida | *Rhogostoma-lineage* | *Rhogostoma-lineage_X* | *Rhogostoma-lineage_X_sp.* |
| 18071 | 98.0 | 0.0 | 0.0 | 0.0 | 2.0 | 98.0 | Opisthokonta | Fungi | Rozellomycota | Rozellomycota_X | *Rozellomycota_XX* | *Rozellomycota_XXX* | *Rozellomycota_XXX_sp.* |
| 1597 | 97.7 | 0.0 | 0.0 | 0.0 | 2.2 | 97.8 | Alveolata | Ciliophora | Spirotrichea | Hypotrichia | *Oxytrichidae* | *Oxytrichidae_X* | *Oxytrichidae_X_sp.* |
| 45360 | 97.5 | 0.0 | 0.0 | 0.0 | 2.5 | 97.5 | Rhizaria | Cercozoa | Filosa-Sarcomonadea | Glissomonadida | *Allapsidae* | *Allapsidae_X* | *Allapsidae_X_sp.* |
| 74373 | 91.2 | 0.0 | 0.0 | 0.0 | 8.8 | 91.2 | Stramenopiles | Gyrista | Chrysophyceae | Chrysophyceae_X | *Chrysophyceae_XX* | *Chrysophyceae_XXX* | *Chrysophyceae_XXX_sp.* |
| 1512 | 90.4 | 0.0 | 0.0 | 0.0 | 9.6 | 90.4 | Alveolata | Ciliophora | Spirotrichea | Hypotrichia | *Halteriidae* | *Halteria* | *Halteria_grandinella* |
| 3243 | 88.4 | 0.0 | 0.0 | 0.0 | 11.6 | 88.4 | Chlorophyta | Chlorophyta_X | Chlorophyceae | Chlamydomonadales | *Chlamydomonadales_X* | *Chlamydomonadales_XX* | *Chlamydomonadales_XX_sp.* |
| 1592 | 79.3 | 0.0 | 0.0 | 0.0 | 20.7 | 79.3 | Alveolata | Ciliophora | Spirotrichea | Hypotrichia | *Oxytrichidae* | *Oxytricha* | *Oxytricha_longigranulosa* |
| 48679 | 79.0 | 0.0 | 0.0 | 0.0 | 21.0 | 79.0 | Alveolata | Ciliophora | Spirotrichea | Choreotrichida | *Strobilidiidae* | *Strobilidiidae_X* | *Strobilidiidae_X_sp.* |
| 3169 | 71.8 | 0.0 | 0.0 | 0.0 | 28.2 | 71.8 | Chlorophyta | Chlorophyta_X | Chlorophyceae | Chlamydomonadales | *Chlamydomonadales_X* | *Chlamydomonas* | *Chlamydomonas_sp.* |
| 975 | 67.5 | 0.0 | 0.0 | 0.0 | 32.5 | 67.5 | Alveolata | Ciliophora | Oligohymenophorea | Peritrichia_2 | *Sessilida* | *Opisthonecta* | *Opisthonecta_minima* |
| 1544 | 61.3 | 0.0 | 0.0 | 0.1 | 38.5 | 61.5 | Alveolata | Ciliophora | Spirotrichea | Hypotrichia | *Hypotrichia_X* | *Hypotrichia_XX* | *Hypotrichia_XX_sp.* |
| 74444 | 52.8 | 0.0 | 0.0 | 0.0 | 47.2 | 52.8 | Stramenopiles | Gyrista | Chrysophyceae | Ochromonadales | *Ochromonadaceae* | *Spumella* | *Spumella_sp.* |
| 74457 | 52.6 | 0.0 | 0.0 | 0.0 | 47.4 | 52.6 | Stramenopiles | Gyrista | Chrysophyceae | Ochromonadales | *Ochromonadales_X* | *Ochromonadales_XX* | *Ochromonadales_XX_sp.* |
| 74602 | 51.5 | 0.0 | 0.0 | 0.2 | 48.3 | 51.7 | Opisthokonta | Fungi | Chytridiomycota | Chytridiales | *Chytridiales_X* | *Chytridiales_XX* | *Chytridiales_XX_sp.* |
| 45414 | 50.5 | 0.0 | 0.0 | 0.0 | 49.5 | 50.5 | Rhizaria | Cercozoa | Filosa-Thecofilosea | Cryomonadida | *Protaspa-lineage* | *Protaspa-lineage_X* | *Protaspa-lineage_X_sp.* |
| 45391 | 49.7 | 0.0 | 0.0 | 0.0 | 50.3 | 49.7 | Rhizaria | Cercozoa | Filosa-Sarcomonadea | Glissomonadida | *Sandonidae* | *Sandonidae_X* | *Sandonidae_X_sp.* |
| 75470 | 46.2 | 0.0 | 0.0 | 0.0 | 53.8 | 46.2 | Alveolata | Ciliophora | Spirotrichea | Hypotrichia | *Pseudokeronopsidae* | *Eschaneustyla* | *Eschaneustyla_lugeri* |
| 74433 | 43.0 | 0.0 | 0.0 | 0.0 | 57.0 | 43.0 | Stramenopiles | Gyrista | Chrysophyceae | Ochromonadales | *Ochromonadaceae* | *Spumella* | *Spumella_bureschii* |
| 49305 | 35.6 | 0.0 | 0.0 | 0.2 | 64.1 | 35.9 | Alveolata | Ciliophora | Oligohymenophorea | Peritrichia_2 | *Sessilida* | *Sessilida_X* | *Sessilida_X_sp.* |
| 2222 | 35.7 | 0.0 | 0.0 | 0.0 | 64.3 | 35.7 | Alveolata | Dinoflagellata | Dinophyceae | Suessiales | *Suessiaceae* | *Polarella* | *Polarella_glacialis* |
| 755 | 34.9 | 0.0 | 0.0 | 0.0 | 65.1 | 34.9 | Alveolata | Ciliophora | Litostomatea | Litostomatea_X | *Litostomatea_XX* | *Litostomatea_XXX* | *Litostomatea_XXX_sp.* |
| 74443 | 34.3 | 0.0 | 0.0 | 0.0 | 65.7 | 34.3 | Stramenopiles | Gyrista | Chrysophyceae | Ochromonadales | *Ochromonadaceae* | *Spumella* | *Spumella_sinechrysos* |
| 2035 | 33.7 | 0.0 | 0.0 | 0.0 | 66.3 | 33.7 | Alveolata | Dinoflagellata | Dinophyceae | Peridiniales | *Peridiniaceae* | *Peridinium* | *Peridinium_sp.* |
| 74494 | 33.5 | 0.0 | 0.0 | 0.0 | 66.5 | 33.5 | Stramenopiles | Gyrista | Chrysophyceae | Paraphysomonadales | *Paraphysomonadales_Clade-EC1Ga* | *Paraphysomonadales_Clade-EC1Ga_X* | *Paraphysomonadales_Clade-EC1Ga_X_sp.* |
| 1927 | 33.3 | 0.0 | 0.0 | 0.0 | 66.7 | 33.3 | Alveolata | Dinoflagellata | Dinophyceae | Gymnodiniales | *Gymnodiniaceae* | *Gymnodinium* | *Gymnodinium_sp.* |
| 2211 | 32.3 | 0.0 | 0.0 | 0.0 | 67.7 | 32.3 | Alveolata | Dinoflagellata | Dinophyceae | Suessiales | *Borghiellaceae* | *Borghiella* | *Borghiella_tenuissima* |
| 1646 | 0.0 | 0.0 | 0.0 | 1.4 | 98.6 | 1.4 | Alveolata | Ciliophora | Spirotrichea | Hypotrichia | *Spirofilidae* | *Pseudouroleptus* | *Pseudouroleptus_caudatus* |
| 74395 | 0.0 | 0.0 | 0.0 | 0.6 | 99.4 | 0.6 | Stramenopiles | Gyrista | Chrysophyceae | Hydrurales | *Hydruraceae* | *Hydrurus* | *Hydrurus_foetidus* |
| 77900 | 0.4 | 0.0 | 0.0 | 0.0 | 99.6 | 0.4 | Stramenopiles | Gyrista | Chrysophyceae | Ochromonadales | *Ochromonadaceae* | *Uroglena* | *Uroglena_botrys* |
| 48728 | 0.2 | 0.0 | 0.2 | 0.0 | 99.7 | 0.3 | Alveolata | Ciliophora | Spirotrichea | Oligotrichida | *Strombidiidae* | *Strombidiidae_X* | *Strombidiidae_X_sp.* |
| 10125 | 0.3 | 0.0 | 0.0 | 0.0 | 99.7 | 0.3 | Cryptophyta | Cryptophyta_X | Cryptophyceae | Cryptomonadales | *Cryptomonadales_X* | *Cryptomonas* | *Cryptomonas_sp.* |
| 1550 | 0.0 | 0.0 | 0.0 | 0.3 | 99.7 | 0.3 | Alveolata | Ciliophora | Spirotrichea | Hypotrichia | *Oxytrichidae* | *Oxytricha* | *Oxytricha_sp.* |
| 1569 | 0.1 | 0.0 | 0.1 | 0.1 | 99.7 | 0.3 | Alveolata | Ciliophora | Spirotrichea | Hypotrichia | *Oxytrichidae* | *Bistichella* | *Bistichella_variabilis* |
| 5412 | 0.2 | 0.0 | 0.0 | 0.0 | 99.8 | 0.2 | Streptophyta | Streptophyta_X | Charophyceae | Charophyceae_X | *Charophyceae_XX* | *Chara* | *Chara_braunii* |
| 2170 | 0.2 | 0.0 | 0.0 | 0.0 | 99.8 | 0.2 | Alveolata | Dinoflagellata | Dinophyceae | Peridiniales | *Thoracosphaeraceae* | *Thoracosphaeraceae_X* | *Thoracosphaeraceae_X_sp.* |
| 698 | 0.1 | 0.0 | 0.0 | 0.1 | 99.8 | 0.2 | Alveolata | Ciliophora | Litostomatea | Haptoria_6 | *Lacrymariidae* | *Lacrymariidae_X* | *Lacrymariidae_X_sp.* |
| 45460 | 0.2 | 0.0 | 0.0 | 0.0 | 99.8 | 0.2 | Rhizaria | Cercozoa | Novel-clade-10-12 | Novel-clade-10 | *Novel-clade-10_X* | *Novel-clade-10_XX* | *Novel-clade-10_XX_sp.* |
| 49141 | 0.1 | 0.0 | 0.0 | 0.0 | 99.9 | 0.1 | Alveolata | Ciliophora | Colpodea | Colpodea_X | *Bursariomorphida* | *Bursariomorphida_X* | *Bursariomorphida_X_sp.* |
| 515 | 0.1 | 0.0 | 0.0 | 0.0 | 99.9 | 0.1 | Alveolata | Ciliophora | Colpodea | Colpodea_X | *Bursariomorphida* | *Bryometopus* | *Bryometopus_pseudochilodon* |
| 74365 | 0.1 | 0.0 | 0.0 | 0.0 | 99.9 | 0.1 | Stramenopiles | Gyrista | Chrysophyceae | Chromulinales | *Chrysosphaerellaceae* | *Chrysosphaerella* | *Chrysosphaerella_sp.* |
| 74442 | 0.1 | 0.0 | 0.0 | 0.0 | 99.9 | 0.1 | Stramenopiles | Gyrista | Chrysophyceae | Ochromonadales | *Ochromonadaceae* | *Spumella* | *Spumella_similis* |
| 47536 | 0.1 | 0.0 | 0.0 | 0.0 | 99.9 | 0.1 | Stramenopiles | Gyrista | Gyrista_X | Gyrista_XX | *MAST-2* | *MAST-2A* | *MAST-2A_sp.* |
| 2321 | 0.1 | 0.0 | 0.0 | 0.0 | 99.9 | 0.1 | Alveolata | Dinoflagellata | Syndiniales | Dino-Group-II | *Dino-Group-II-Clade-21* | *Dino-Group-II-Clade-21_X* | *Dino-Group-II-Clade-21_X_sp.* |
| 74434 | 0.1 | 0.0 | 0.0 | 0.0 | 99.9 | 0.1 | Stramenopiles | Gyrista | Chrysophyceae | Ochromonadales | *Ochromonadaceae* | *Spumella* | *Spumella_communis* |
| 3237 | 0.1 | 0.0 | 0.0 | 0.0 | 99.9 | 0.1 | Chlorophyta | Chlorophyta_X | Chlorophyceae | Chlamydomonadales | *Chlamydomonadales_X* | *Chloromonas* | *Chloromonas_sp.* |
| 1765 | 0.1 | 0.0 | 0.0 | 0.0 | 99.9 | 0.1 | Alveolata | Dinoflagellata | Dinophyceae | Dinophyceae_X | *Dinophyceae_XX* | *Dinophyceae_XXX* | *Dinophyceae_XXX_sp.* |
| 74438 | 0.1 | 0.0 | 0.0 | 0.0 | 99.9 | 0.1 | Stramenopiles | Gyrista | Chrysophyceae | Ochromonadales | *Ochromonadaceae* | *Spumella* | *Spumella_oblata* |
| 514 | 0.1 | 0.0 | 0.0 | 0.0 | 99.9 | 0.1 | Alveolata | Ciliophora | Colpodea | Colpodea_X | *Bursariomorphida* | *Bryometopus* | *Bryometopus_atypicus* |
| 45237 | 0.1 | 0.0 | 0.0 | 0.0 | 99.9 | 0.1 | Rhizaria | Cercozoa | Filosa-Imbricatea | Filosa-Imbricatea_X | *Novel-clade-2* | *Novel-clade-2_X* | *Novel-clade-2_X_sp.* |
| 74449 | 0.1 | 0.0 | 0.0 | 0.0 | 99.9 | 0.1 | Stramenopiles | Gyrista | Chrysophyceae | Ochromonadales | *Ochromonadaceae* | *Uroglenopsis* | *Uroglenopsis_americana* |
| 74432 | 0.1 | 0.0 | 0.0 | 0.0 | 99.9 | 0.1 | Stramenopiles | Gyrista | Chrysophyceae | Ochromonadales | *Ochromonadaceae* | *Spumella* | *Spumella_benthica* |
| 49161 | 0.1 | 0.0 | 0.0 | 0.0 | 99.9 | 0.1 | Alveolata | Ciliophora | Prostomatea | Prorodontida | *Urotrichidae* | *Urotricha* | *Urotricha_sp.* |
| 10354 | 0.1 | 0.0 | 0.0 | 0.0 | 99.9 | 0.1 | Telonemia | Telonemia_X | Telonemia_XX | Telonemia_XXX | *Telonemia-Group-2* | *Telonemia-Group-2_X* | *Telonemia-Group-2_X_sp.* |
| 74370 | 0.1 | 0.0 | 0.0 | 0.0 | 99.9 | 0.1 | Stramenopiles | Gyrista | Chrysophyceae | Chrysophyceae_X | *Chrysophyceae_XX* | *Chrysastrella* | *Chrysastrella_breviappendiculata* |
| 74369 | 0.1 | 0.0 | 0.0 | 0.0 | 99.9 | 0.1 | Stramenopiles | Gyrista | Chrysophyceae | Chrysophyceae_Cluster-II | *Chrysophyceae_Cluster-II_X* | *Chrysophyceae_Cluster-II_XX* | *Chrysophyceae_Cluster-II_XX_sp.* |
| 10103 | 0.1 | 0.0 | 0.0 | 0.0 | 99.9 | 0.1 | Cryptophyta | Cryptophyta_X | Cryptophyceae | Cryptomonadales | *Cryptomonadales_X* | *Cryptomonadales_XX* | *Cryptomonadales_XX_sp.* |
| 74437 | 0.1 | 0.0 | 0.0 | 0.0 | 99.9 | 0.1 | Stramenopiles | Gyrista | Chrysophyceae | Ochromonadales | *Ochromonadaceae* | *Spumella* | *Spumella_longicolla* |

**Supplementary Table 7.** Contribution of terrestrial source microbial communities: soil, soil crust, water track microbial mat (WT mat), and water track microplankton (WT water) to Quttinirpaaq Lagoon microbial mat communities. Contribution provided as an estimated percentage of total reads per taxon, only taxa that were identified to have a terrestrial input were included. Taxa ordered by highest percentage of reads contributed from terrestrial sources (sum of all source percentages).

| **TaxID** | **Soil (%)** | **Soil Crust (%)** | **WT Mat (%)** | **WT Water (%)** | **Unknown (%)** | **Terrestrial (%)** | **Division** | **Subdivision** | **Class** | **Order** | **Family** | **Genus** | **Species** |
| --- | --- | --- | --- | --- | --- | --- | --- | --- | --- | --- | --- | --- | --- |
| 1597 | 7.8 | 60.6 | 19.7 | 3.7 | 8.2 | 91.8 | Alveolata | Ciliophora | Spirotrichea | Hypotrichia | *Oxytrichidae* | *Oxytrichidae_X* | *Oxytrichidae_X_sp.* |
| 12049 | 0.0 | 19.2 | 72.5 | 0.0 | 8.3 | 91.7 | Opisthokonta | Fungi | Ascomycota | Pezizomycotina | *Eurotiomycetes* | *Knufia* | *Knufia_perforans* |
| 45431 | 7.9 | 21.2 | 60.5 | 1.7 | 8.7 | 91.3 | Rhizaria | Cercozoa | Filosa-Thecofilosea | Cryomonadida | *Rhogostoma-lineage* | *Rhogostoma-lineage_X* | *Rhogostoma-lineage_X_sp.* |
| 4016 | 0.0 | 83.3 | 2.7 | 0.0 | 14.0 | 86.0 | Chlorophyta | Chlorophyta_X | Trebouxiophyceae | Prasiolales | *Prasiolales_X* | *Pseudostichococcus* | *Pseudostichococcus_monallantoides* |
| 18071 | 16.0 | 58.7 | 4.3 | 0.2 | 20.9 | 79.1 | Opisthokonta | Fungi | Rozellomycota | Rozellomycota_X | *Rozellomycota_XX* | *Rozellomycota_XXX* | *Rozellomycota_XXX_sp.* |
| 10636 | 0.0 | 47.0 | 29.1 | 0.0 | 23.9 | 76.1 | Opisthokonta | Fungi | Ascomycota | Pezizomycotina | *Dothideomycetes* | *Dothideomycetes_X* | *Dothideomycetes_X_sp.* |
| 3169 | 0.8 | 73.9 | 0.4 | 0.0 | 24.9 | 75.1 | Chlorophyta | Chlorophyta_X | Chlorophyceae | Chlamydomonadales | *Chlamydomonadales_X* | *Chlamydomonas* | *Chlamydomonas_sp.* |
| 45360 | 28.1 | 21.8 | 15.2 | 3.5 | 31.5 | 68.5 | Rhizaria | Cercozoa | Filosa-Sarcomonadea | Glissomonadida | *Allapsidae* | *Allapsidae_X* | *Allapsidae_X_sp.* |
| 1585 | 0.0 | 34.9 | 17.3 | 5.3 | 42.5 | 57.5 | Alveolata | Ciliophora | Spirotrichea | Hypotrichia | *Oxytrichidae* | *Orthamphisiella* | *Orthamphisiella_breviseries* |
| 1544 | 1.3 | 29.3 | 14.2 | 11.0 | 44.1 | 55.9 | Alveolata | Ciliophora | Spirotrichea | Hypotrichia | *Hypotrichia_X* | *Hypotrichia_XX* | *Hypotrichia_XX_sp.* |
| 3243 | 4.3 | 46.8 | 4.2 | 0.6 | 44.1 | 55.9 | Chlorophyta | Chlorophyta_X | Chlorophyceae | Chlamydomonadales | *Chlamydomonadales_X* | *Chlamydomonadales_XX* | *Chlamydomonadales_XX_sp.* |
| 75470 | 0.4 | 35.8 | 17.6 | 1.6 | 44.7 | 55.3 | Alveolata | Ciliophora | Spirotrichea | Hypotrichia | *Pseudokeronopsidae* | *Eschaneustyla* | *Eschaneustyla_lugeri* |
| 4026 | 0.0 | 51.2 | 3.6 | 0.0 | 45.2 | 54.8 | Chlorophyta | Chlorophyta_X | Trebouxiophyceae | Prasiolales | *Prasiolales_X* | *Stichococcus* | *Stichococcus_bacillaris* |
| 18176 | 25.8 | 8.8 | 19.3 | 0.0 | 46.2 | 53.8 | Opisthokonta | Fungi | Mucoromycota | Mucoromycotina | *Mucoromycotina_X* | *Mucoromycotina_XX* | *Mucoromycotina_XX_sp.* |
| 74602 | 0.8 | 30.9 | 6.1 | 11.1 | 51.1 | 48.9 | Opisthokonta | Fungi | Chytridiomycota | Chytridiales | *Chytridiales_X* | *Chytridiales_XX* | *Chytridiales_XX_sp.* |
| 3902 | 0.0 | 40.0 | 8.3 | 0.0 | 51.7 | 48.3 | Chlorophyta | Chlorophyta_X | Trebouxiophyceae | Chlorellales | *Chlorellales_X* | *Neocystis* | *Neocystis_brevis* |
| 74634 | 0.0 | 6.4 | 2.1 | 38.4 | 53.1 | 46.9 | Opisthokonta | Fungi | Fungi_X | Fungi_XX | *Fungi_XXX* | *Fungi_XXXX* | *Fungi_XXXX_sp.* |
| 755 | 0.2 | 37.1 | 3.3 | 6.0 | 53.4 | 46.6 | Alveolata | Ciliophora | Litostomatea | Litostomatea_X | *Litostomatea_XX* | *Litostomatea_XXX* | *Litostomatea_XXX_sp.* |
| 1591 | 0.0 | 1.8 | 30.2 | 13.8 | 54.1 | 45.9 | Alveolata | Ciliophora | Spirotrichea | Hypotrichia | *Oxytrichidae* | *Oxytricha* | *Oxytricha_longa* |
| 11329 | 0.0 | 41.1 | 3.2 | 0.0 | 55.7 | 44.3 | Opisthokonta | Fungi | Ascomycota | Pezizomycotina | *Pezizomycotina_X* | *Pezizomycotina_XX* | *Pezizomycotina_XX_sp.* |
| 45391 | 1.2 | 27.9 | 6.5 | 0.0 | 64.4 | 35.6 | Rhizaria | Cercozoa | Filosa-Sarcomonadea | Glissomonadida | *Sandonidae* | *Sandonidae_X* | *Sandonidae_X_sp.* |
| 1569 | 0.0 | 26.5 | 3.9 | 4.3 | 65.4 | 34.6 | Alveolata | Ciliophora | Spirotrichea | Hypotrichia | *Oxytrichidae* | *Bistichella* | *Bistichella_variabilis* |
| 3998 | 0.0 | 23.3 | 5.5 | 0.0 | 71.2 | 28.8 | Chlorophyta | Chlorophyta_X | Chlorophyceae | Chaetopeltidales | *Chaetopeltidaceae* | *Planophila* | *Planophila_sp.* |
| 45361 | 12.7 | 5.7 | 9.4 | 0.0 | 72.2 | 27.8 | Rhizaria | Cercozoa | Filosa-Sarcomonadea | Glissomonadida | *Allapsidae* | *Group-Te* | *Group_Te_sp.* |
| 75406 | 0.0 | 11.7 | 13.9 | 0.0 | 74.4 | 25.6 | Alveolata | Ciliophora | Phyllopharyngea | Cyrtophoria_4 | *Chilodonellidae* | *Phascolodon* | *Phascolodon_vorticella* |
| 45062 | 12.1 | 3.7 | 6.8 | 0.0 | 77.4 | 22.6 | Rhizaria | Cercozoa | Endomyxa | Vampyrellida | *Leptophryidae* | *Leptophryidae_X* | *Leptophryidae_X_sp.* |
| 74715 | 0.0 | 14.3 | 5.2 | 1.9 | 78.7 | 21.3 | Opisthokonta | Fungi | Chytridiomycota | Rhizophydiales | *Rhizophydiales_X* | *Rhizophydiales_XX* | *Rhizophydiales_XX_sp.* |
| 1570 | 0.0 | 10.9 | 1.4 | 8.2 | 79.5 | 20.5 | Alveolata | Ciliophora | Spirotrichea | Hypotrichia | *Oxytrichidae* | *Cyrtohymena* | *Cyrtohymena_citrina* |
| 75371 | 16.2 | 3.8 | 0.4 | 0.0 | 79.6 | 20.4 | Alveolata | Ciliophora | Nassophorea | Nassophorea_X | *Nassulida* | *Naxella* | *Naxella_paralucida* |
| 45374 | 0.0 | 16.0 | 4.0 | 0.0 | 80.0 | 20.0 | Rhizaria | Cercozoa | Filosa-Sarcomonadea | Glissomonadida | *Glissomonadida_X* | *Glissomonadida_XX* | *Glissomonadida_XX_sp.* |
| 45379 | 0.0 | 14.2 | 5.7 | 0.0 | 80.2 | 19.8 | Rhizaria | Cercozoa | Filosa-Sarcomonadea | Glissomonadida | *Sandonidae* | *Flectomonas* | *Flectomonas_ekelundi* |
| 1550 | 0.0 | 0.0 | 6.9 | 12.1 | 81.0 | 19.0 | Alveolata | Ciliophora | Spirotrichea | Hypotrichia | *Oxytrichidae* | *Oxytricha* | *Oxytricha_sp.* |
| 1765 | 0.0 | 18.6 | 0.0 | 0.0 | 81.4 | 18.6 | Alveolata | Dinoflagellata | Dinophyceae | Dinophyceae_X | *Dinophyceae_XX* | *Dinophyceae_XXX* | *Dinophyceae_XXX_sp.* |
| 17498 | 0.0 | 0.0 | 18.5 | 0.0 | 81.5 | 18.5 | Opisthokonta | Fungi | Basidiomycota | Pucciniomycotina | *Microbotryomycetes* | *Glaciozyma* | *Glaciozyma_antarctica* |
| 1522 | 0.0 | 18.2 | 0.0 | 0.0 | 81.8 | 18.2 | Alveolata | Ciliophora | Spirotrichea | Hypotrichia | *Holostichidae* | *Anteholosticha* | *Anteholosticha_sp.* |
| 74738 | 1.9 | 5.4 | 5.5 | 4.7 | 82.6 | 17.4 | Opisthokonta | Fungi | Chytridiomycota | Spizellomycetales | *Spizellomycetales_X* | *Spizellomycetales_XX* | *Spizellomycetales_XX_sp.* |
| 74444 | 0.9 | 5.3 | 4.2 | 5.3 | 84.2 | 15.8 | Stramenopiles | Gyrista | Chrysophyceae | Ochromonadales | *Ochromonadaceae* | *Spumella* | *Spumella_sp.* |
| 47415 | 0.0 | 3.3 | 12.3 | 0.0 | 84.5 | 15.5 | Stramenopiles | Gyrista | Xanthophyceae | Xanthophyceae_X | *Xanthophyceae_XX* | *Xanthophyceae_XXX* | *Xanthophyceae_XXX_sp.* |
| 2397 | 0.0 | 8.9 | 0.0 | 5.8 | 85.3 | 14.7 | Alveolata | Perkinsea | Perkinsida | Perkinsida_X | *Perkinsida_XX* | *Perkinsida_XXX* | *Perkinsida_XXX_sp.* |
| 3475 | 0.0 | 0.0 | 14.6 | 0.0 | 85.4 | 14.6 | Chlorophyta | Chlorophyta_X | Trebouxiophyceae | Chlorellales | *Chlorellales_X* | *Chlorella* | *Chlorella_sp.* |
| 4352 | 0.0 | 10.7 | 3.7 | 0.0 | 85.6 | 14.4 | Chlorophyta | Chlorophyta_X | Ulvophyceae | Ulotrichales | *Ulotrichales_X* | *Hazenia* | *Hazenia_sp.* |
| 47413 | 0.0 | 0.0 | 13.8 | 0.0 | 86.2 | 13.8 | Stramenopiles | Gyrista | Xanthophyceae | Xanthophyceae_X | *Xanthophyceae_XX* | *Xanthonema* | *Xanthonema_sp.* |
| 74603 | 0.0 | 0.0 | 0.0 | 13.5 | 86.5 | 13.5 | Opisthokonta | Fungi | Chytridiomycota | Chytridiomycota_X | *Chytridiomycota_XX* | *Chytridiomycota_XXX* | *Chytridiomycota_XXX_sp.* |
| 4344 | 0.0 | 12.4 | 0.0 | 0.0 | 87.6 | 12.4 | Chlorophyta | Chlorophyta_X | Ulvophyceae | Ulotrichales | *Ulotrichales_X* | *Chlorothrix* | *Chlorothrix_sp.* |
| 47389 | 0.0 | 0.0 | 12.1 | 0.0 | 87.9 | 12.1 | Stramenopiles | Gyrista | Xanthophyceae | Xanthophyceae_X | *Xanthophyceae_XX* | *Heterothrix* | *Heterothrix_debilis* |
| 3947 | 0.0 | 9.4 | 1.7 | 0.0 | 88.9 | 11.1 | Chlorophyta | Chlorophyta_X | Trebouxiophyceae | Prasiolales | *Prasiolales_X* | *Stichococcus* | *Stichococcus_sp.* |
| 1180 | 0.0 | 0.0 | 10.4 | 0.0 | 89.6 | 10.4 | Alveolata | Ciliophora | Phyllopharyngea | Cyrtophoria_4 | *Chilodonellidae* | *Chilodonellidae_X* | *Chilodonellidae_X_sp.* |
| 49163 | 0.0 | 8.0 | 0.0 | 2.0 | 90.0 | 10.0 | Alveolata | Ciliophora | Heterotrichea | Heterotrichea_X | *Blepharismidae* | *Blepharisma* | *Blepharisma_sp.* |
| 45203 | 0.0 | 9.8 | 0.0 | 0.0 | 90.2 | 9.8 | Rhizaria | Cercozoa | Filosa-Imbricatea | Euglyphida | *Euglyphida_X* | *Euglyphida_XX* | *Euglyphida_XX_sp.* |
| 46700 | 0.0 | 0.0 | 9.1 | 0.0 | 90.9 | 9.1 | Stramenopiles | Gyrista | Bacillariophyceae | Cymbellales | *Gomphonemataceae* | *Gomphonema* | *Gomphonema_micropus* |
| 13637 | 0.0 | 7.1 | 1.9 | 0.0 | 91.0 | 9.0 | Opisthokonta | Fungi | Ascomycota | Pezizomycotina | *Pezizomycotina_X* | *Eiglera* | *Eiglera_flavida* |
| 76997 | 0.0 | 0.0 | 9.0 | 0.0 | 91.0 | 9.0 | Rhizaria | Cercozoa | Filosa-Sarcomonadea | Cercomonadida | *Cercomonadidae* | *Neocercomonas* | *Neocercomonas_sp.* |
| 74127 | 1.1 | 5.2 | 1.9 | 0.0 | 91.9 | 8.1 | Stramenopiles | Bigyra | Sagenista | Labyrinthulomycetes | *Amphifilaceae* | *Amphifilaceae_X* | *Amphifilaceae_X_sp.* |
| 45386 | 0.0 | 3.2 | 4.5 | 0.0 | 92.3 | 7.7 | Rhizaria | Cercozoa | Filosa-Sarcomonadea | Glissomonadida | *Sandonidae* | *Sandona* | *Sandona_aporians* |
| 46634 | 0.0 | 1.8 | 4.8 | 0.0 | 93.4 | 6.6 | Stramenopiles | Gyrista | Bacillariophyceae | Cymbellales | *Cymbellaceae* | *Cymbella* | *Cymbella_sp.* |
| 4366 | 0.0 | 6.6 | 0.0 | 0.0 | 93.4 | 6.6 | Chlorophyta | Chlorophyta_X | Ulvophyceae | Ulotrichales | *Ulotrichales_X* | *Ulothrix* | *Ulothrix_sp.* |
| 3162 | 0.0 | 5.1 | 0.0 | 0.0 | 94.9 | 5.1 | Chlorophyta | Chlorophyta_X | Chlorophyceae | Chlamydomonadales | *Chlamydomonadales_X* | *Chlamydomonas* | *Chlamydomonas_raudensis* |
| 46741 | 0.0 | 0.0 | 5.0 | 0.0 | 95.0 | 5.0 | Stramenopiles | Gyrista | Bacillariophyceae | Naviculales | *Naviculaceae* | *Navicula* | *Navicula_cryptotenella* |
| 2968 | 1.0 | 0.0 | 4.0 | 0.0 | 95.0 | 5.0 | Tubulinea | Tubulinea_X | Elardia | Euamoebida | *Nolandellidae* | *Nolandellidae_X* | *Nolandellidae_X_sp.* |
| 74442 | 0.0 | 0.0 | 0.5 | 4.3 | 95.2 | 4.8 | Stramenopiles | Gyrista | Chrysophyceae | Ochromonadales | *Ochromonadaceae* | *Spumella* | *Spumella_similis* |
| 74143 | 0.0 | 4.7 | 0.0 | 0.0 | 95.3 | 4.7 | Stramenopiles | Bigyra | Sagenista | Labyrinthulomycetes | *Labyrinthulaceae* | *Labyrinthulaceae_ANT10_3* | *Labyrinthulaceae_ANT10_3_sp.* |
| 46757 | 0.0 | 3.5 | 1.0 | 0.0 | 95.5 | 4.5 | Stramenopiles | Gyrista | Bacillariophyceae | Naviculales | *Naviculaceae* | *Navicula* | *Navicula_sp.* |
| 3198 | 0.0 | 3.9 | 0.0 | 0.0 | 96.1 | 3.9 | Chlorophyta | Chlorophyta_X | Chlorophyceae | Chlamydomonadales | *Chlamydomonadales_X* | *Chlorococcum* | *Chlorococcum_sp.* |
| 46749 | 0.0 | 3.2 | 0.2 | 0.0 | 96.5 | 3.5 | Stramenopiles | Gyrista | Bacillariophyceae | Naviculales | *Naviculaceae* | *Navicula* | *Navicula_phyllepta* |
| 5412 | 0.0 | 0.0 | 3.3 | 0.0 | 96.7 | 3.3 | Streptophyta | Streptophyta_X | Charophyceae | Charophyceae_X | *Charophyceae_XX* | *Chara* | *Chara_braunii* |
| 45076 | 0.0 | 0.0 | 3.3 | 0.0 | 96.7 | 3.3 | Rhizaria | Cercozoa | Endomyxa | Vampyrellida | *Vampyrellida_X* | *Vampyrellida_clade_B* | *Vampyrellida_sp.* |
| 1518 | 0.0 | 1.5 | 1.5 | 0.0 | 96.9 | 3.1 | Alveolata | Ciliophora | Spirotrichea | Hypotrichia | *Holostichidae* | *Anteholosticha* | *Anteholosticha_monilata* |
| 46513 | 0.0 | 0.0 | 2.9 | 0.0 | 97.1 | 2.9 | Stramenopiles | Gyrista | Bacillariophyceae | Bacillariophyceae_X | *Bacillariophyceae_XX* | *Bacillariophyceae_XXX* | *Bacillariophyceae_XXX_sp.* |
| 71903 | 0.0 | 0.0 | 2.9 | 0.0 | 97.1 | 2.9 | Discoba | Discoba_X | Heterolobosea | Tetramitia_V | *Vahlkampfiidae_V* | *Vahlkampfia* | *Vahlkampfia_avara* |
| 47381 | 0.0 | 0.0 | 2.6 | 0.0 | 97.4 | 2.6 | Stramenopiles | Gyrista | Xanthophyceae | Xanthophyceae_X | *Xanthophyceae_XX* | *Chlorellidium* | *Chlorellidium_tetrabotrys* |
| 48728 | 0.0 | 1.8 | 0.6 | 0.0 | 97.6 | 2.4 | Alveolata | Ciliophora | Spirotrichea | Oligotrichida | *Strombidiidae* | *Strombidiidae_X* | *Strombidiidae_X_sp.* |
| 47383 | 0.0 | 0.0 | 2.1 | 0.0 | 97.9 | 2.1 | Stramenopiles | Gyrista | Xanthophyceae | Xanthophyceae_X | *Xanthophyceae_XX* | *Heterococcus* | *Heterococcus_caespitosus* |
| 46676 | 0.0 | 0.0 | 2.0 | 0.0 | 97.9 | 2.1 | Stramenopiles | Gyrista | Bacillariophyceae | Naviculales | *Stauroneidaceae* | *Fistulifera* | *Fistulifera_saprophila* |
| 74129 | 0.0 | 0.0 | 1.9 | 0.0 | 98.1 | 1.9 | Stramenopiles | Bigyra | Sagenista | Labyrinthulomycetes | *Amphifilaceae* | *Fibrophrys* | *Fibrophrys_sp.* |
| 45390 | 0.0 | 0.0 | 1.9 | 0.0 | 98.1 | 1.9 | Rhizaria | Cercozoa | Filosa-Sarcomonadea | Glissomonadida | *Sandonidae* | *Sandona* | *Sandona_sp.* |
| 46675 | 0.0 | 0.0 | 1.7 | 0.0 | 98.3 | 1.7 | Stramenopiles | Gyrista | Bacillariophyceae | Naviculales | *Stauroneidaceae* | *Fistulifera* | *Fistulifera_pelliculosa* |
| 45414 | 1.6 | 0.0 | 0.0 | 0.0 | 98.4 | 1.6 | Rhizaria | Cercozoa | Filosa-Thecofilosea | Cryomonadida | *Protaspa-lineage* | *Protaspa-lineage_X* | *Protaspa-lineage_X_sp.* |
| 17505 | 0.0 | 0.0 | 1.5 | 0.0 | 98.5 | 1.5 | Opisthokonta | Fungi | Basidiomycota | Pucciniomycotina | *Microbotryomycetes* | *Microbotryomycetes_X* | *Microbotryomycetes_X_sp.* |
| 45075 | 0.0 | 0.0 | 1.3 | 0.0 | 98.8 | 1.3 | Rhizaria | Cercozoa | Endomyxa | Vampyrellida | *Thalassomyxa-lineage* | *Thalassomyxa* | *Thalassomyxa_sp.* |
| 2819 | 0.0 | 0.0 | 1.2 | 0.0 | 98.8 | 1.2 | Discosea | Discosea_X | Flabellinia | Vannellida | *Vannellidae* | *Vannella* | *Vannella_sp.* |
| 45070 | 0.0 | 0.0 | 1.1 | 0.0 | 98.9 | 1.1 | Rhizaria | Cercozoa | Endomyxa | Vampyrellida | *Penardia-lineage* | *Penardia-lineage_X* | *Penardia-lineage_X_sp.* |
| 47839 | 0.0 | 0.0 | 1.0 | 0.0 | 99.0 | 1.0 | Stramenopiles | Gyrista | Peronosporomycetes | Peronosporomycetes_X | *Peronosporales* | *Pythium* | *Pythium_takayamanum* |
| 45722 | 0.0 | 0.0 | 0.9 | 0.0 | 99.1 | 0.9 | Rhizaria | Foraminifera | Monothalamids | Monothalamids_X | *Lagynidae* | *Nemogullmia* | *Nemogullmia_sp.* |
| 2222 | 0.8 | 0.0 | 0.0 | 0.0 | 99.2 | 0.8 | Alveolata | Dinoflagellata | Dinophyceae | Suessiales | *Suessiaceae* | *Polarella* | *Polarella_glacialis* |
| 1732 | 0.0 | 0.7 | 0.0 | 0.0 | 99.3 | 0.7 | Alveolata | Ciliophora | Spirotrichea | Spirotrichea_X | *Spirotrichea_XX* | *Phacodinium* | *Phacodinium_metchnikoffi* |
| 76796 | 0.0 | 0.0 | 0.6 | 0.0 | 99.4 | 0.6 | Opisthokonta | Fungi | Basidiomycota | Pucciniomycotina | *Microbotryomycetes* | *Leucosporidium* | *Leucosporidium_sp.* |
| 74400 | 0.0 | 0.0 | 0.5 | 0.0 | 99.5 | 0.5 | Stramenopiles | Gyrista | Chrysophyceae | Hydrurales | *Hydrurales_X* | *Hydrurales_XX* | *Hydrurales_XX_sp.* |
| 45262 | 0.4 | 0.0 | 0.0 | 0.0 | 99.6 | 0.4 | Rhizaria | Cercozoa | Filosa-Imbricatea | Thaumatomonadida | *Thaumatomonadida_X* | *Thaumatomonadida_XX* | *Thaumatomonadida_XX_sp.* |
| 47412 | 0.0 | 0.0 | 0.3 | 0.0 | 99.7 | 0.3 | Stramenopiles | Gyrista | Xanthophyceae | Xanthophyceae_X | *Xanthophyceae_XX* | *Xanthonema* | *Xanthonema_sessile* |
| 3704 | 0.0 | 0.0 | 0.0 | 0.0 | 100.0 | 0.0 | Chlorophyta | Chlorophyta_X | Mamiellophyceae | Mamiellales | *Mamiellaceae* | *Mantoniella* | *Mantoniella_squamata* |
| 4369 | 0.0 | 0.0 | 0.0 | 0.0 | 100.0 | 0.0 | Chlorophyta | Chlorophyta_X | Ulvophyceae | Ulotrichales | *Ulotrichales_X* | *Urospora* | *Urospora_neglecta* |
| 74138 | 0.0 | 0.0 | 0.0 | 0.0 | 100.0 | 0.0 | Stramenopiles | Bigyra | Sagenista | Labyrinthulomycetes | *Labyrinthulaceae* | *Aplanochytrium* | *Aplanochytrium_sp.* |

Supplementary Figure 1

**Supplementary Figure 1.** Rarefaction curves of sample read depth against number of unique eukaryotic taxa in the (A) microbial mat, (B) microplankton, (C) soil crust, and (D) soil samples. AP (Pink) = Antoniades Pond, LA Inflow (Grey) = Lake A inflow stream, QL (Dark Blue) = Quttinirpaaq Lagoon, WHIS (Light Blue) = Ward Hunt Ice Shelf, WT (Yellow) = Walker Hill, WHL (Green) = Ward Hunt Lake.

Supplementary Figure 2

**Supplementary Figure 2.** Depth profile of Ward Hunt Lake. (A) Temperature (°C), (B) dissolved oxygen saturation (%), (C) specific conductivity (μS cm^-1^), and (D) chlorophyll α (μg L^-1^). Ice cover represented on graphs to approximate depth of 1.5m.

Supplementary Figure 3

**Supplementary Figure 3.** Canonical correspondence analysis (CCA) of the microbial eukaryotic (protist and fungi) diversity within microbial mat and microplankton communities, plotted alongside water temperature (Temp), specific conductivity, and pH, variables significantly associated with distribution of taxa between communities by permutational multivariate analysis of variance (PERMANOVA). Length and direction of arrows demonstrate strength of association with the communities.

Supplementary Figure 4

**Supplementary Figure 4.** Diversity of (A) Fungi and (B) Metazoa assigned reads averaged by sampling location and represented as relative abundance of total 18S rRNA gene reads. Reads grouped at the Class level
